# Synthesis of novel carbazole hydrazine-carbothioamide scaffold as potent antioxidant, anticancer and antimicrobial agents

**DOI:** 10.1186/s13065-024-01207-1

**Published:** 2024-05-21

**Authors:** İrfan Çapan, Mohammed Hawash, Mohammed T. Qaoud, Levent Gülüm, Ezgi Nurdan Yenilmez Tunoglu, Kezban Uçar Çifci, Bekir Sıtkı Çevrimli, Yusuf Sert, Süleyman Servi, İrfan Koca, Yusuf Tutar

**Affiliations:** 1 Department of Pharmaceutical Basic Sciences, Faculty of Pharmacy, Gazi University, 06330 Ankara, Türkiye; 2 Sente Kimya Research and Development Inc., 06200 Ankara, Türkiye; 3 Department of Pharmacy, Faculty of Medicine and Health Sciences, An-Najah National University, Nablus, Palestine; 4 Department of Pharmacy, Faculty of Pharmacy, Cyprus International University, Northern Cyprus, Mersin 10, 99258 Nicosia, Türkiye; 5 Department of Plant and Animal Production, Mudurnu Süreyya Astarcı Vocational College, Bolu Abant İzzet Baysal University, Bolu, Türkiye; 6 Department of Medical Laboratory Techniques, Vocational School of Health Services, Demiroğlu Bilim University, Istanbul, Türkiye; 7 Department of Molecular Medicine, Faculty of Health Sciences, University of Health Sciences, Istanbul, Türkiye; 8 Division of Basic Sciences and Health, Hemp Research Institute, Yozgat Bozok University, Yozgat, Türkiye; 9 Department of Chemistry and Chemical Processing Technologies, Technical Sciences Vocational College, Gazi University, Ankara, Türkiye; 10 Sorgun Vocational College, Yozgat Bozok University, Yozgat, Türkiye; 11 Department of Chemistry, Faculty of Science, Fırat University, Elazığ, Türkiye; 12 Department of Chemistry, Faculty of Art & Sciences, Yozgat Bozok University, Yozgat, Türkiye; 13 Medical School, Division of Biochemistry, Recep Tayyip Erdogan University, Rize, Türkiye; 14 Faculty of Pharmacy, Division of Biochemistry, University of Health Sciences, Istanbul, Türkiye

**Keywords:** Carbazole, Thiosemicarbazide, Antioxidant, Anticancer, PI3K/Akt/mTOR pathway, Molecular docking

## Abstract

**Background:**

Carbazole-based molecules containing thiosemicarbazide functional groups are recognized for their diverse biological activities, particularly in enhancing therapeutic anticancer effects through inhibiting crucial pathways. These derivatives also exhibit noteworthy antioxidant properties.

**Objectives:**

This study aims to synthesize, characterize, and evaluate the antioxidant and anticancer activities of 18 novel carbazole derivatives.

**Methods:**

The radical scavenging capabilities of the compounds were assessed using the 2,2-diphenyl-1-picrylhydrazyl assay. Antiproliferative activities were evaluated on MCF-7 cancer cell lines through viability assays. Additionally, the modulation of the PI3K/Akt/mTOR pathway, apoptosis/necrosis induction, and cell cycle analysis were conducted for the most promising anticancer agents.

**Results:**

nine compounds showed potent antioxidant activities with IC_50_ values lower than the positive control acarbose, with compounds **4 h** and **4y** exhibiting the highest potency (IC_50_ values of 0.73 and 0.38 µM, respectively). Furthermore, compounds **4o** and **4r** displayed significant anticancer effects, with IC_50_ values of 2.02 and 4.99 µM, respectively. Compound **4o**, in particular, exhibited promising activity by targeting the PI3K/Akt/mTOR signaling pathway, inhibiting tumor survival, inducing apoptosis, and causing cell cycle arrest in MCF-7 cell lines. Furthermore, compound **4o** was showed significant antimicrobial activities against *S. aureus* and *E. coli,* and antifungal effect against *C. albicans.* Its potential to overcome drug resistance through this pathway inhibition highlights its promise as an anticancer agent. Molecular docking simulations supported these findings, revealing favorable binding profiles and interactions within the active sites of the enzymes PI3K, AKT1, and mTOR. Moreover, assessing the druggability of the newly synthesized thiosemicarbazide derivatives demonstrated optimal physicochemical properties, further endorsing their potential as drug candidates.

**Supplementary Information:**

The online version contains supplementary material available at 10.1186/s13065-024-01207-1.

## Introduction

Heterocyclic compounds, which serve as scaffolds for synthesizing novel medications, are a highly significant category of molecules that have garnered considerable interest in many biological investigations [1–3]. Among these potential chemotherapeutic agents, heterocyclic compounds that target cancer cells hold significant promise [4, 5]. Carbazole, in particular, occupies a prominent position within this class of compounds. Both natural and synthetic carbazole derivatives have exhibited a wide range of beneficial properties such as anti-cancer [6–10], anti-HIV [11–13], anti-inflammatory [14], anti-viral, anti-microbial [15–17], and anti-histamine activities [18].

Moreover, carbazole derivatives have demonstrated various other biological activities, including anti-serotonin, diuretic [19], anti-fungicide, anti-inflammatory inhibitor, and anti-convulsant activities [20]. Importantly, these derivatives have drawn attention as effective pharmaceutical agents in the treatment of breast cancer, kidney cancer, brain tumors, and leukemia, with their anti-tumor and anti-HIV activity properties. Notably, their minimal tendency to induce gene mutations, low risk of blood poisoning, and limited toxic side effects contribute to the growing interest in the carbazole skeleton [21]. Similarly, drugs that have a carbazole skeleton, such as *Ellipticin* [22, 23], MHY407 [24], and *Rimcazole* [25] (currently only utilized in clinics for neuromodulatory analgesic properties and are not used alone in cancer treatment), and *Ondansetron* [26] (is used to prevent nausea and vomiting caused by chemotherapy, radiation therapy, and surgery for cancer patients), have found widespread use in the treatment of different types of cancer.

On the other hand, the thiosemicarbazide functional group plays a crucial role in synthesizing diverse heterocyclic compounds in synthetic chemistry. It has been reported that thiosemicarbazide derivatives exhibited a wide range of biological activities such as antioxidant, antimicrobial, anti-inflammatory, anticancer, analgesic, anticonvulsant, and antiallergic. Moreover, their complexes with metals have also shown anticancer activity [27, 28]. Certain thiosemicarbazide derivatives revealed antitumor activity in vitro for the non-small cell lung carcinoma cell line HOP-92 and the human melanoma cell line SK-MEL-2 [29, 30]. It was reported that the chalcone-type thiosemicarbazide structure with a p-tolyl substituent on the B phenyl ring is a potent epidermal growth factor receptor (EGFR) kinase inhibitor. In addition, it was reported that this compound exhibited activity against HepG2, a hepatoma cancer cell, suggesting its potential as an anticancer compound [31]. Examining the *N*-(*N*'-phenethylthiocarbamoyl) derivatives showed their activity against human leukemia cells in vitro [32]. Similarly, the thiosemicarbazide derivative containing quinazoline structure revealed antitumor activity as observed previously [33]. 4-(4-(5-mercapto-1,3,4-oxadiazol-2-yl) phenyl) thiosemicarbazide (*Stemazole*) and 4-(2-bromo-4-(5-mercapto-1,3,4-oxadiazole)-2-yl) phenyl) thiosemicarbazide (*Br-Stemazole*) compounds were found to activate the proliferation of stem cells [34]. Moreover, thiosemicarbazide derivative containing benzimidazole ring was effective against HepG2 and PC12 cancer cells, representing hepatocellular carcinoma and neuron cell line, respectively [35].

In our previous studies, some new heterocyclic compounds with different functional groups were synthesized, and their drug similarity properties were investigated theoretically [36]. Furthermore, some carbazole-based acetyl benzohydrazide derivatives were synthesized, and their urease enzyme activities were investigated using Jack bean urease as a model enzyme [37].

In all biological systems, antioxidant defense mechanisms exist to counteract the negative consequences of oxidative stress. Antioxidants are compounds that supply electrons to damaged cells, thereby preventing and stabilizing free radical damage. They also degrade free radicals into waste byproducts that the body rejects [38, 39]. The relationship between antioxidant activity and the PI3K/AKT/mTOR signaling pathway is complicated and multidimensional. By scavenging reactive oxygen species (ROS) and protecting cells from oxidative stress, antioxidants play a pivotal role in maintaining cellular redox balance [40, 41]. Their influence on this signaling pathway involves intricate regulatory mechanisms that warrant further exploration and understanding. The PI3K/AKT/mTOR pathway, on the other hand, regulates biological activities such as cell growth, survival, and metabolism. It’s evident that antioxidants can modulate the PI3K/AKT/mTOR signaling pathway. Based on specific research findings, antioxidants such as resveratrol, curcumin, or vitamin E may impede the process by decreasing PI3K or AKT activity or by blocking mTOR signaling. These benefits are believed to be attributed to their ability to scavenge ROS, alleviate oxidative stress, and indirectly impact pathway regulation [42–44].

Cancer continues to be a prominent contributor to global mortality rates, necessitating urgent efforts to advance the creation of innovative and efficacious therapeutic interventions [45–47]. Annually, over 10 million novel cancers are identified, resulting in significant health complications in both developing and industrialized nations [48]. The development of innovative therapeutic candidates for anticancer treatment remains a significant issue in the field of medicinal chemistry [49]. In addition to the promising roles of carbazole and semithiocarbazide derivatives as anticancer and antioxidant agents, it is worth noting that these moieties have illustrated a critical and potential antimicrobial activity [50]. Their activity against various bacterial strains underscores their potential significance in combating bacterial infections [50–52]. Furthermore, the number of cancer fatalities and new occurrences linked to treatment or persistent infections highlights the interaction between infection and cancer. Infectious agents such as bacteria and viruses are responsible for around 2 million new cancer patients. Patients with chronic infections are more vulnerable to cancer because their immune systems are weakened and unable to fight both the pathogen and the formation of cancer cells. This weakening can also emerge as a result of cancer treatments that are overly harsh on the patient's health, such as chemotherapy, radiation, and surgical resection, making patients vulnerable to infection agents. In addition, chronic infection causes inflammation, which contributes to the development of cancer [53]. The issue of antibiotic resistance has been widely recognized as a significant global public health concern [54], and as a result of the growth of resistant microorganisms and cancer cells, it is necessary to research non-traditional therapeutic options. The earliest and most important method of action may be on cell membranes [55]. Further, anticancer activity generally resembles antibiotic activity, implying a shared mechanism of action [56]. Thus, compounds that target dual action are beneficial for the treatment.

**Fig. 1:**
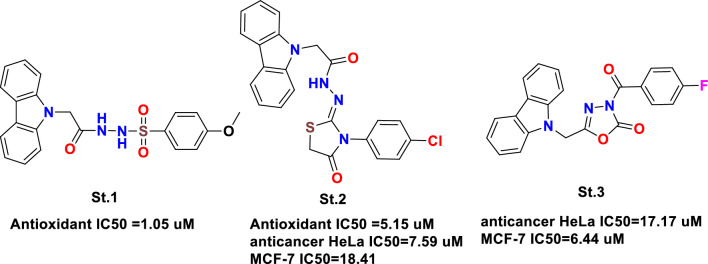
Carbazole derivaiteves with antioxidant and anticancer activities

In our previous preliminary work, a series of carbazole derivatives were synthesized, and their antioxidant and anticancer activities were evaluated; among the synthesized compounds St.1 and St.2 Fig. 1 showed potent antioxidant activities against DPPH with IC_50_ values of 1.05 and 5.15 µM respectively, moreover among this series 4 compounds showed significant anticancer activities on a panel of cancer cell lines and St.2 and St.3 (Fig. 1) showed potent activities on HeLa cancer cell lines with IC_50_ values 7.59 and 17.17 µM and against MCF-7 cancer cell lines with IC_50_ values 18.41 and 6.44 µM respectively [57].

Based on the data obtained, carbazole was chosen as the main skeleton. In contrast, the thiosemicarbazide functional group, known for its biologically active presence in the active ingredient of many drugs, was chosen as the side chain. In this study, new thiosemicarbazide derivatives with carbazole skeleton were synthesized, and subsequently, their antioxidant and anticancer activities were assessed. To investigate the primary source of their antiproliferation activity, the gene expression levels of the compound with the lowest IC_50_ value were determined. Following this, the expression levels of fifty-three genes, including the housekeeping gene, were examined in RNA samples, and a pathway analysis was performed. Furthermore, the docking simulations were performed to elucidate the ligand–protein interaction profile and binding geometry behind the observed anticancer activity. The antibacterial activity was also evaluated for our newly synthesized carbazole and thiosemicarbazide derivatives, and their druggability and pharmacokinetics profiles were assessed.

## Materials and methods

### Chemistry

The chemicals and solvents used were obtained from commercial sources, and the solvents used were of analytical purity. The melting points of the compounds were determined with the SMP50 Automatic Melting Point device, and the values were given without correction. Aluminum plates coated with silica gel 60 F_254_ (Merck) were used to check the purity of the compounds. The different solvent systems were utilized at thin-layer chromatography (TLC), such as hexane/ethyl acetate and dichloromethane/ethyl acetate. Camag UV lamp (254 and 366 nm) was used for monitoring the reactions. The synthesized compounds were purified by the Buchi Pure C-815 Automatic Flash Chromatography System with the UV detector. While Ready-made Buchi EcoFlex and FlashPure silica gel columns (12 g, 24 g, 40 g) were used as stationary phases, hexane/ethyl acetate and dichloromethane/ethyl acetate gradient solvent systems were used as mobile phases. TLC and UPLC/MS-TOF analyses checked the purity of the compounds. The ^1^H-NMR spectra were recorded on Bruker 400-MHz spectrometer and are reported in ppm (δ) relative to tetramethylsilane (TMS) as the internal standard, and ^13^C-NMR (100 MHz) is referenced to DMSO-*d*_6_. Chemical shifts were reported in ppm (parts per million) values. The coupling constants were given as Hertz (Hz). The HRMS spectra of the compounds were obtained from their solutions in methanol with positive ion (ESI+) electrospray ionization techniques using Waters LCT Premier XE UPLC/MSTOF system and MassLynx 4.1 software. Aquity BEH C18 column (2.1 × 100 mm 1.7 μM, flow rate: 0.3 mL/min) was used as stationary phase, and CH_3_CN/H_2_O (1–90%) gradient solvent system containing formic acid (0.1%) as mobile phase. The ChemDraw19 program was used for molecule drawing, and the MestReNova 12 program was used for NMR FID, LC–MS data processing, and spectra analysis.

### General procedure

#### Synthesis of Ethyl 2-(9H-carbazol-9-yl) acetate (2)

To the solution of 9*H*-carbazole (17.9 mmol, 3.0 g) in DMF (25 mL) was added NaH (17.9 mmol, 717.6 mg, 60% dispersion in mineral oil). After it was stirred at room temperature for 2 h, ethyl bromoacetate (17.9 mmol, 1.98 mL) was added to the mixture and cooled to 0 °C in the ice bath. The temperature of the mixture was brought to room temperature and stirred for 24 h. The reaction progress was followed by TLC. After the reaction was completed, the mixture was added dropwise to cold water (200 mL), and the crude product was obtained. The occurred solid was filtrated, dried, and then recrystallized from isopropanol. Yields: 93%, mp: 245–247 °C. Lit: 245–248 °C ^37^. ^1^H NMR (400 MHz, DMSO) δ 8.17 (d, *J* = 3.1 Hz, 2H), 7.56 (dd, *J* = 7.9, 3.1 Hz, 2H), 7.51–7.43 (m, 2H), 7.24 (d, *J* = 3.0 Hz, 2H), 5.35 (s, 2H), 4.23–4.11 (m, 2H), 1.22 (t, *J* = 2.4 Hz, 3H).

#### Synthesis of 2-(9H-carbazol-9-yl) acetohydrazide (3)

To a solution of ethyl 2-(9*H*-carbazol-9-yl) acetate (3.95 mmol, 1.0 g) in ethanol (20 mL) was added hydrazine hydrate (19.74 mmol, 1.24 mL, 80%) and heated with stirring at the boiling temperature of the solvent (12 h). According to TLC data, when ethyl 2-(9*H*-carbazol-9-yl) acetate was finished in the reaction medium, the solvent was removed in vacuo. The crude product was dissolved in methanol (3 mL) and added dropwise to distilled water (100 mL). The resulting solid was filtered and dried under a vacuum. The crude product was purified by recrystallization from isopropanol. Yields: 81%, mp: 239–241 °C. ^1^H NMR (400 MHz, DMSO-*d*_6_) δ 9.54 (s, 1H), 8.20–8.12 (m, 2H), 7.56 (d, *J* = 8.3 Hz, 2H), 7.45 (dd, *J* = 8.3, 7.1) Hz, 2H), 7.22 (td, *J* = 7.6, 1.0 Hz, 2H), 4.99 (s, 2H), 4.33 (d, *J* = 4.3 Hz, 2H). ^13^C NMR (101 MHz, DMSO) δ 167.23, 141.04, 126.09, 122.69, 120.60, 120.55, 119.45, 109.91, 44.67. HRMS (m/z) [M + H]^+^ calcd for C_14_H_14_N_3_O: 240.1137, found: 240.1135.

#### Synthesis of compounds (4a-z)

2-(9*H*-carbazol-9-yl) acetohydrazide (4.18 mmol, 1.0 g,) was dissolved in ethanol (20 mL), and then the requisite isothiocyanate (4.18 mmol) was added in the equivalent molar ratio to this solution. The reactions that took place at the solvent's boiling temperature were completed after 5 h. After removing the solvent in vacuo, the obtained solid was purified using automatic flash chromatography.

*2.2.3.1. 2-(2-(9H-carbazol-9-yl)acetyl)-N-phenylhydrazine-1-carbothioamide ****(4a).*** Purified by flash column chromatography (0%→50% EtOAc in DCM). White solid; isolated yield: 88%, mp 235–237 °C; ^1^H NMR (400 MHz, DMSO-*d*_6_) δ 10.52 (s, 1H), 9.86–9.67 (m, 2H), 8.17 (d, *J* = 7.7 Hz, 2H), 7.61 (d, *J* = 8.1 Hz, 2H), 7.47 (t, *J* = 8.1 Hz, 4H), 7.39 (q, *J* = 7.6 Hz, 2H), 7.24 (tt, *J* = 7.0, 3.3 Hz, 3H), 5.18 (d, *J* = 2.1 Hz, 2H). ^13^C NMR (101 MHz, DMSO) δ 184.16, 167.77, 141.09, 139.50, 128.70, 126.14, 126.02, 122.75, 120.65, 119.58, 109.96, 44.56, 40.64, 40.43, 40.22, 40.01, 39.80, 39.59, 39.39. HRMS (m/z) [M + H]^+^ calcd for C_21_H_19_N_4_OS: 375.1280, found: 375.1245.

*2.2.3.2. 2-(2-(9H-carbazol-9-yl)acetyl)-N-(3-methoxyphenyl)hydrazine-1-carbothioamide ****(4b).*** Purified by flash column chromatography (0%→50% EtOAc in DCM). White solid; isolated yield: 84%, mp 218–220 °C; ^1^H NMR (400 MHz, DMSO-*d*_6_) δ 10.53 (s, 1H), 9.78 (s, 2H), 8.18 (d, *J* = 7.7 Hz, 2H), 7.61 (d, *J* = 8.2 Hz, 2H), 7.51–7.41 (m, 2H), 7.34–7.13 (m, 5H), 7.05 (d, *J* = 8.0 Hz, 1H), 6.80 (d, *J* = 8.3 Hz, 1H), 5.19 (s, 2H), 3.77 (s, 3H). ^13^C NMR (101 MHz, DMSO) δ 171.39, 167.24, 159.57, 141.08, 140.60, 129.46, 126.13, 126.09, 126.01, 122.74, 120.65, 120.63, 119.58, 119.45, 119.42, 109.97, 109.83, 55.61, 44.55. HRMS (m/z) [M + H]^+^ calcd for C_22_H_21_N_4_O_2_S: 405.1385, found: 405.1980.

*2.2.3.3. 2-(2-(9H-carbazol-9-yl)acetyl)-N-(3-morpholinopropyl)hydrazine-1-carbothioamide ****(4c).*** Purified by flash column chromatography (0%→40% EtOAc in Hexane). White solid; isolated yield: 92%, mp 189–191 °C; ^1^H NMR (400 MHz, DMSO-*d*_6_) δ 13.75 (s, 1H), 8.21 (d, *J* = 7.7 Hz, 2H), 7.68 (d, *J* = 8.2 Hz, 2H), 7.55–7.39 (m, 2H), 7.27 (t, *J* = 7.4 Hz, 2H), 5.86 (s, 2H), 3.97–3.77 (m, 2H), 3.47 (t, *J* = 4.6 Hz, 4H), 2.08 (d, *J* = 4.7 Hz, 4H), 1.78 (t, *J* = 6.8 Hz, 2H), 1.21–1.13 (m, 2H). ^13^C NMR (101 MHz, DMSO) δ 167.82, 148.97, 140.44, 123.00, 66.52, 55.01, 53.35, 42.63, 38.71, 24.13. HRMS (m/z) [M + H]^+^ calcd for C_22_H_28_N_5_O_2_S: 426.1964, found: 426.1955.

*2.2.3.4. 2-(2-(9H-carbazol-9-yl)acetyl)-N-(naphthalen-1-yl)hydrazine-1-carbothioamide ****(4d).*** Purified by flash column chromatography (0%→50% EtOAc in Hexane). White solid; isolated yield: 85%, mp 222–224 °C; ^1^H NMR (400 MHz, DMSO-*d*_6_) δ 10.66 (s, 1H), 9.99 (s, 1H), 9.81 (s, 1H), 8.20 (d, *J* = 7.7 Hz, 2H), 7.99 (d, *J* = 8.2 Hz, 1H), 7.91 (dd, *J* = 8.4, 5.5 Hz, 2H), 7.67–7.52 (m, 5H), 7.52–7.40 (m, 4H), 7.25 (t, *J* = 7.4 Hz, 2H), 5.22 (s, 2H). ^13^C NMR (101 MHz, DMSO) δ 171.26, 168.07, 141.14, 134.25, 128.40, 127.49, 126.58, 126.45, 126.13, 126.05, 125.91, 124.05, 122.77, 120.68, 119.59, 119.46, 109.95, 44.51. HRMS (m/z) [M + H]^+^ calcd for C_25_H_21_N_4_OS: 425.1436, found: 425.2122.

*2.2.3.5. 2-(2-(9H-carbazol-9-yl)acetyl)-N-(4-nitrophenyl)hydrazine-1-carbothioamide ****(4e).*** Purified by flash column chromatography (0%→50% EtOAc in DCM). White solid; isolated yield: 94%, mp 235–237 °C; ^1^H NMR (400 MHz, DMSO-*d*_6_) δ 11.01–9.82 (m, 3H), 8.38–8.09 (m, 4H), 7.93 (s, 2H), 7.72–7.53 (m, 2H), 7.46 (t, *J* = 7.5 Hz, 2H), 7.23 (td, *J* = 7.4, 3.3 Hz, 2H), 5.20 (s, 2H). ^13^C NMR (101 MHz, DMSO) δ 171.34, 146.01, 141.19, 141.04, 126.17, 126.03, 125.14, 124.30, 122.74, 120.67, 120.15, 119.61, 119.45, 109.97, 109.88, 44.55. HRMS (m/z) [M + H]^+^ calcd for C_21_H_18_N_5_O_3_S: 420.1130, found: 420.1133.

*2.2.3.6. 2-(2-(9H-carbazol-9-yl)acetyl)-N-(4-methoxyphenyl)hydrazine-1-carbothioamide ****(4f).*** Purified by flash column chromatography (0%→50% EtOAc in DCM). White solid; isolated yield: 95%, mp 210–212 °C; ^1^H NMR (400 MHz, DMSO-*d*_6_) δ 10.50 (s, 1H), 9.66 (d, *J* = 6.1 Hz, 2H), 8.17 (d, *J* = 7.7 Hz, 2H), 7.59 (d, *J* = 8.2 Hz, 2H), 7.46 (ddd, *J* = 8.2, 7.1, 1.3 Hz, 3H), 7.33–7.20 (m, 4H), 6.94 (d, *J* = 8.9 Hz, 2H), 5.17 (s, 2H), 3.77 (s, 3H). ^13^C NMR (101 MHz, DMSO) δ 171.37, 167.72, 157.41, 141.10, 141.07, 132.31, 132.27, 126.13, 126.01, 122.74, 122.71, 120.67, 120.64, 119.56, 119.42, 113.92, 109.95, 109.82, 55.73, 55.70, 44.55, 44.51, 40.65, 40.59, 40.44, 40.38, 40.23, 40.17, 40.02, 39.96, 39.82, 39.75, 39.61, 39.54, 39.40, 39.33. HRMS (m/z) [M + H]^+^ calcd for C_22_H_21_N_4_O_2_S: 405.1385, found: 405.1389.

*2.2.3.7. 2-(2-(9H-carbazol-9-yl)acetyl)-N-(4-bromophenyl)hydrazine-1-carbothioamide ****(4g).*** Purified by flash column chromatography (0%→40% EtOAc in Hexane). White solid; isolated yield: 89%, mp 255–257 °C; ^1^H NMR (400 MHz, DMSO-*d*_6_) δ 10.47 (s, 1H), 9.75 (d, *J* = 11.0 Hz, 2H), 8.16 (d, *J* = 7.7 Hz, 2H), 7.58 (dd, *J* = 19.9, 8.3 Hz, 4H), 7.52–7.37 (m, 4H), 7.24 (t, *J* = 7.4 Hz, 2H), 5.17 (s, 2H). ^13^C NMR (101 MHz, DMSO) δ 171.35, 167.75, 141.08, 138.96, 131.54, 126.14, 126.01, 122.75, 120.64, 120.61, 119.58, 109.95, 44.57. HRMS (m/z) [M + H]^+^ calcd for C_21_H_18_BrN_4_OS: 453.0385, found: 453.1194.

*2.2.3.8. 2-(2-(9H-carbazol-9-yl)acetyl)-N-(4-chlorophenyl)hydrazine-1-carbothioamide ****(4h).*** Purified by flash column chromatography (0%→40% EtOAc in Hexane). White solid; isolated yield: 90%, mp 235–237 °C; ^1^H NMR (400 MHz, DMSO-*d*_6_) δ 10.49 (s, 1H), 9.75 (d, *J* = 28.4 Hz, 2H), 8.16 (d, *J* = 7.7 Hz, 2H), 7.60 (d, *J* = 8.2 Hz, 2H), 7.53 (d, *J* = 8.8 Hz, 2H), 7.50–7.39 (m, 4H), 7.23 (t, *J* = 7.4 Hz, 2H), 5.17 (s, 2H). ^13^C NMR (101 MHz, DMSO) δ 171.38, 167.88, 141.20, 141.06, 138.50, 128.62, 126.14, 126.02, 122.73, 120.66, 120.62, 119.59, 119.44, 109.95, 109.83, 44.54. HRMS (m/z) [M + H]^+^ calcd for C_21_H_18_ClN_4_OS: 409.0890, found: 409.1523.

*2.2.3.9. 2-(2-(9H-carbazol-9-yl)acetyl)-N-(4-(trifluoromethyl)phenyl)hydrazine-1-carbothioamide ****(4k).*** Purified by flash column chromatography (0%→40% EtOAc in Hexane). White solid; isolated yield: 92%, mp 177–179 °C; ^1^H NMR (400 MHz, DMSO-*d*_6_) δ 8.17 (d, *J* = 7.7 Hz, 2H), 7.81 (d, *J* = 8.5 Hz, 2H), 7.73 (d, *J* = 8.4 Hz, 2H), 7.62 (d, *J* = 8.2 Hz, 2H), 7.47 (ddd, *J* = 8.3, 7.0, 1.2 Hz, 2H), 7.24 (t, *J* = 7.4 Hz, 2H), 5.19 (s, 2H). ^13^C NMR (101 MHz, DMSO) δ 171.38, 167.88, 143.35, 141.21, 141.07, 128.84, 126.16, 126.03, 125.83, 123.45, 122.75, 120.66, 119.62, 119.60, 109.96, 44.56. HRMS (m/z) [M + H]^+^ calcd for C_22_H_18_F_3_N_4_OS: 443.1153, found: 443.1693.

*2.2.3.10. 2-(2-(9H-carbazol-9-yl)acetyl)-N-cyclohexylhydrazine-1-carbothioamide ****(4m).*** Purified by flash column chromatography (0%→50% EtOAc in DCM). White solid; isolated yield: 78%, mp 211–213 °C; ^1^H NMR (400 MHz, DMSO-*d*_6_) δ 10.19 (s, 1H), 9.32 (d, *J* = 107.9 Hz, 1H), 8.16 (d, *J* = 7.7 Hz, 2H), 7.59 (d, *J* = 8.3 Hz, 2H), 7.46 (q, *J* = 7.8, 6.2 Hz, 3H), 7.23 (t, *J* = 7.4 Hz, 2H), 5.13 (s, 2H), 2.03–1.50 (m, 6H), 1.25 (d, *J* = 10.3 Hz, 5H). ^13^C NMR (101 MHz, DMSO) δ 171.47, 167.47, 141.15, 141.04, 126.12, 126.02, 122.73, 122.71, 120.64, 119.57, 119.55, 109.95, 53.26, 44.47, 32.40, 25.65, 25.26. HRMS (m/z) [M + H]^+^ calcd for C_21_H_25_N_4_OS: 381.1749, found: 381.2469.

*2.2.3.11. 2-(2-(9H-carbazol-9-yl)acetyl)-N-(p-tolyl)hydrazine-1-carbothioamide ****(4n).*** Purified by flash column chromatography (0%→40% EtOAc in Hexane). White solid; isolated yield: 81%, mp 180–182 °C; ^1^H NMR (400 MHz, DMSO-*d*_6_) δ 10.45 (s, 1H), 9.61 (d, *J* = 10.4 Hz, 2H), 8.16 (d, *J* = 7.7 Hz, 2H), 7.60 (d, *J* = 8.2 Hz, 2H), 7.46 (t, *J* = 7.5 Hz, 2H), 7.34 (d, *J* = 8.0 Hz, 2H), 7.30–7.13 (m, 4H), 5.17 (s, 2H), 2.31 (s, 3H). ^13^C NMR (101 MHz, DMSO) δ 171.38, 167.75, 141.22, 141.08, 136.89, 135.10, 129.16, 126.13, 126.02, 122.73, 120.66, 120.62, 119.57, 119.43, 109.95, 109.82, 44.53, 21.05. HRMS (m/z) [M + H]^+^ calcd for C_22_H_21_N_4_OS: 389.1436, found: 389.2215.

*2.2.3.12. N-(2-(2-(9H-carbazol-9-yl)acetyl)hydrazine-1-carbonothioyl)benzamide ****(4o).*** Purified by flash column chromatography (0%→50% EtOAc in DCM). White solid; isolated yield: 75%, mp 189–191 °C; ^1^H NMR (400 MHz, DMSO-*d*_6_) δ 12.70 (s, 1H), 11.57 (d, *J* = 108.8 Hz, 2H), 8.17 (d, *J* = 7.7 Hz, 2H), 7.95 (d, *J* = 7.7 Hz, 2H), 7.65 (d, *J* = 8.1 Hz, 3H), 7.49 (dt, *J* = 16.4, 7.8 Hz, 4H), 7.24 (t, *J* = 7.6 Hz, 2H), 5.31 (s, 2H). ^13^C NMR (101 MHz, DMSO) δ 177.70, 168.51, 165.48, 141.03, 133.64, 132.24, 129.33, 129.20, 128.91, 128.78, 126.22, 122.74, 120.67, 119.66, 119.63, 109.99, 44.21. HRMS (m/z) [M + H]^+^ calcd for C_22_H_19_N_4_O_2_S: 403.1229, found: 403.2189.

*2.2.3.13. 2-(2-(9H-carbazol-9-yl)acetyl)-N-benzylhydrazine-1-carbothioamide ****(4p).*** Purified by flash column chromatography (0%→40% EtOAc in Hexane). White solid; isolated yield: 86%, mp 169–171 °C; ^1^H NMR (400 MHz, DMSO-*d*_6_) δ 10.32 (s, 1H), 9.43 (s, 1H), 8.16 (d, *J* = 7.7 Hz, 2H), 7.55 (d, *J* = 8.2 Hz, 2H), 7.51–7.37 (m, 3H), 7.32 (d, *J* = 4.3 Hz, 4H), 7.29–7.19 (m, 3H), 5.12 (s, 2H), 4.81 (d, *J* = 6.0 Hz, 2H). ^13^C NMR (101 MHz, DMSO) δ 171.29, 167.78, 141.09, 141.05, 139.62, 128.70, 128.60, 128.07, 127.36, 127.15, 126.11, 126.02, 122.71, 120.65, 120.62, 119.56, 119.46, 109.90, 109.67, 47.20, 44.43. HRMS (m/z) [M + H]^+^ calcd for C_22_H_21_N_4_OS: 389.1463, found: 389.2268.

*2.2.3.14. 2-(2-(9H-carbazol-9-yl)acetyl)-N-(bicyclo[2.2.1]heptan-2-yl)hydrazine-1-carbothioamide ****(4r).*** Purified by flash column chromatography (0%→40% EtOAc in Hexane). White solid; isolated yield: 73%, mp 199–201 °C; ^1^H NMR (400 MHz, DMSO-*d*_6_) δ 10.20 (s, 1H), 9.31 (d, *J* = 88.8 Hz, 1H), 8.16 (d, *J* = 7.8 Hz, 2H), 7.60 (d, *J* = 8.2 Hz, 2H), 7.49–7.42 (m, 2H), 7.27–7.20 (m, 2H), 5.14 (s, 2H), 3.93 (s, 1H), 2.28–2.15 (m, 2H), 1.70–1.61 (m, 1H), 1.54–1.37 (m, 3H), 1.32 (d, *J* = 10.5 Hz, 2H), 1.25–1.04 (m, 4H). ^13^C NMR (101 MHz, DMSO) δ 171.44, 167.46, 141.18, 141.02, 126.13, 126.00, 122.75, 122.73, 122.71, 120.66, 120.62, 119.58, 119.42, 109.91, 109.77, 57.69, 44.47, 42.19, 35.75, 35.41, 28.37, 26.50. HRMS (m/z) [M + H]^+^ calcd for C_22_H_25_N_4_OS: 393.1749, found: 393.2584.

*2.2.3.15. 2-(2-(9H-carbazol-9-yl)acetyl)-N-phenethylhydrazine-1-carbothioamide ****(4s).*** Purified by flash column chromatography (0%→40% EtOAc in Hexane). White solid; isolated yield: 84%, mp 170–172 °C; ^1^H NMR (400 MHz, DMSO-*d*_6_) δ 10.26 (s, 1H), 9.45 (d, *J* = 83.8 Hz, 1H), 8.16 (d, *J* = 7.7 Hz, 2H), 8.08 (s, 1H), 7.57 (d, *J* = 8.2 Hz, 2H), 7.52–7.40 (m, 3H), 7.38–7.15 (m, 7H), 5.11 (s, 2H), 3.86–3.57 (m, 2H), 2.86 (dd, *J* = 9.1, 6.4 Hz, 2H). ^13^C NMR (101 MHz, DMSO) δ 167.73, 159.32, 141.05, 139.65, 129.10, 128.91, 126.65, 126.13, 122.71, 120.66, 119.57, 109.91, 45.78, 44.44, 35.37. HRMS (m/z) [M + H]^+^ calcd for C_23_H_23_N_4_OS: 403.1593, found: 403.2480.

*2.2.3.16. 2-(2-(9H-carbazol-9-yl)acetyl)-N-isopropylhydrazine-1-carbothioamide ****(4t).*** Purified by flash column chromatography (0%→50% EtOAc in DCM). White solid; isolated yield: 90%, mp 186–188 °C; ^1^H NMR (400 MHz, DMSO-*d*_6_) δ 10.18 (s, 1H), 9.32 (d, *J* = 117.8 Hz, 1H), 8.16 (d, *J* = 7.7 Hz, 2H), 7.65–7.39 (m, 5H), 7.23 (t, *J* = 7.4 Hz, 2H), 5.13 (s, 2H), 4.55–4.21 (m, 1H), 1.15 (d, *J* = 6.5 Hz, 6H). ^13^C NMR (101 MHz, DMSO) δ 171.44, 167.51, 141.14, 141.05, 126.12, 126.02, 122.72, 120.64, 119.56, 119.50, 109.95, 109.93, 109.75, 46.22, 44.46, 44.41, 22.44, 22.40. HRMS (m/z) [M + H]^+^ calcd for C_18_H_21_N_4_OS: 341.1436, found: 341.2164.

*2.2.3.17. 2-(2-(9H-carbazol-9-yl)acetyl)-N-allylhydrazine-1-carbothioamide ****(4y).*** Purified by flash column chromatography (0%→50% EtOAc in DCM). White solid; isolated yield: 78%, mp 205–207 °C; ^1^H NMR (400 MHz, DMSO-*d*_6_) δ 10.31 (s, 1H), 9.52 (d, *J* = 93.9 Hz, 1H), 8.20–8.11 (m, 2H), 7.64–7.47 (m, 2H), 7.44 (ddd, *J* = 8.3, 7.1, 1.3 Hz, 2H), 7.30–7.14 (m, 2H), 5.86 (ddt, *J* = 17.3, 10.2, 5.0 Hz, 1H), 5.25–4.98 (m, 4H), 4.17 (d, *J* = 5.6 Hz, 2H). ^13^C NMR (101 MHz, DMSO) δ 167.73, 141.14, 141.06, 135.31, 126.11, 126.02, 122.71, 120.64, 119.55, 119.45, 116.25, 115.75, 109.92, 109.76, 46.34, 44.44. HRMS (m/z) [M + H]^+^ calcd for C_18_H_19_N_4_OS: 339.1280, found: 339.2032.

*2.2.3.18. 2-(2-(9H-carbazol-9-yl)acetyl)-N-methylhydrazine-1-carbothioamide ****(4z).*** Purified by flash column chromatography (0%→50% EtOAc in DCM). White solid; isolated yield: 84%, mp 173–175 °C; ^1^H NMR (400 MHz, DMSO-*d*_6_) δ 10.22 (s, 1H), 9.41 (d, *J* = 104.2 Hz, 1H), 8.16 (d, *J* = 7.7 Hz, 2H), 8.01 (d, *J* = 5.6 Hz, 1H), 7.56 (d, *J* = 8.2 Hz, 2H), 7.46 (t, *J* = 7.5 Hz, 3H), 7.23 (dd, *J* = 8.8, 6.2 Hz, 2H), 5.11 (s, 2H), 2.95 (d, *J* = 4.4 Hz, 3H). ^13^C NMR (101 MHz, DMSO) δ 171.43, 167.79, 141.04, 126.14, 126.03, 122.68, 120.67, 120.62, 119.56, 119.44, 109.88, 109.76, 44.39, 31.42. HRMS (m/z) [M + H]^+^ calcd for C_16_H_17_N_4_OS: 313.1123, found: 313.1103.

### Biological methods

#### Antioxidant DPPH assay

To estimate the Carbazole derivatives' antioxidant potential, a solution of each compound (1 mM/mL) in methanol was serially diluted with methanol to obtain a concentration of 1, 2, 5, 10, 20, 50, and 100 µM/mL. Then, DPPH (2,2-diphenyl-1-picrylhydrazyl) reagent (Sigma, USA) was dissolved in 0.002% w/v methanol and mixed with the previously prepared working concentrations in a 1:1 ratio. The same procedures were repeated for Trolox (Sigma-Aldrich, Denmark), which was used as a positive control. All of the solutions were kept in the dark chamber for 30 min at an ordinary temperature. Then, their absorbance values were measured at a wavelength of 517 nm utilizing a UV–visible spectrophotometer. The DPPH inhibition potentials by Carbazole derivatives and Trolox were determined employing the following equation:

$$\text{DPPH inhibition }(\text{\%})=\frac{\text{abs}(\text{blank})-\text{ abs}(\text{sample})}{\text{abs}(\text{blank})}*100\text{\%}$$

where abs_blank_ is the blank absorbance and abs_sample_ is the absorbance of the samples [58]. The antioxidant half-maximal inhibitory concentration (IC_50_) of the synthesized carbazole derivatives and Trolox were assessed by using an online tool “Quest Graph™ IC50 Calculator.” AAT Bioquest, Inc., 25 May. 2023, https://www.aatbio.com/tools/ic50-calculator. 

#### In vitro* antimicrobial activity*

Using Muller Hinton agar medium, the antibacterial activity was evaluated by using 24 h cultures of *Escherichia coli, Staphylococcus aureus, and Candida albicans*. *E. coli* and *S. aureus* were incubated in 1% Mueller Hinton Broth (MHB) liquid medium at 37 °C for 24 h; *C. albicans* was incubated in Sabouraud Dextrose Broth (SDB) at 30 °C for 48 h. MIC (Minimum Inhibitory Concentration) and MBC (Minimum Bactericidal Concentration) tests were performed to determine antimicrobial activity. This stage was carried out in a sterile cabinet.

The density of microorganisms in fresh cultures was adjusted according to the MacFarland 0.5 scale. For that, the microorganisms were centrifuged at 4000 rpm for 20 min. The supernatant was poured off, and the pellet was washed with 1 mL of 0.9% NaCl solution (physiological saline). The centrifugation was then repeated. The pellet was dissolved again with saline solution and adjusted to 0.5 MacFarland. In the experiment performed in a sterile 96-well plate, compound **4o** was diluted ½ with DMSO to prepare a stock solution. In the first, 100 µl of medium and 100 µl of compound **4o** as initial dilutions. Dilution was performed seven times at a ratio of ½. Then, 5 µl of fresh microorganism cultures prepared as MacFarland 0.5 were inoculated. The control group was prepared as 100 µl medium, 100 µl distilled water, and 5 µl microorganisms. The plates were incubated at 37 °C and 30 °C for 24 h.

After incubation, 10 µl of the sample was taken, dropped onto the respective media, and incubated for 24 h at appropriate temperatures to clearly distinguish the residues left by the compound **4o** and the growth of microorganisms. Mueller Hinton Agar (MHA) was used for *E. coli* and *S. aureus,* and Potato Dextrose Agar (PDA) was used for *C. albicans*. After incubation, MIC and MBC values were determined. The first well with a significant decrease in growth was considered as MIC, and the first well with no growth was considered as MBC [59, 60].

#### Cell line and reagents

MCF-7 cancer cell lines were cultured at Dulbecco’s Modified Eagle Medium High Glucose (DMEM) (EuroClone, Via Figino, Italy); fetal bovine serum (FBS) and MTT (3-(4,5-Dimethylthiazol-2-yl)-2,5-Diphenyl Tetrazolium Bromide) were provided by Sigma-Aldrich (Darmstadt, Germany); ApopNexin Annexin-V-FITC with PI Apoptosis Kit was obtained from Merck (Darmstadt, Germany); Total RNA Isolation Kit was provided by Analytic Jena (Jena, Germany), SensiFast cDNA Synthesis Kit was provided by Meridian Bioscience (Cincinati, USA), SybrGreen Master Mix was obtained from EuroClone (Via Figino, Italy) [61, 62].

#### *Cancer* pathway array

RNA extracted from MCF-7 cells treated with 4o converted to cDNA. The expression level of the cell line was compared to non-treated cells to evaluate the effect of the compound with previously established methods [61, 62]. The results were analyzed using gene enrichment analysis (Reactome and EnrichR pathways). To confirm PI3K/AKT/mTOR pathway expression of PIK3CA, PIK3CB, PTEN, AKT1, and mTOR gene expression levels measured. In the absence of 4o, the expression level of each gene is taken as a unity by using b-actin housekeeping gene expression. Gene expression in the presence of the compound was taken relative to unity (See supplementary file for primers and fold expression change) [61–63].

#### Apoptosis

FITC Annexin-V apoptosis detection kit with PI, ApopNexinTM FITC (APT750, Merk, Germany) was used for apoptosis assay. MCF-7 cells were seeded into a 6-well plate (1 × 10^6^ cells per well). Then, the cells were incubated with the compounds at the cytotoxic concentration by employing a cell viability assay. Apoptotic cells were cultured with the compounds, the medium was removed after incubation, and the cells were washed twice with 5 mL of pre-chilled phosphate-buffered saline. Cells washed with PBS were lifted with Trypsin–EDTA and centrifuged at 400 rpm for 5 min. The resulting cells were each treated with 1 mL of cold 1-X Binding Buffer (Sigma Aldrich, USA). Then, 200 μL of the mixture was taken and placed in a flow cytometry tube, and the other part containing cells was kept on ice. First, 3 µl of ApopNexinTM FITC and 2 µl of 100X propidium iodide (PI) (P4170, Sigma Aldrich, USA) were added to the solution taken into the sample tube and mixed. The resulting mixture was incubated for 15 min in the dark at room temperature. At the end of the incubation, the samples were measured in flow cytometry (Beckman Coulter Cytoflex) and data were obtained [64].

#### Cell cycle

The cells were cultured (3 × 10^5^/well) at the IC_50_ values. The manufacturer protocol (Sigma-Aldrich Mak344 Cell Cycle Analysis Kit) was used to perform cell cycle experiments and analyzed by flow cytometry (Beckman Coulter Cytoflex).

### Computational studies

In silico computational studies are pivotal in modern drug discovery and development processes. These computational techniques, such as molecular docking and drug-likeness analysis, enable researchers to simulate and analyze molecular interactions between potential drug candidates and their biological targets [65]. By harnessing the power of computational algorithms and predictive models, scientists can expedite the identification of promising drug candidates, optimize their chemical structures for enhanced efficacy and safety, and prioritize compounds for further experimental validation [66]. Additionally, in silico studies provide valuable insights into the physicochemical properties, pharmacokinetics, and potential biological activities of drug candidates, thereby aiding in the rational design and optimization of novel therapeutics. Overall, integrating in silico computational approaches alongside experimental methodologies has revolutionized the drug discovery process, facilitating the development of safer, more efficacious, and targeted therapies for various diseases [67–70]. The Maestro Schrodinger 2021-3 platform was used for the in silico molecular docking calculations. The molecular docking study followed the ligand preparation process, subsequent protein preparation, and receptor grid generation.

#### Ligand preparation

The ligand preparation step implicates several key steps, such as adjusting the bond angles and lengths, adding hydrogen atoms, and generating their ionization states at the target pH (7.4 ± 1.0). Also, it aims to investigate the most favorable spatial and 3D conformational arrangements of the ligand, the possible low-energy conformations, tautomers, stereochemistries, and corrected chiralities generated. Subsequently, the resolved structures were minimized at the OPLS4 force field and saved to apply for docking simulations [71].

##### Protein preparation

The Protein Preparation Wizard module, integrated into the Maestro Schrödinger interface, was used to resolve the selected protein targets. First, the crystallographic structure for each utilized protein was obtained from Protein Data Bank-RCSB (PDB; http://www.rcsb.org/pdb). The preparation step encompassed various tasks, including assigning bond orders, adding hydrogen atoms, filling in missing side chains and loops using Prime, generating het states at pH 7 ± 1 using Epik, optimizing the protein structure, removing water molecules within a 3 Å range beyond hets, and finally minimizing the structure using OPLS4 force [72, 73].

##### Receptor grid generation

A receptor grid box was generated to identify the specific region of interest within the target protein to enhance docking accuracy. For the selected protein structures, the grid box dimensions were set automatically according to the native ligand and centered at the centroid of selected residues within the binding site. At the same time, other parameters were constrained as default [74]. In the last part, the SwissADME online platform [75] was applied to obtain drug-likeness and a few ADME traits.

##### Statistical analysis

All of the obtained results were expressed as mean ± SD standard deviation; the result was considered significant when the p-value was < 0.05. The unpaired t-test was used to analyze the data.

## Results and discussion

### Chemistry

**Scheme 1:**
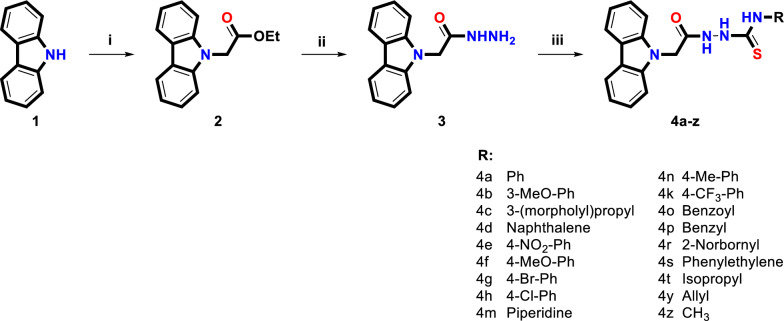
Reagents and conditions: (i) ethyl bromoacetate, NaH, DMF, rt.; (ii) NH_2_NH_2_.H_2_O, ethanol, refluxed, 12 h.; (iii) isothiocyanate derivatives, ethanol, refluxed, 5h

This study involved synthesizing and characterizing new carbazole compounds carrying a thiosemicarbazide functional group, investigating their anticancer activity, and gene expression levels of bioactive compounds. Synthetic routes for preparing compounds are summarized in Scheme 1**,** and NMR spectra of all the compounds are given in the Supplementary Information section.

In the first step of the synthesis studies, Ethyl-2-(9*H*-carbazol-9-yl) acetate **(2)** containing the ester functional group on the nitrogen atom was synthesized from the reaction of commercially purchased 9*H*-carbazole with ethyl bromoacetate. The hydrazide derivative 2-(9*H*-carbazol-9-yl) acetohydrazide (**3**) was obtained from the reaction of compound-**2** with hydrazine hydrate. New thiosemicarbazide derivatives **(4a-z)**, also known as hydrazine-1-carbothioamide, were synthesized in high yield from the reaction of compound-**3** with aliphatic, alicyclic, bicyclic, and aromatic substituted different isothiocyanates.

All synthesized compounds were purified by automatic flash chromatography with UV detector, and the structure analysis was performed by taking ^1^H-NMR and ^13^C_APT_-NMR spectra. Mass spectra were obtained by high-resolution mass spectrometry. The structure–activity evaluation was carried out with eighteen different compound derivatives that were synthesized and characterized. The chemical structures of the compounds were found to be compatible with the targeted molecular structures.

### Antioxidant results

Antioxidant compounds' radical scavenging effect is mediated by a well-known process in which reactive free radicals interact with antioxidants by abstracting a hydrogen atom. This process aids in the neutralization of free radicals and the reduction of oxidative stress. The SAR results for the produced compounds were analyzed based on their IC_50_ values against DPPH, as shown in Table 1. The SAR analysis demonstrates that substances containing particular substituents, such as methoxy (**4b**), chloro (**4h**), p-tolyl (**4n**), allyl (**4y**), and certain alkyl/cycloalkyl groups (**4k**, **4m**), have higher antioxidant activity. These results shed light on the structural requirements for increased antioxidant capability in this class of carbazole derivatives. The thiosemicarbazide functional group is present in compounds **4a**, **4b**, **4c**, and **4d**. When their IC_50_ values are compared, we can observe that compound **4b** (5.15 µM) has the highest antioxidant activity among these compounds. This shows that adding a methoxy group to the phenyl ring (as in compound **4b** and **St.1 **Fig. 1) improves antioxidant effectiveness compared to other compounds with other substituents. The phenyl ring substituents in compounds **4f** (3.41 µM), **4g** (4.14 µM), and **4h** (0.73 µM) vary. Notably, compound **4h** with a chloro group displayed the highest antioxidant activity among them, implying that the chloro group’s electron-withdrawing nature may contribute to higher antioxidant capacity and this could be similar impact of **St.2** (Fig. 1). Compound **4n** (1.52 µM), which has a p-tolyl substituent, demonstrated relatively high antioxidant activity when compared to other compounds in the series, indicating that this particular substituent affects antioxidant potency.

**Table 1:** The IC_50_ values (µM) of the synthesized compounds against DPPH radical scavenging method, and MCF-7 cell line


Code	R	IC_50_ values (µM)	
		DPPH Radical Scavenging	Carbazole Derivatives Effect on MCF-7 Cell Line
Positivecontrol	–	7.71 ± 1.47^a^	5.06 ± 0.21^b^
**4a**	Ph	52.06 ± 2.42	NI
**4b**	3-MeO-Ph	5.15 ± 1.02	NI
**4c**	3-(morpholyl)propyl	8.99 ± 2.17	NI
**4d**	Naphthalene	29.71 ± 1.77	NI
**4e**	4-NO_2_-Ph	50.82 ± 1.88	NI
**4f**	4-MeO-Ph	3.41 ± 1.05	NI
**4g**	4-Br-Ph	4.14 ± 0.75	NI
**4h**	4-Cl-Ph	**0.73 ± 0.54**	NI
**4m**	Piperidine	2.37 ± 1.70	NI
**4n**	4-Me-Ph	1.52 ± 0.58	NI
**4k**	4-CF_3_-Ph	2.08 ± 0.72	NI
**4o**	Benzoyl	157.94 ± 2.54	**2.02 ± 0.04 µM**
**4p**	Benzyl	10.15 ± 1.85	NI
**4r**	2-Norbornyl	19.94 ± 1.55	**4.99 ± 0.42 µM**
**4s**	Phenylethylene	3.05 ± 1.75	NI
**4t**	Isopropyl	15.88 ± 2.07	NI
**4y**	Allyl	**0.38 ± 0.05**	NI
**4z**	CH_3_	21.58 ± 0.87	NI

Positive controls: ^a^ Trolox, ^b^Doxorubicin (*p* < *0.05)*, IC_50_ values of the compounds that are higher than 20 µM considered as no inhibition (NI)

The antioxidant results of the Carbazole compounds against DPPH show varied degrees of antioxidant activity. Some chemicals have an antioxidant capacity equivalent to or greater than the positive control Trolox IC_50_ value of 7.71 µM, whereas others have lower antioxidant activity. These findings show the compounds' varied spectrum of antioxidant capacities and underscore the significance of more research and evaluation to grasp their potential in contrast to other well-known antioxidants.

### Cell cytotoxicity experiments

**Fig. 2:**
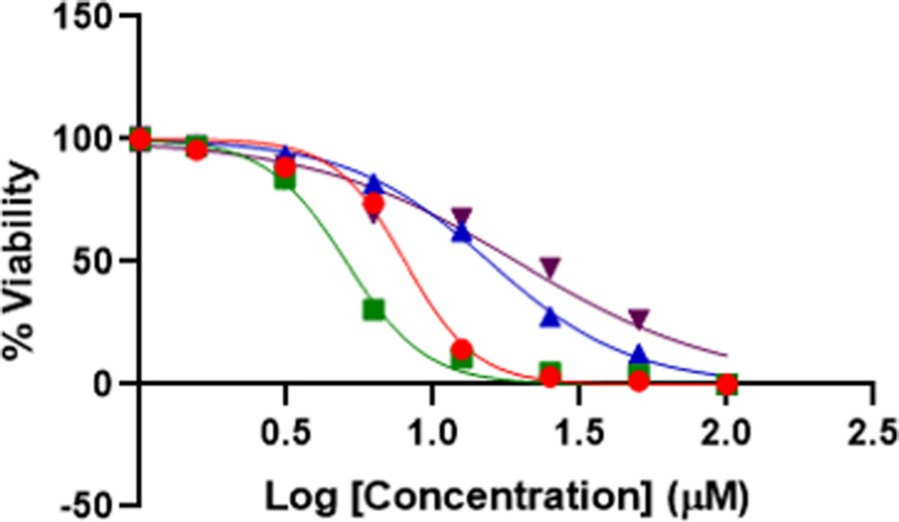
MCF-7 cells treated with '4o' are represented by red circles, while MCF-7 cells treated with doxorubicin are depicted as green rectangles. Non-cancerous L929 cells treated with '4o' are shown as blue upside triangles, and those treated with doxorubicin are displayed as brown downside triangles

Cells (MCF-7 and L929) were incubated in 75 cm^2^ sterile flasks with 10% FBS, 100 U/mL penicillin, and 100 µg/mL streptomycin. The cells were grown at 37 ^○^C in 5% CO_2,_ and MTT was used to screen the viability of the cells. Cells were seeded at a density of 10^4^ cells per well. The cells were then cultured for 24 h in 100 µl of DMEM complete medium. After pretreatment with different concentrations of the compounds for 48 h, 10 µl of 5 mg/mL MTT solution was added to each well and incubated for 4 h at 37 ^○^C, and 100 µl of DMSO was used in each well to dissolve the blue formazan crystals. Then, the absorbance was measured at 570 nm [61]. The cell viability percentages of compound 4o against MCF-7 cancer cell lines and L929 normal cell lines were presented in Fig. 2 in comparison with doxorubicin anticancer agent.

Within the cell cytotoxicity test, the effect of compound **4o** on MCF-7 cell lines was compared to the clinical drug doxorubicin. The IC_50_ value for compound **4o** on MCF-7 cell lines is 2.02 µM, while the value for L929 is 37.78 µM, indicating a selective index (SI) value equal to 18.70 for this compound. The IC_50_ value for doxorubicin is 5.06 and 19.33 µM on MCF-7 and L929 cell lines, respectively, and calculates the SI value as 3.82. These values indicate that compound **4o** has higher potential to become an efficient anticancer drug. The effect of the compound on cancer pathways was determined through the array studies.

### Array and gene enrichment analysis

An array consists of 92 genes, expression enhancements analyzed against two housekeeping genes (GAPDH and ACTINB). The normalized effect in the presence and absence of the compound were then compared. The enrichment analysis indicated that 4o drives MCF-7 cells to apoptosis and arrest cells at the G2-M checkpoint (Table 2).

**Table 2:** Gene enrichment analysis of MCF-7 cells against **4o** compound

Term	p-value	q-value	Overlap genes
Apoptosis	3.185742e-11	1.465441e-09	[CASP7, BCL2L11, CCND2, DDIT3, HSPB1, CASP2, XIAP, HMOX1, FASLG, SOD1, BIRC3]
mTORC1 signaling	5.563581e-09	1.279624e-07	[PPP1R15A, MAP2K3, ACLY, G6PD, LDHA, PFKL, IGFBP5, DDIT3, MCM2, AURKA]
Hypoxia	1.119546e-06	1.029982e-05	[PPP1R15A, LDHA, PFKL, IGFBP3, DDIT3, HMOX1, ADM, PGF]
G2-M checkpoint	1.119546e-06	1.029982e-05	[CDC20, DKC1, CDK4, STMN1, MAPK14, MKI67, MCM2, AURKA]
Glycolysis	1.119546e-06	1.029982e-05	[G6PD, LDHA, IGFBP3, STMN1, NOL3, GUSB, AURKA, SOD1]
Adipogenesis	1.304867e-05	8.574841e-05	[ACYL, PFKL, ANGPT1, CPT2, GPD2, LPL, SOD1]
p53 pathway	1.304867e-05	8.574841e-05	[PPP1R15A, CCND3, CCND2, APAF1, DDIT3, HMOX1, ERCC5]
Myc targets V1	1.309957e-04	5.478004e-04	[CDC20, LDHA, HSP90AB1, CDK4, COX5A, MCM2]
Epithelial mesenchymal transition	1.309957e-04	5.478004e-04	[FOXC2, CDH2, IGFBP3, VEGFC, SNAI2, FGF2]
TNF-alpha signaling via NF-kB	1.309957e-04	5.478004e-04	[PPP1R15A, MAP2K3, SERPINB2, CCL2, ETS2, BIRC3]

Table 2 shows that the **4o** compound drives MCF-7 cells to apoptosis, and the compound directly affects proliferation and cell growth through the metabolic genes. The compound also affects metabolism through mTORC1, glycolysis, and adipogenesis. Compound **4o** does not only induce apoptosis but also enhances the cell cycle through G2-M checkpoint arrest. Therefore, cell cycle and apoptosis experiments were performed on flow cytometry to support gene enrichment analysis.

### Apoptosis

**Fig. 3:**
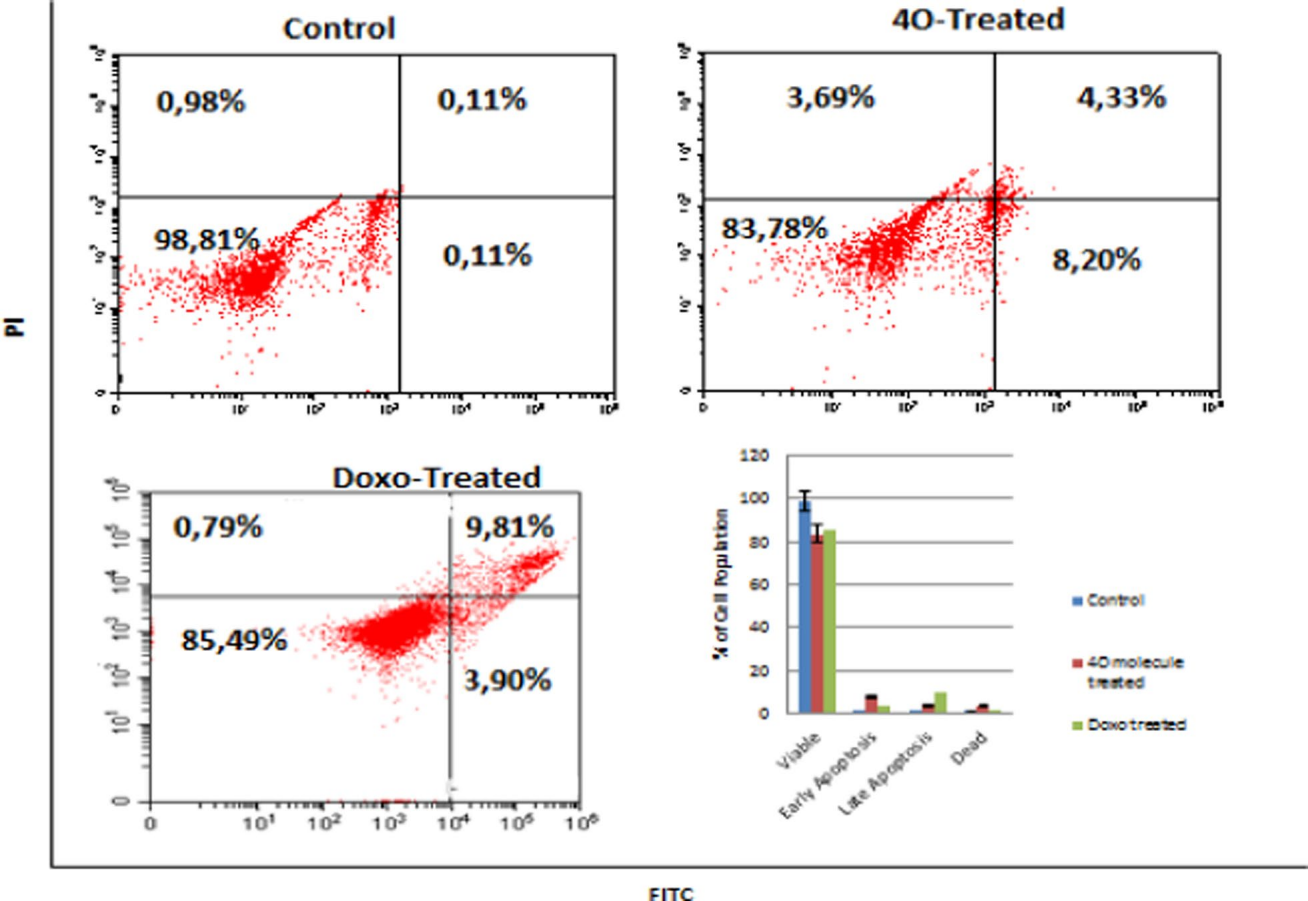
Effect of **4o** on apoptotic cell population of MCF-7 cell line, left untreated, right **4o** treated

As suggested by the results of the array experiments, flow cytometry experiments were performed to support the induction of apoptosis. The compound **4o** drives cancer cells to apoptosis, and the results are further compared to doxorubicin-treated MCF-7 cells. In the absence of the compound **4o**, cell populations are found to be at the pre-apoptotic (0.11%), post-apoptotic (0.11%), necrosis (0.98%), and viable (98.81%) (Fig. 3), while the ratio substantially alters in the presence of **4o** as pre-apoptotic (8.20%), post apoptotic (4.33%), necrosis (3.69%), and viable (83.78%) (Fig. 3), this gives a total of 16.22% dead cells while the ratio with the clinical drug doxorubicin is 14.50%. Thus, the compound is a promising drug candidate.

Figure 3 shows that **4o** drives MCF-7 cells to apoptosis, which is supported by gene enrichment analysis. The comparison of the compound with doxorubicin suggests it’s potential as anticancer drug. Table 2 indicates the genes involved in the apoptotic cell death pathway. The analysis indicates that the compound arrests the cells at the G2/M point. Further, gene enrichment analysis indicates that the compound blocks PI3K/AKT/mTOR Signaling.

### Cell cycle

**Fig. 4:**
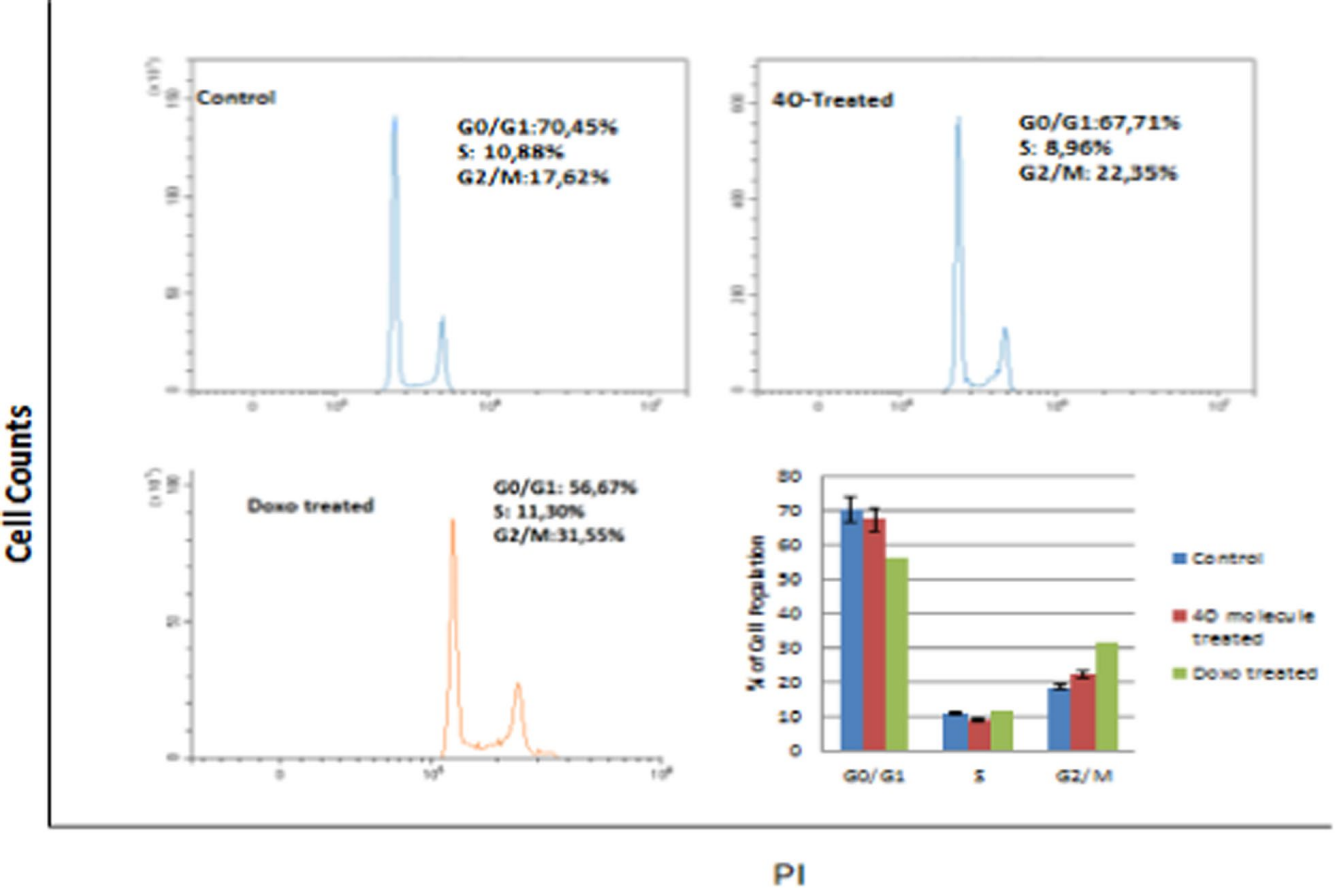
Evaluation of cell cycle in MCF-7 cells treated with **4o** molecule (12 μM) after 48 h

As depicted in Fig. 4, molecule **4o** causes an increase in the G0/G1 phase and cell cycle arrest in the G2/M phase dependent on time. Our results demonstrated that the treated cells showed an arrest at G2/M when compared with the control group. Thus, the **4o** molecule induces cell cycle arrest in the MCF-7 cell line in the G2/M phase. The arrest also determined by analysis of array experiments which confirms cell cycle experiments.

### Antibacterial analysis

In our investigation, the antimicrobial activity of compound **4o** was rigorously assessed against two prominent bacterial strains, *S. aureus* and *E. coli*, alongside a fungal strain, *C. albicans*. The MIC values for both bacterial strains were found to be 1.56 µM, indicating the lowest concentration at which the compound inhibited visible growth. Furthermore, the MBC values mirrored the MIC values, signifying that the compound not only restrained bacterial growth but also demonstrated bactericidal properties at the same concentration. Remarkably, the antifungal activity against *C. albicans* exhibited an even lower MIC of 0.39 µM, highlighting the potent efficacy of compound **4o** against this fungal strain. These findings underscore the broad-spectrum antimicrobial potential of compound **4o**, positioning it as a promising candidate for further exploration in the development of novel antimicrobial agents. In Table 3 the summary of the antimicrobial results were presented accordingly. The compound is effective for anticancer and antimicrobial activity and thus, the result suggests that 4o may use similar biochemical processes at *S. aureus*, *E. coli*, *C. albicans*. Apoptosis and cell cycle experiments indicate that 4o may affect DNA synthesis, replication and related pathways. Therefore, similar patterns may be seen in the evolutionary processes that result in the emergence of adaptive phenotypes in prokaryotic and eukaryotic cells in response to the selection pressure of therapeutic agents/drugs.

**Table 3:** MIC and MBC values of Compound **4o**

	4o		Positive control	
Microbial Strains	MIC (µM)	MBC (µM)	MIC (µM)	MBC (µM)
*S. aureus*	1.56	1.56	0.63^a^	63.0^a^
*E. coli*	1.56	1.56	5.09^a^	50.9^a^
*C. albicans*	0.39	0.39	1.63^b^	16.3^b^

^a^ Doxycyline and ^b^ fluconazole

### Molecular docking analysis

Since it enables better knowledge of the structure and the drug action mechanisms on body functions at the cellular and molecular levels, computational chemistry and modeling are now frequently used to develop and discover pharmaceuticals. Additionally, this is connected to decreased expenses and chemical dangers while synthesizing these molecules [76]_._ From this point of view, it is possible to say how important the molecular docking process is. If this technique is briefly explained, a molecular docking simulation technique looks at the ideal position for a ligand to bind to a target's active site. In this method, the binding site in the target is chosen using 3D coordinates, and the binding affinity of the resulting orientation of the molecule within the binding site, which creates the complex, is calculated. The most significant and sensitive binding affinity value is the one with the largest negative number (highest binding affinity or lowest binding energy), representing the most advantageous conformation of the complex created when the involved ligand successfully binds to the active pockets target. In this part, the molecular docking mechanism between the three targets RAC-alpha serine/threonine-protein kinase (AKT-1), phosphatidylinositol 3-kinase (PI3K), and Mammalian target of rapamycin (mTOR) and **4o** ligand were investigated separately. Here, the calculation on the **4o** ligand is based entirely on the *in-vitro* results, and the best results were examined in the experimental environment. While there are a lot of *in-silico* molecular docking systems, the Maestro Schrödinger 2021-3 platform demonstrated superior efficiency for our calculations as the computational tool for molecular docking, Maestro Schrödinger, showcased remarkable precision and accelerated performance, thereby enhancing the accuracy and speed of our analyses [77]. Additionally, it should not be overlooked that it has proven to be reliable in numerous studies and our research [78]. In the initial phase, our focus was on ensuring the reliability of the docking procedure and validating the software employed. To verify the accuracy of ligand binding profiles, we calculated the root mean square deviation (RMSD) parameter by superimposing the native ligand present in each target protein, both in its crystallized state and its docked pose.

**Fig. 5:**
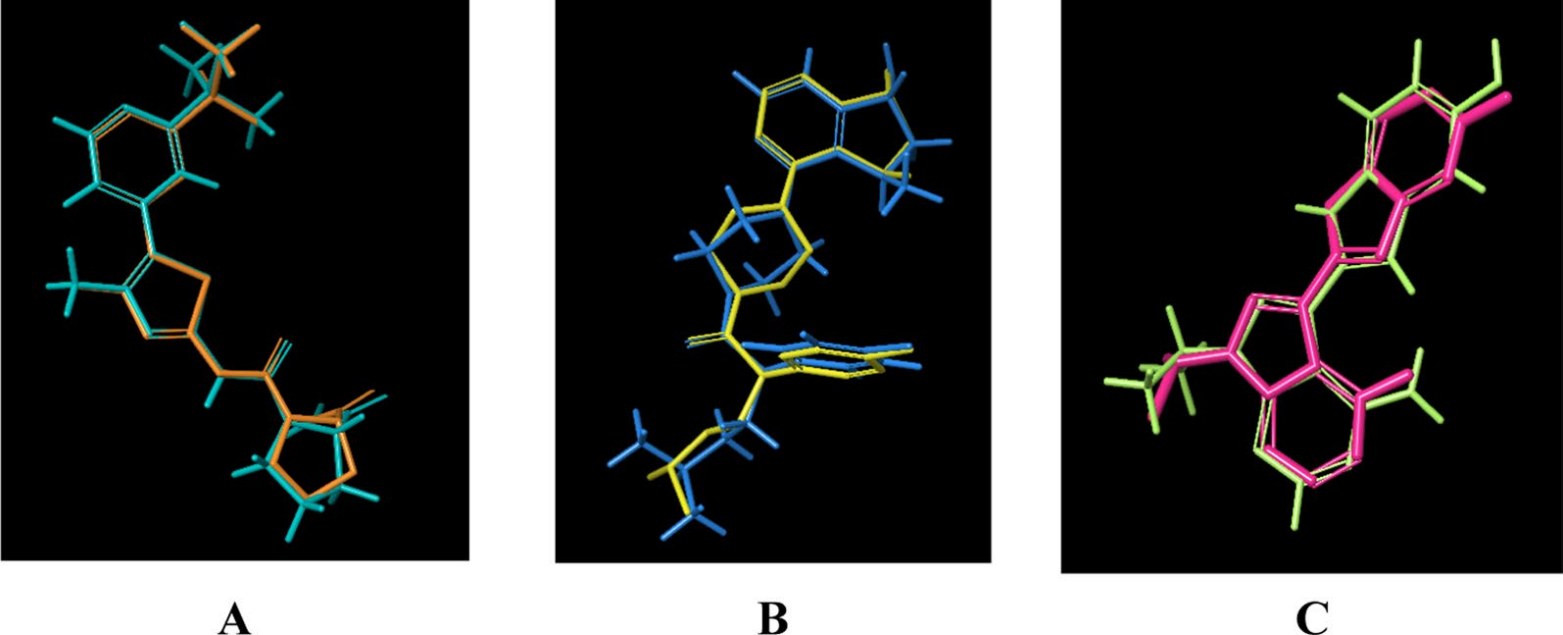
Superimposition of Crystal and Docked Structures for Alpelisib (**A**), Ipatasertib (**B**), and Torkinib (**C**), Native Ligands of PI3K (PDB ID: 4JPS), AKT-1 (PDB ID: 4EKL), and mTOR (PDB ID: 4JT5) Targets

**Fig. 6:**
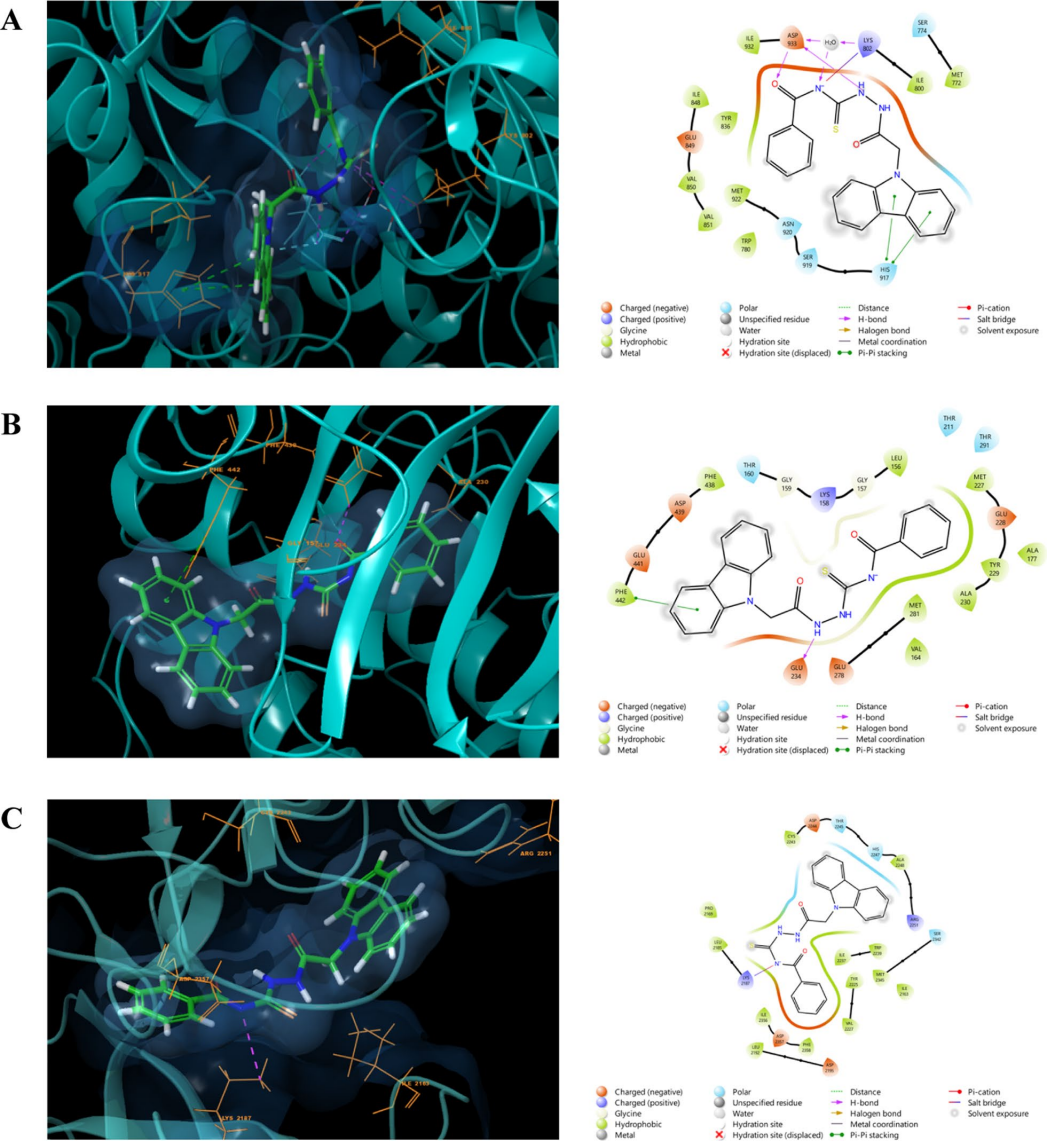
Docking simulations of compound 4O within the binding sites (light blue color) of (**A**) PI3K (PDB ID: 4JPS), (**B**) AKT-1 (PDB ID: 4EKL), and (**C**) mTOR (PDB ID: 4JT5), presented in both 2D and 3D structures. Hydrogen bonds and salt bridges are depicted in magenta, while π-cationic interactions are highlighted in green

**Fig. 7:**
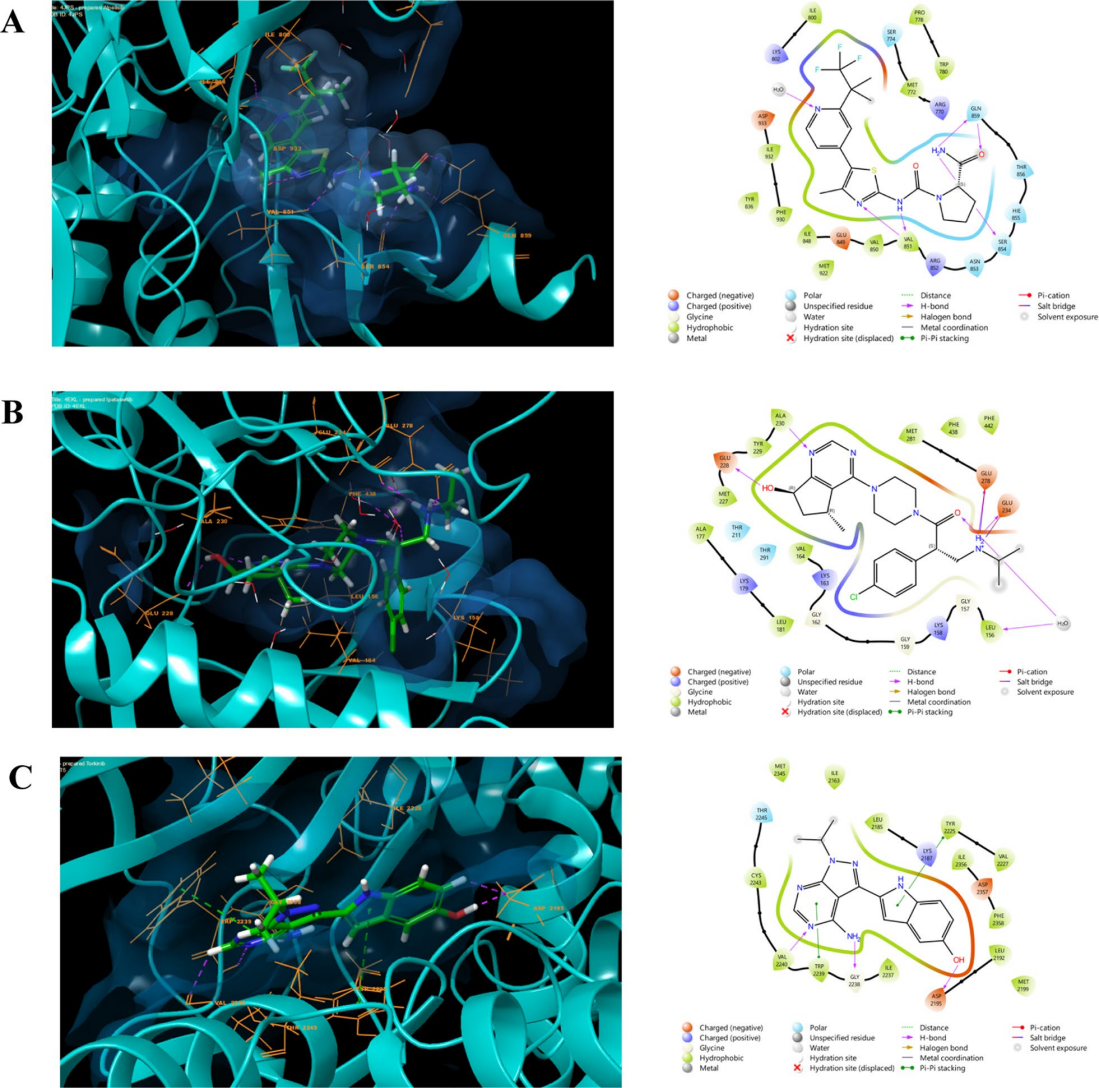
Docking simulations of the native ligands Alpelisib (**A**), Ipatasertib (**B**), and Torkinib (**C**), within their respective targets PI3K (PDB ID: 4JPS), AKT-1 (PDB ID: 4EKL), and mTOR (PDB ID: 4JT5) Targets

The RMSD values serve as crucial indicators, where a value below 2 Å signifies the reasonability and precision of the applied docking procedure. In Fig. 5, the superimposition of Alpelisib, Ipatasertib, and Torkinib structures—native ligands for the PI3K (PDB ID: 4JPS), AKT-1 (PDB ID: 4EKL), and mTOR (PDB ID: 4JT5) targets—over their respective docked poses is illustrated. The resulting RMSD values were 0.3622, 0.555, and 0.2666 Å, respectively. As depicted in the figure, these values substantiate the robustness and accuracy of the employed docking procedure and software. Additionally, the docking simulations depicted in Fig. 7 showcase the optimal accommodation of the native ligands within their respective binding sites. These ligands establish multiple valuable physical interactions with surrounding residues, such as hydrogen bonds, salt bridges, and hydrophobic interactions, as summarized in Table 4. The observed binding profiles align closely with those reported in the literature [79–81] (Fig. 6).

**Table 4:** The docking scores and ligand-receptor binding profiles of **4o** compound within the binding pockets of PI3K, AKT1, and mTOR receptors

Target	Ligand	Ligand-receptor binding profile				Docking score
		Hydrogen bonds and salt bridges	π-π Stacking	π-Cationic interaction	Hydrophobic interactions	
PI3K	**4o**	Asp933, Lys802	–	His917	Ile800, Ile848, Asn920, Ile932	− 8.9
	Alpelisib	Val851, Ser854, Gln859	–	–	Ile800, Tyr836, Ile848, Val851, Ile932, Asp833	− 12.42
AKT1	**4o**	Glu234	Phe442	–	Leu156, Val164, Ala177, Phe438	− 7.6
	Ipatasertib	Glu228, Ala230, Glu234, Lys278, Leu156	–	–	Thr211, Glu278, Phe442	− 11.83
mTOR	**4o**	Lys2187	–	–	Tyr2225, Ile2237, Asp2244, Thr2245, Ala2248, Ile2356	− 7.2
	Torkinib	Val2240, Gly2238, Asp2195	Tyr2225, Trp2239	–	Tyr2225, Ile2237, Trp2239, Thr2245, Ile2356	− 9.9

In an effort to elucidate the observed anticancer activity and validate the inhibitory potency of compound **4o** against the PI3K/Akt1/mTOR pathway, docking simulations were conducted. Compound **4o** was docked against the previously optimized crystallographic structures of PI3K, AKT-1, and mTOR targets identified by their respective PDB IDs: 4JPS, 4EKL, and 4JT5. The results of these simulations are presented in Fig. 7.

Figure 7a illustrates the docking of compound **4o** to the binding site of PI3K enzymes, demonstrating the establishment of two hydrogen bonds with the surrounding amino acids Asp933 and Lys802, along with a π-Cationic interaction with the amino acid His917. These crucial interactions are further supported by the formation of additional hydrophobic interactions with enveloping amino acids, including Ile800, Ile848, Asn920, and Ile932. An examination of the binding profile of compound **4o** within the AKT1 protein's binding pocket, as depicted in Fig. 7b, reveals the formation of a hydrogen bond and π-π stacking interaction with Glu234 and Phe442 amino acids, respectively. The significant role of hydrophobic interactions also contributes to the overall affinity profile by engaging with Leu156, Val164, Ala177, and Phe438 amino acids. Upon conducting docking simulations within the binding site of the mTOR target, it is observed that compound **4o** resides within the binding distance, forming a hydrogen bond with the Lys2187 residue (Fig. 7C). Additionally, multiple hydrophobic interactions are identified with surrounding residues such as Tyr2225, Ile2237, Asp2244, Thr2245, Ala2248, and Ile2356 amino acids. These observed binding profiles align with the recorded docking scores, as summarized in Table 4, providing support for the experimentally observed significant inhibition potency of compound **4o** against the PI3K/AKT1/mTOR pathway. The interaction profiles exhibited by compound 4o within the three targeted receptors demonstrate numerous valuable interactions and a fitting geometry akin to that observed for the native ligands. These interactions substantiate its reported activity against the AKT-1/PI3K/mTOR pathway.

### Drug-Likeness and ADME Properties

Early in the drug discovery process, unwanted molecules have often been filtered out using the drug-likeness concept drawn from the structures and characteristics of current medications and drug candidates. So, in short, the drug development process is sped up by the drug-likeness filters based on physicochemical characteristics. Pfizer’s rule of five is a broad principle for establishing drug-likeness and deciding if an inhibitor with specific biological and pharmacological properties would be an orally active medication in the human body [82]. If two or more of these thresholds are met, an inhibitor can be ingested and become active. These principles can be summed up as follows: ≤ 500 g/mol for the molecular weight; ≤ 5 for the Moriguchi octanol–water partition coefficient; ≤ 10 and ≤ 5 for the H-bond acceptor and donor; ≤ 10 for the number of rotational bonds; and < 140 for the topological polar surface area. This section investigated these parameters using the SwissADME website [83]. The compounds' Lipinski violation numbers and drug-likeness states were given in the final column of Table 5. These properties are known as physicochemical properties. According to the recorded values, the physicochemical characteristics of the newly synthesized molecules do not deviate from the Lipinski guidelines and remain within the indicated boundaries. Additionally, the WLOGP values were obtained in the 1.63 (4c)-5.79 (4k) range. It is known that the determined WLOGP value for molecules here is related to lipophilicity from the literature [84].

**Table 5:** Important computed physicochemical properties of the **4a-z** series

Compound	MW	n_Rot_	HBA	HBD	Mlog *P*	TPSA	WLOGP	Lipinski rule violation; drug-likeness
4a	374.46	7	1	3	3.07	90.18	3.62	0;Yes
4b	394.49	9	2	3	2.37	99.41	3.32	0;Yes
4c	425.55	10	3	3	1.63	102.65	1.63	0;Yes
4d	424.52	7	1	3	3.75	90.18	4.77	0;Yes
4e	419.46	8	3	3	2.16	136.0	3.53	0;Yes
4f	404.48	8	2	3	2.75	99.41	3.63	0;Yes
4g	453.35	7	1	3	3.67	90.18	4.38	0;Yes
4h	408.90	7	1	3	3.56	90.18	4.27	0;Yes
4k	442.46	8	4	3	3.88	90.18	5.79	0;Yes
4m	380.51	7	1	3	3.02	90.18	3.62	0;Yes
4n	388.49	7	1	3	3.29	90.18	3.93	0;Yes
4o	392.47	9	2	3	2.43	107.25	2.82	0;Yes
4p	378.49	9	1	3	2.64	90.18	3.03	0;Yes
4r	380.51	7	1	3	3.02	90.18	3.48	0;Yes
4s	392.52	10	1	3	2.86	90.18	3.22	0;Yes
4t	330.45	8	1	3	1.94	90.18	2.39	0;Yes
4y	338.43	8	1	3	2.26	90.18	2.48	0;Yes
4z	312.39	6	1	3	1.86	90.18	1.92	0;Yes

**Fig. 8:**
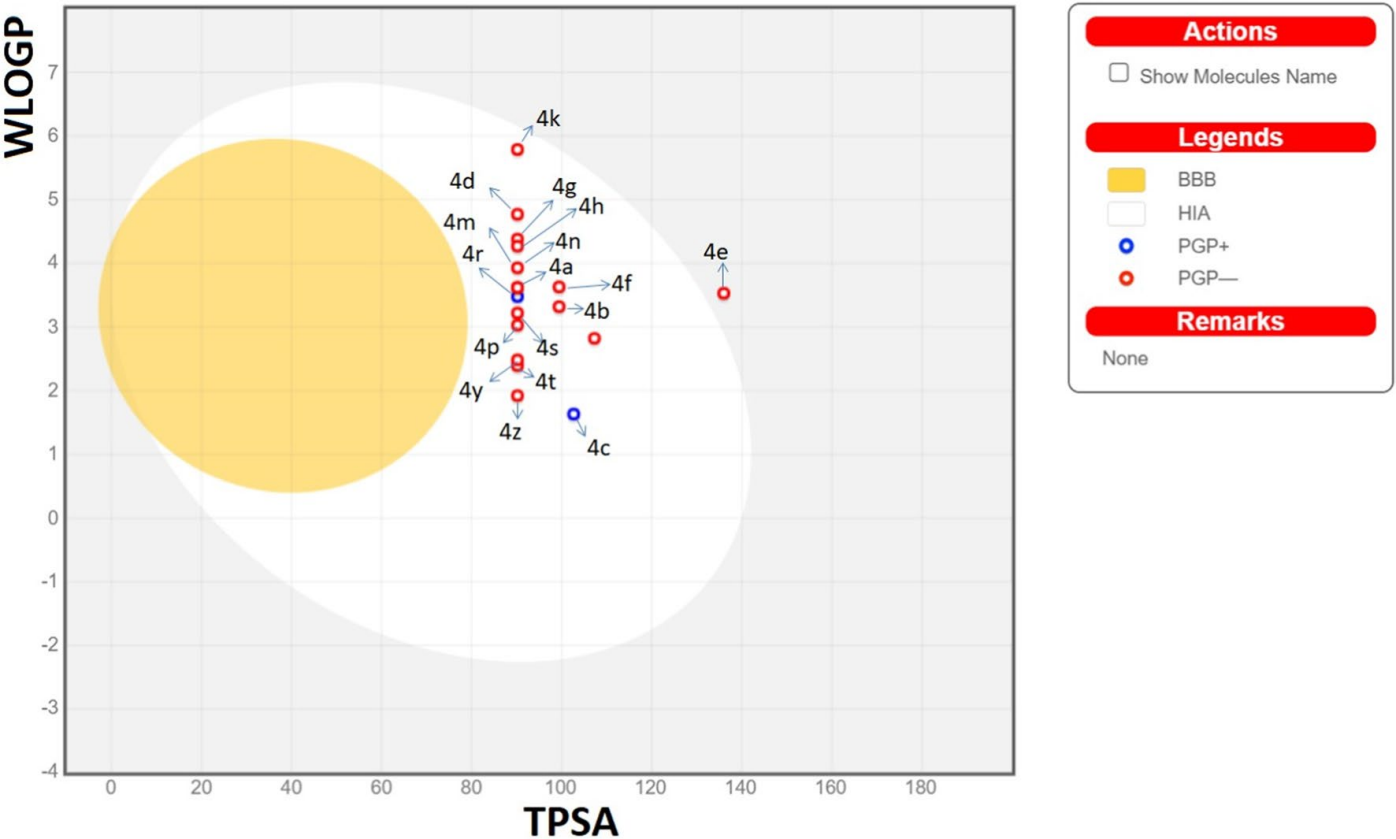
The boiled-egg plots of the **4a-z** molecules

Figure 8 depicts the WLOGP vs TPSA (Boiled-egg plot) plot used to forecast gastrointestinal absorption and brain penetration of the studied compounds **4a-z**. Three colors stand out in the graphic: white, yellow, and gray. The yellow portion (yolk) is for the high probability of brain penetration, and the white region is for the high probability of passive absorption by the gastrointestinal tract. Yolk and white regions are not incompatible [75]. As observed, all of the newly synthesized derivatives, except for **4e**, are in the white region, indicating that all of our chemicals were estimated to be highly absorbed through the GIT by passive diffusion, while they do not have the ability to pass the BBB so could not reach the brain due to being outside the plot’s range.

## Conclusion

In conclusion, this study delved into the biological activities of molecules featuring a carbazole skeleton and thiosemicarbazide functional group, unveiling their potential as therapeutic agents. The synthesized carbazole derivatives showcased diverse activities, encompassing antioxidant and antimicrobial effects, emphasizing their anticancer properties. While compounds **4h** and **4y** exhibited antioxidant activity, this aspect remained uncoupled from anticancer activity, unlike compound **4o**, which demonstrated pronounced anticancer effects. The investigation revealed the ability of these derivatives to potentiate therapeutic anticancer effects by targeting the PI3K/Akt/mTOR signaling pathway, which is crucial for cell signaling, proliferation, survival, and metabolism in breast cancer. Several promising compounds emerged, with compound **4o** standing out as a potential anticancer properties. It modulated the PI3K/Akt/mTOR pathway, inducing apoptosis and halting the cell cycle in MCF-7 cancer cells. Targeting this pathway presents a promising approach to suppressing tumor survival and overcoming drug resistance. This compound showed significant antimicrobial activities against *S. aureus* and *E. coli* bacterial strains. Furthermore, molecular docking studies, particularly on compound **4o**, provided valuable insights into their potential as anticancer agents. Favorable binding profiles and interactions within the binding sites of key enzymes—PI3K, AKT1, and mTOR—underscored the compound's promising affinity for critical components of the PI3K/Akt/mTOR signaling pathway. Additionally, the compounds exhibited drug-like properties, meeting Lipinski's criteria, indicating their potential as lead agents for further drug development. This research contributes significantly to understanding the biological activities of carbazole derivatives, providing insights for designing and synthesizing more potent drug candidates targeting antioxidant and anticancer conditions. Given the pivotal role of the PI3K/Akt/mTOR pathway in cancer development and progression, the antioxidant effects of carbazole derivatives offer a potential therapeutic strategy to combat cancer. The study advocates for further in vivo and in vitro assessments to validate and optimize potential therapeutic agents for future clinical applications. In summary, exploring carbazole derivatives and their activities unveils new avenues for developing effective drugs against cancer and related health conditions, offering hope for improved treatment options in the battle against cancer.

## Supplementary Information

Supplementary Material 1

## Data Availability

All data generated or analyzed during this study are included in this published article (and its supplementary information files), the proteins utilized in our molecular docking study were obtained directly from the PDB databank server (http://www.rcsb.org/pdb).

## Abbreviations

IC_50_: Half maximal inhibitory concentration
MCF-7: A breast cancer cell line
PI3K: Phosphoinositide 3-kinases
Akt: Protein kinase B
mTOR: The mammalian target of rapamycin
HOP-92: A human Non-Small Cell Lung tumor cell line
SK-MEL-2: A cell line isolated from the skin of a 60-year-old, male malignant melanoma patient that can be used in cancer research
PC12: A cell line that was derived from a transplantable rat pheochromocytoma
HepG2: A cell line exhibiting epithelial-like morphology
EGFR: Epidermal growth factor receptor
ROS: Reactive oxygen species
DPPH: 2,2-Diphenyl-1-picrylhydrazyl
RNA: Ribonucleic acid
TLC: Thin-layer chromatography
TMS: Tetramethylsilane
NMR: Nuclear Magnetic Resonance
DMSO: Dimethyl sulfoxide
UPLC/MS-TOF: Ultra-high performance liquid chromatography with quadrupole time-of-flight mass spectrometry
HRMS: High-resolution mass spectrometry
DMF: Dimethylformamide
EtOAc: Ethyl acetate
NaH: Sodium Hydride
Mp: Melting point
MTT: 3-(4,5-Dimethylthiazol-2-yl)-2,5-Diphenyl Tetrazolium Bromide
DMEM: Dulbecco’s Modified Eagle Medium High Glucose
FBS: Fetal bovine serum
PBS: Phosphate Buffered Saline
SAR: Structure activity relationship
L929: An adherent type of mouse fibroblast cell line
