# Correction to: Synthesis of novel carbazole hydrazine‑carbothioamide scaffold as potent antioxidant, anticancer and antimicrobial agents

**DOI:** 10.1186/s13065-024-01274-4

**Published:** 2024-09-16

**Authors:** İrfan Çapan, Mohammed Hawash, Mohammed T. Qaoud, Levent Gülüm, Ezgi Nurdan Yenilmez Tunoglu, Kezban Uçar Çifci, Bekir Sıtkı Çevrimli, Yusuf Sert, Süleyman Servi, İrfan Koca, Yusuf Tutar

**Affiliations:** 1 Department of Pharmaceutical Basic Sciences, Faculty of Pharmacy, Gazi University, 06330 Ankara, Türkiye; 2 Sente Kimya Research and Development Inc., 06200 Ankara, Türkiye; 3 Department of Pharmacy, Faculty of Medicine and Health Sciences, An-Najah National University, Nablus, Palestine; 4 Department of Pharmacy, Faculty of Pharmacy, Cyprus International University, Northern Cyprus, Mersin 10, 99258 Nicosia, Türkiye; 5 Department of Plant and Animal Production, Mudurnu Süreyya Astarcı Vocational College, Bolu Abant İzzet Baysal University, Bolu, Türkiye; 6 Department of Medical Laboratory Techniques, Vocational School of Health Services, Demiroğlu Bilim University, Istanbul, Türkiye; 7 Department of Molecular Medicine, Faculty of Health Sciences, University of Health Sciences, Istanbul, Türkiye; 8 Division of Basic Sciences and Health, Hemp Research Institute, Yozgat Bozok University, Yozgat, Türkiye; 9 Department of Chemistry and Chemical Processing Technologies, Technical Sciences Vocational College, Gazi University, Ankara, Türkiye; 10 Sorgun Vocational College, Yozgat Bozok University, Yozgat, Türkiye; 11 Department of Chemistry, Faculty of Science, Fırat University, Elazığ, Türkiye; 12 Department of Chemistry, Faculty of Art & Sciences, Yozgat Bozok University, Yozgat, Türkiye; 13 Medical School, Division of Biochemistry, Recep Tayyip Erdogan University, Rize, Türkiye; 14 Faculty of Pharmacy, Division of Biochemistry, University of Health Sciences, Istanbul, Türkiye

**Correction to: BMC Chemistry (2024) 18:102**

10.1186/s13065-024-01207-1

Following publication of the original article [1], the author noticed the affiliation error in the Supplementary Material which should be like the original published version. It has now been replaced with the correct supplementary file.

The original article has been corrected. 

## Electronic supplementary material

Below is the link to the electronic supplementary material.

Supplementary Material 1
