# Supplementary material for: Synthesis of novel carbazole hydrazine-carbothioamide scaffold as potent antioxidant, anticancer and antimicrobial agents
Source: BMC Chem. 2024 May 21;18(1):102. doi: 10.1186/s13065-024-01207-1 (PMC11110238; doi:10.1186/s13065-024-01207-1)
Supplement: Supplementary file 1 — Supplementary Material 1 [file 13065_2024_1207_MOESM1_ESM.docx]

Supporting Information

**Synthesis of Novel Carbazole Hydrazine-carbothioamide Scaffold as Potent Antioxidant, Anticancer and Antimicrobial agents**

İrfan Çapan ^1,2^*, Mohammed Hawash ^3^*, Mohammed T. Qaoud ^4^, Levent Gülüm ^5^, Ezgi Nurdan Yenilmez Tunoglu ^6^, Kezban Uçar Çifci ^7,8^, Bekir Sıtkı Çevrimli ^9^, Yusuf Sert ^10^, Süleyman Servi ^11^, İrfan Koca ^12^, Yusuf Tutar ^13,14^

^1^ Department of Pharmaceutical Basic Sciences, Faculty of Pharmacy, Gazi University, 06330, Ankara, Türkiye.

^2^ Sente Kimya Research and Development Inc., 06200, Ankara, Türkiye.

^3^ Department of Pharmacy, Faculty of Medicine and Health Sciences, An-Najah National University, Nablus, Palestine.

^4^ Department of Pharmacy, Faculty of Pharmacy, Cyprus International University, Northern Cyprus, Mersin 10, 99258 Nicosia, Türkiye.

^5^ Department of Plant and Animal Production, Mudurnu Süreyya Astarcı Vocational College, Bolu Abant İzzet Baysal University, Türkiye.

^6^ Department of Medical Laboratory Techniques, Vocational School of Health Services, Demiroğlu Bilim University, İstanbul, Türkiye.

^7^ Department of Molecular Medicine, Faculty of Health Sciences, University of Health Sciences, İstanbul, Türkiye.

^8^ Division of Basic Sciences and Health, Hemp Research Institute, Yozgat Bozok University, Yozgat, Türkiye.

^9^ Department of Chemistry and Chemical Processing Technologies, Technical Sciences Vocational College, Gazi University, Ankara, Türkiye.

^10^ Sorgun Vocational College, Yozgat Bozok University, Yozgat, Türkiye.

^11^ Department of Chemistry, Faculty of Science, Fırat University, Elazığ, Türkiye.

^12^ Department of Chemistry, Faculty of Art & Sciences, Yozgat Bozok University, Yozgat, Türkiye.

^13^ Recep Tayyip Erdogan University, Medical School, Division of Biochemistry, Rize, Turkey

^14^ University of Health Sciences, Faculty of Pharmacy, Division of Biochemistry, Istanbul, Turkey.

***Corresponding authors:**

Mohammed Hawash, Department of Pharmacy, Faculty of Medicine and Health Sciences, An-Najah National University, Nablus, Palestine, orcid.org/0000-0001-5640-9700; Phone: +972569939939; Email: [mohawash@najah.edu](mailto:mohawash@najah.edu). İrfan Çapan, Department of Pharmaceutical Basic Sciences, Faculty of Pharmacy, Gazi University, 06330, Ankara, Türkiye, Phone: +90 312 202 2685 Fax: +90 312 202 80 43, E-mail : irfancapan@gazi.edu.tr

#
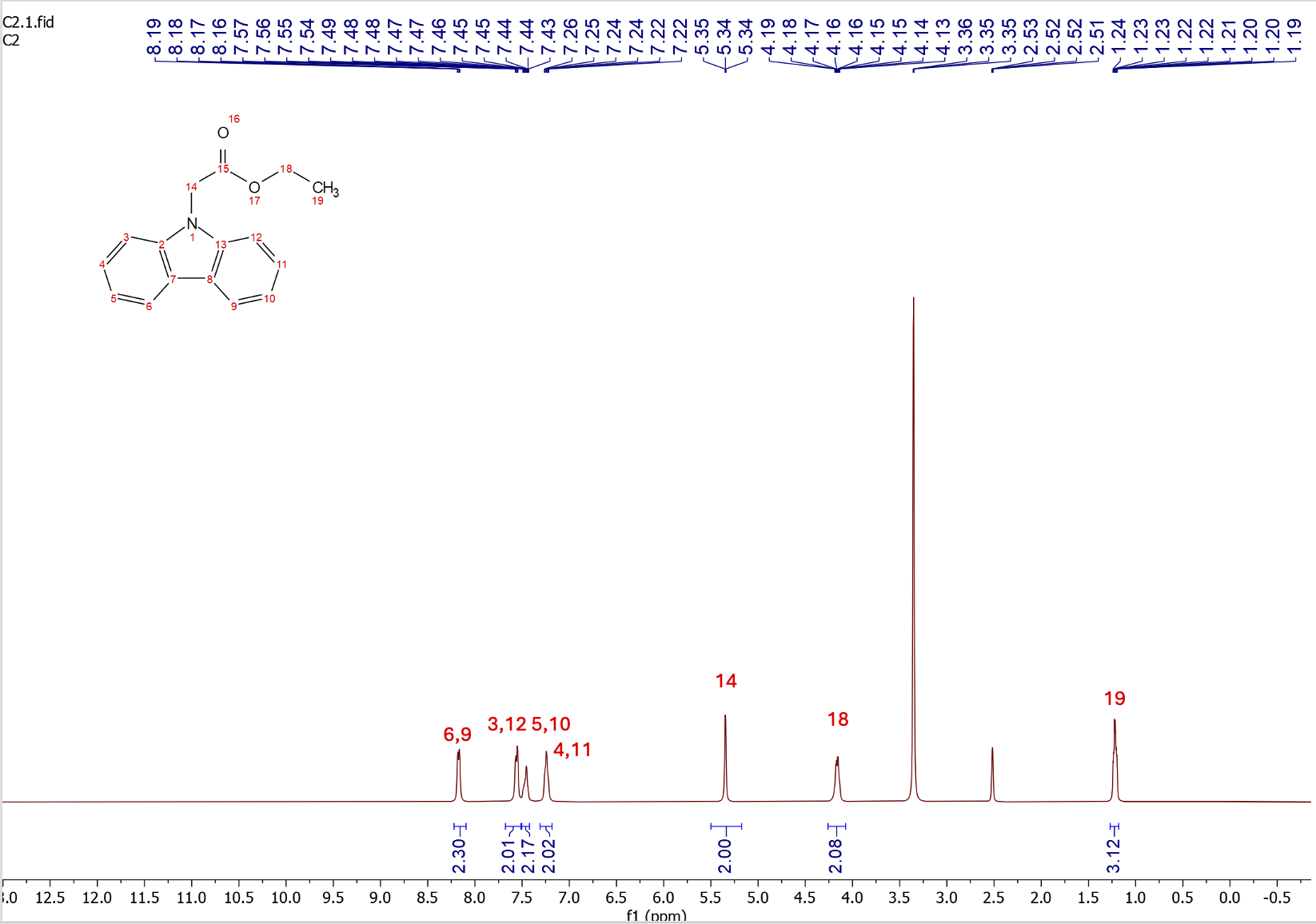
^1^H-NMR Spectrum of Compound 2

#
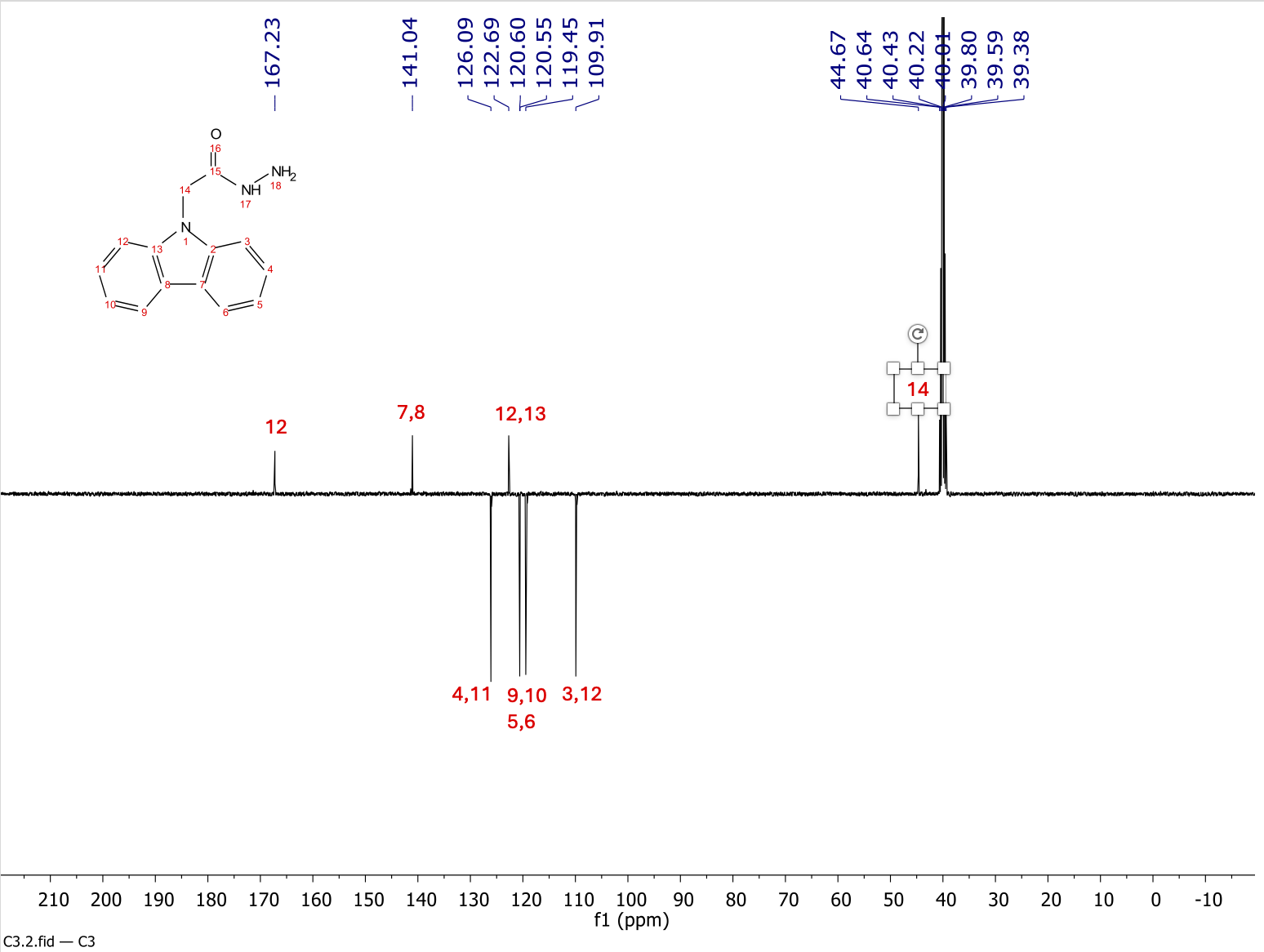

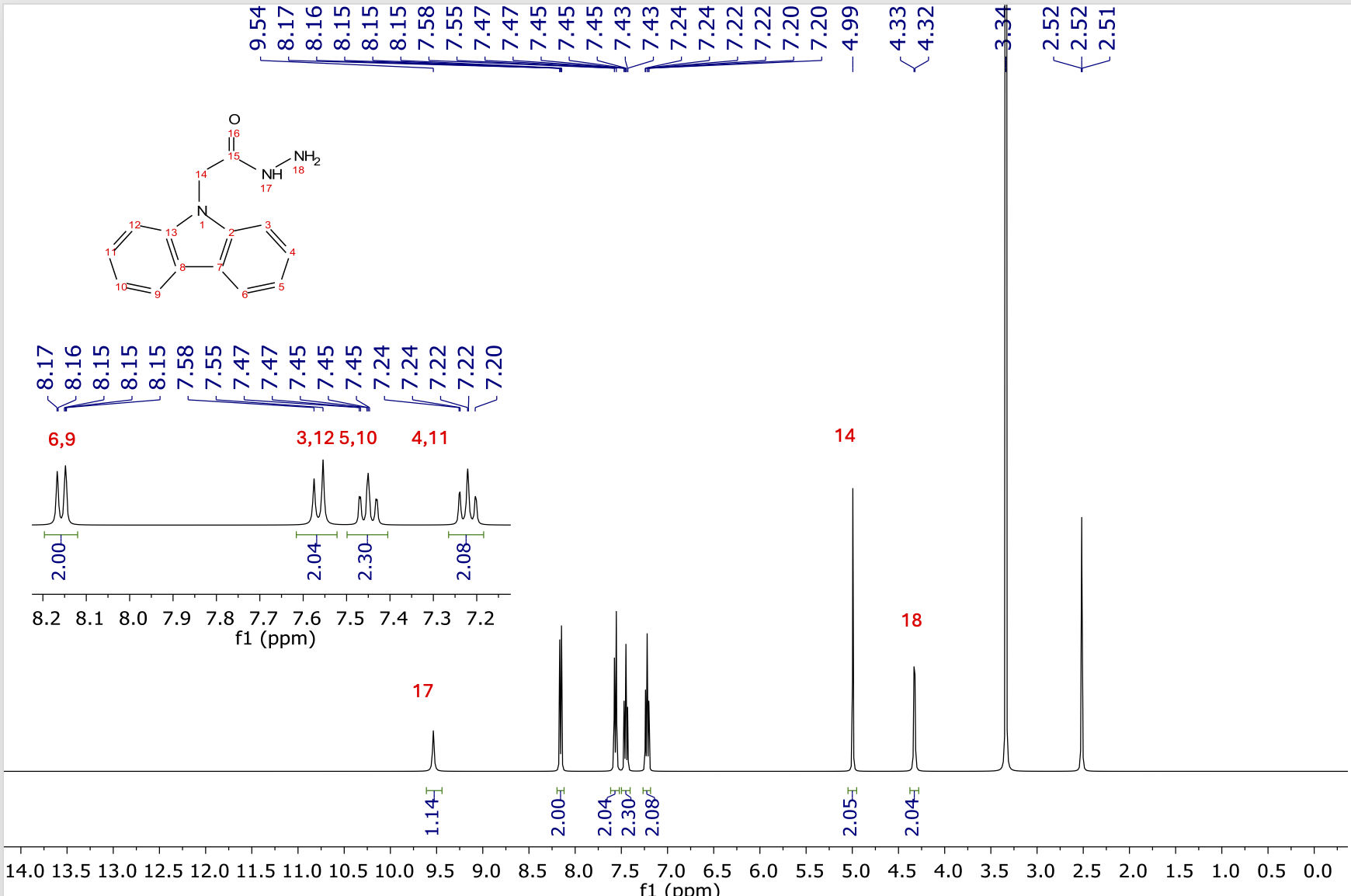
Spectrums of Compound 3

# Spectrums of Compound 4a

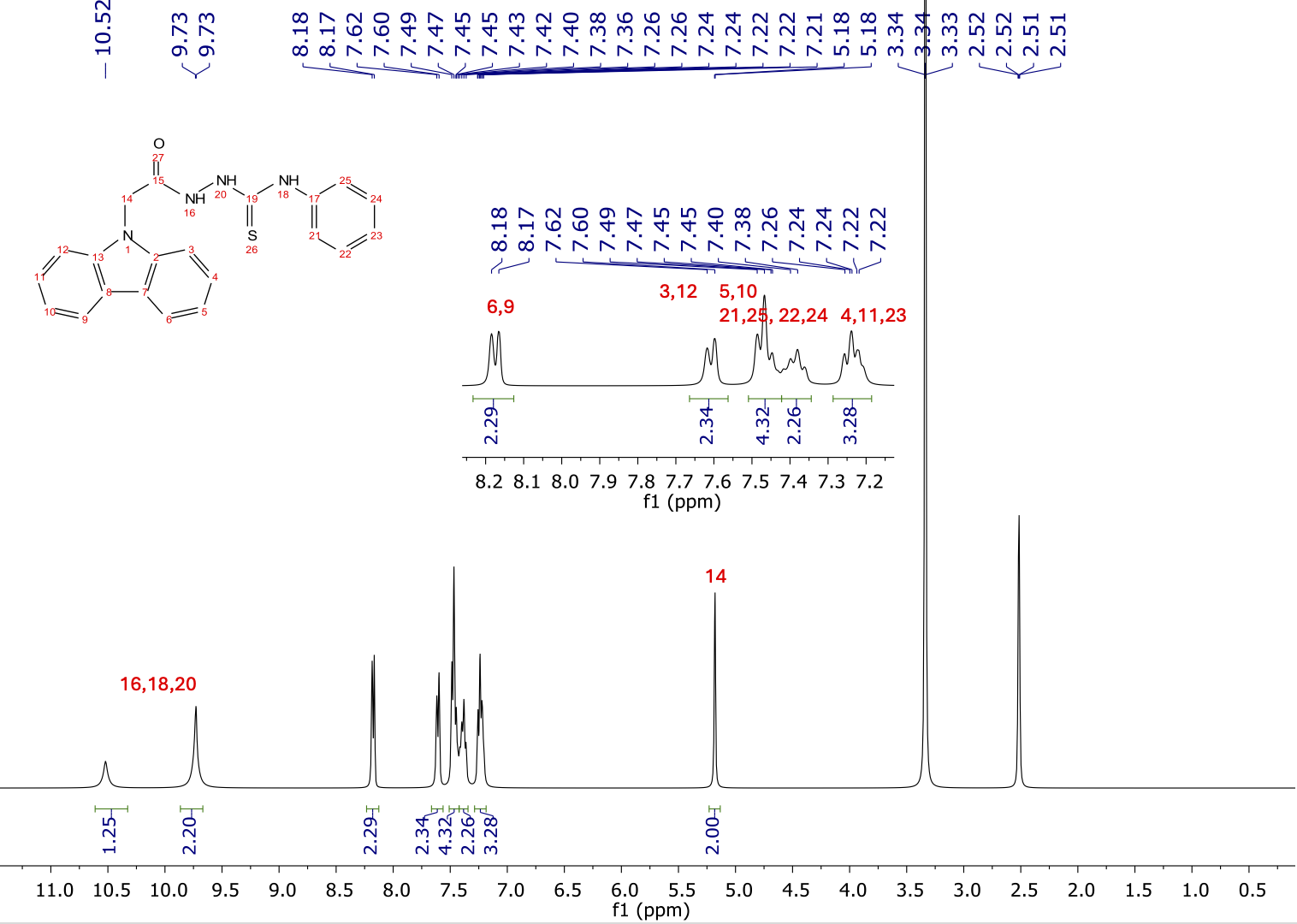


**
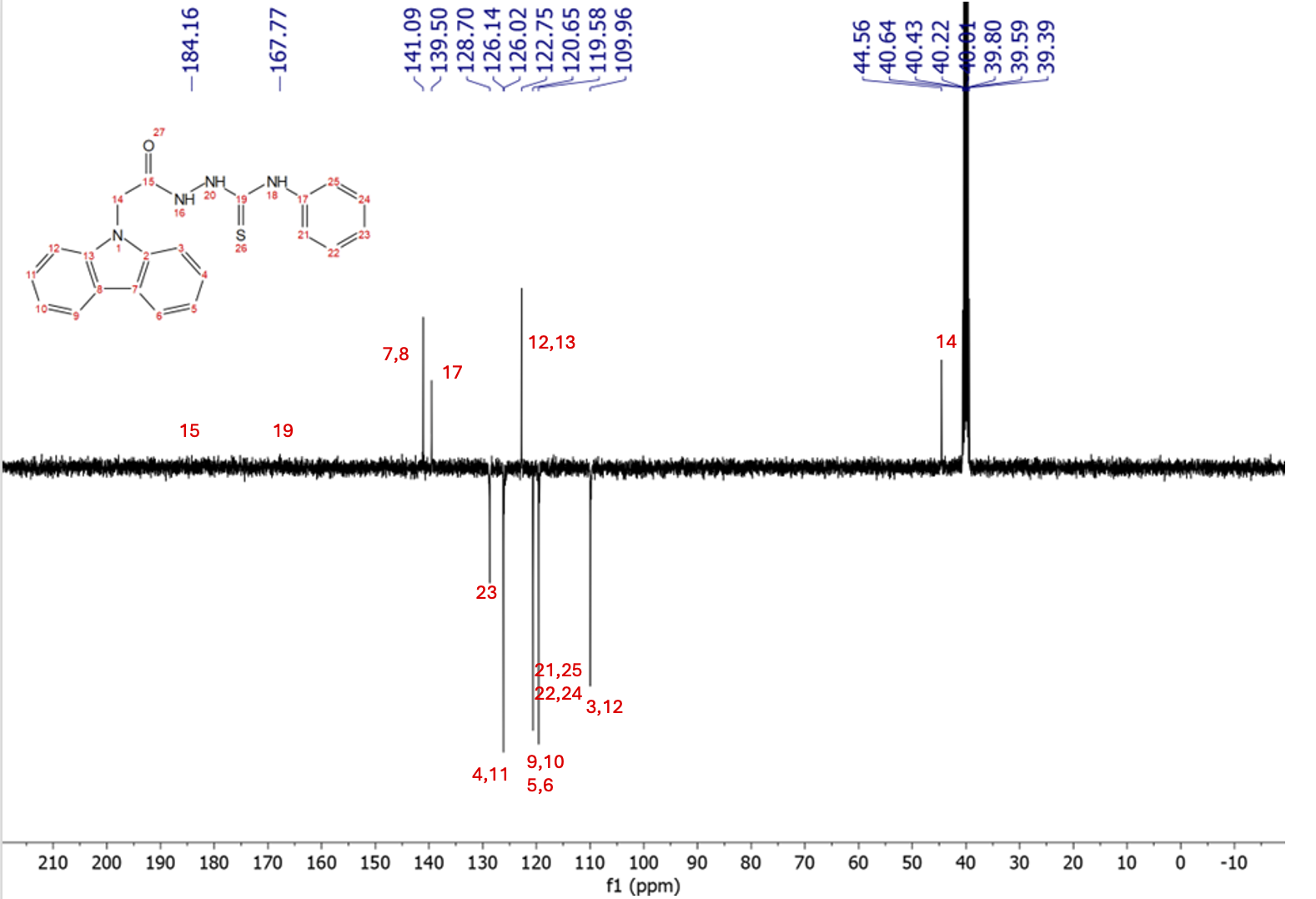
**

# Spectrums of Compound 4b

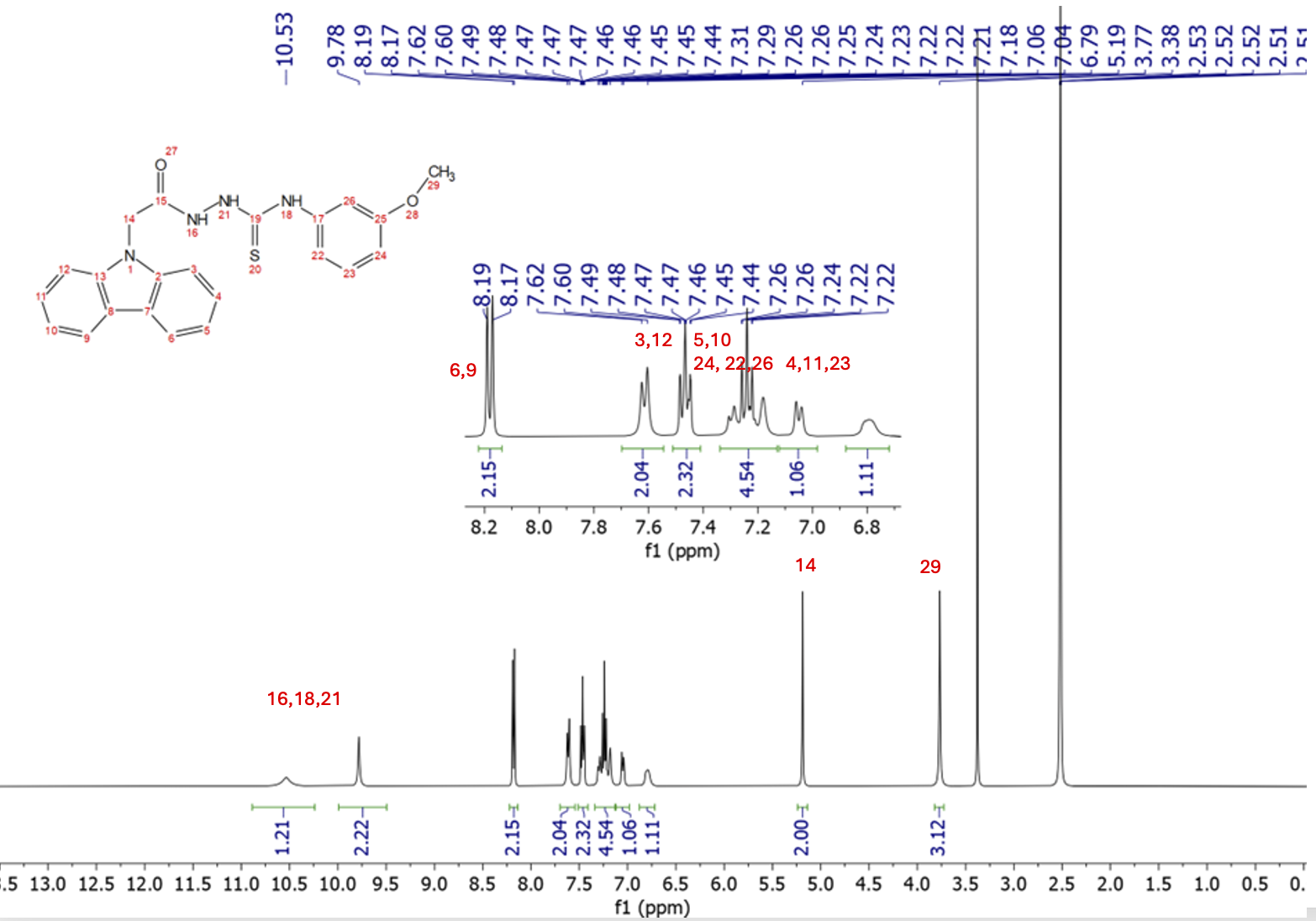


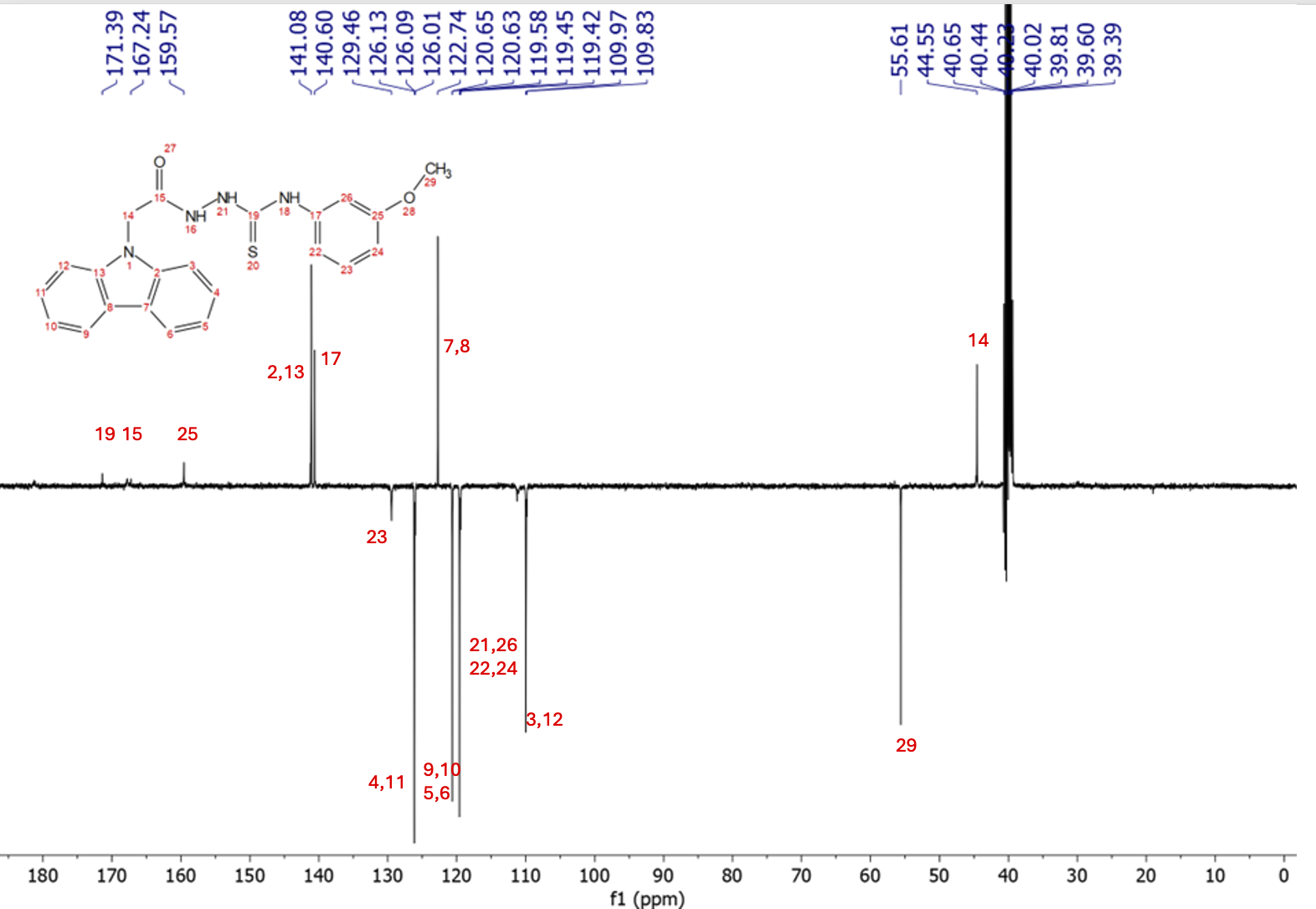


# Spectrums of Compound 4c

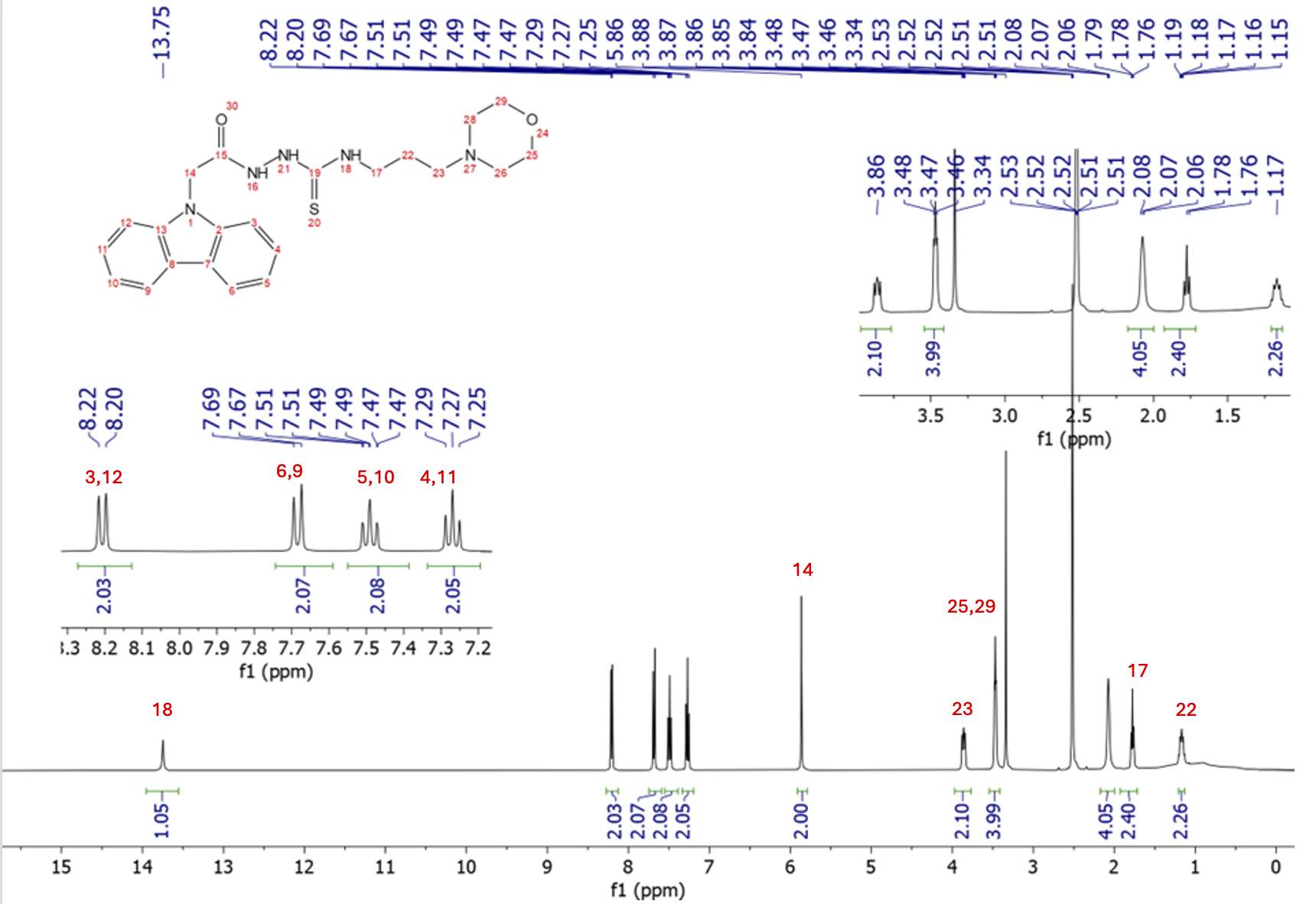


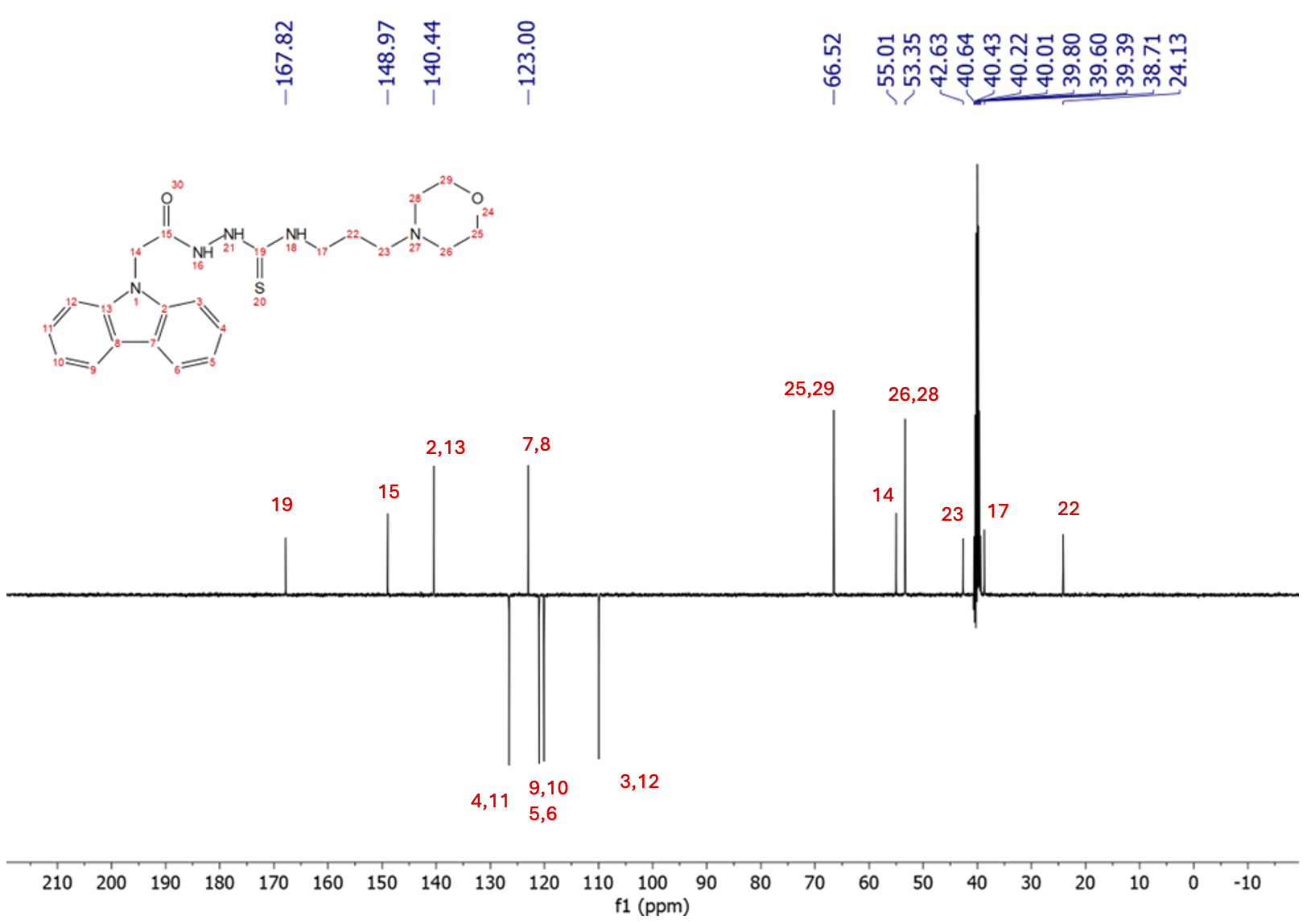


# Spectrums of Compound 4d

**
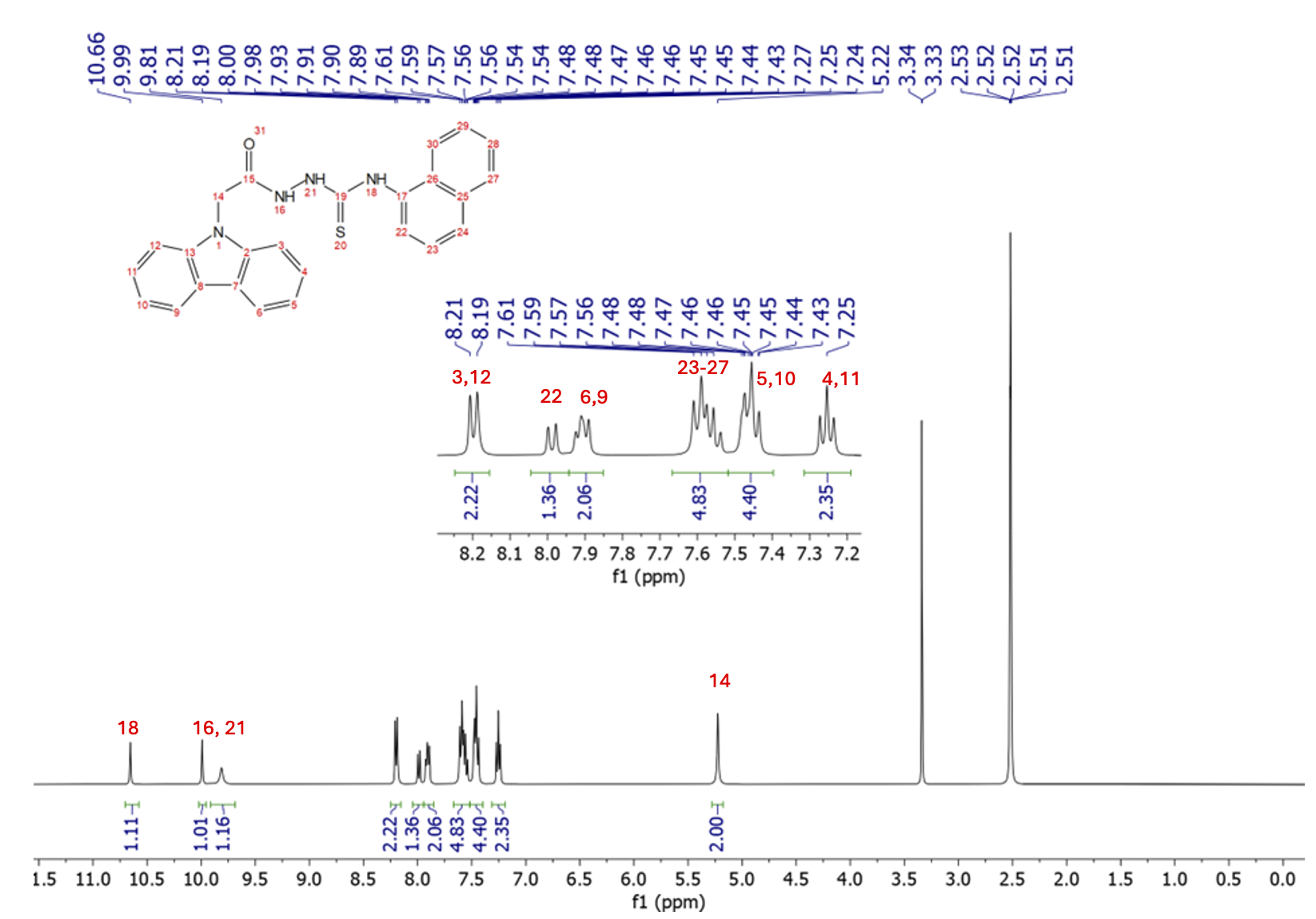
**


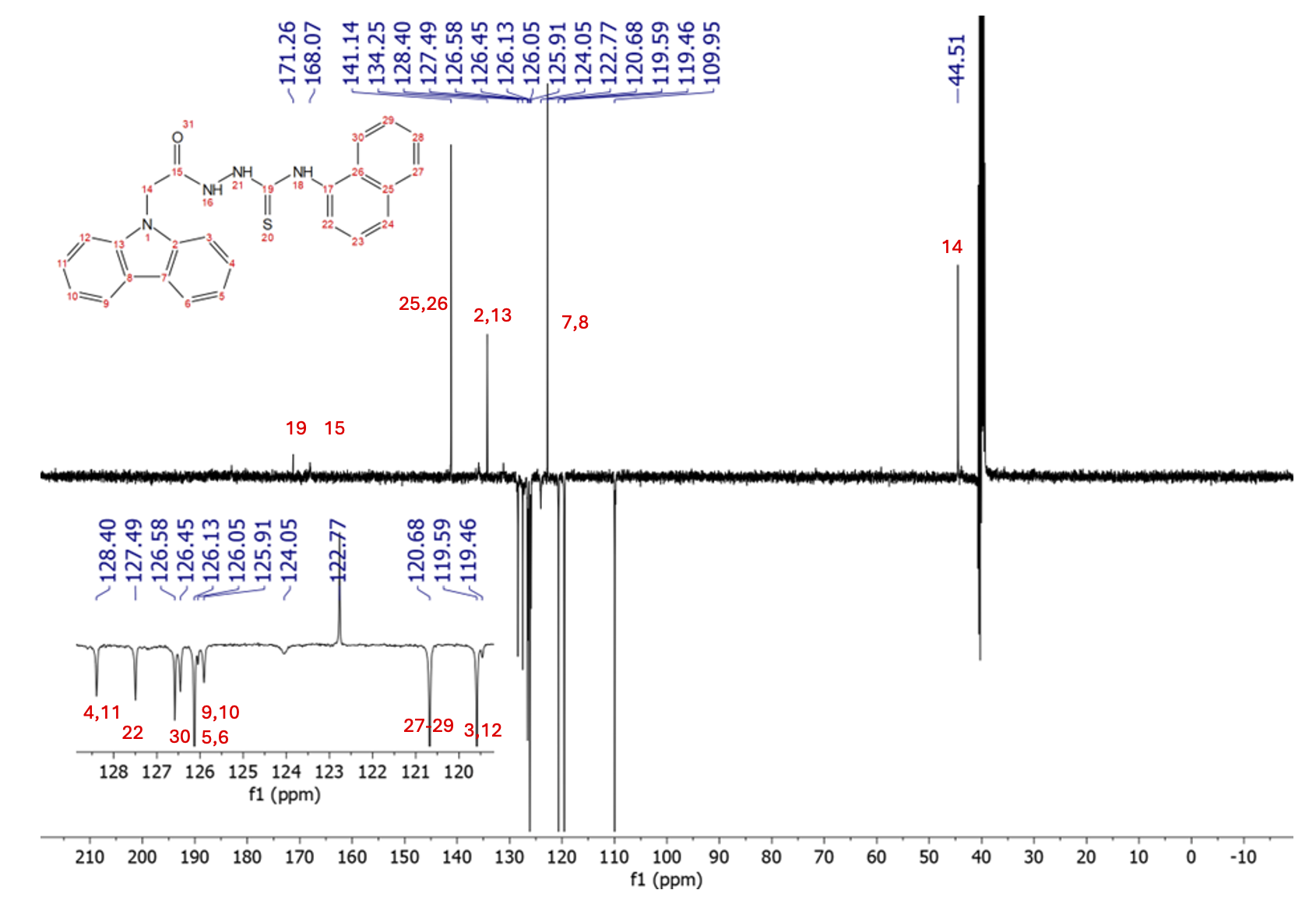


# Spectrums of Compound 4e

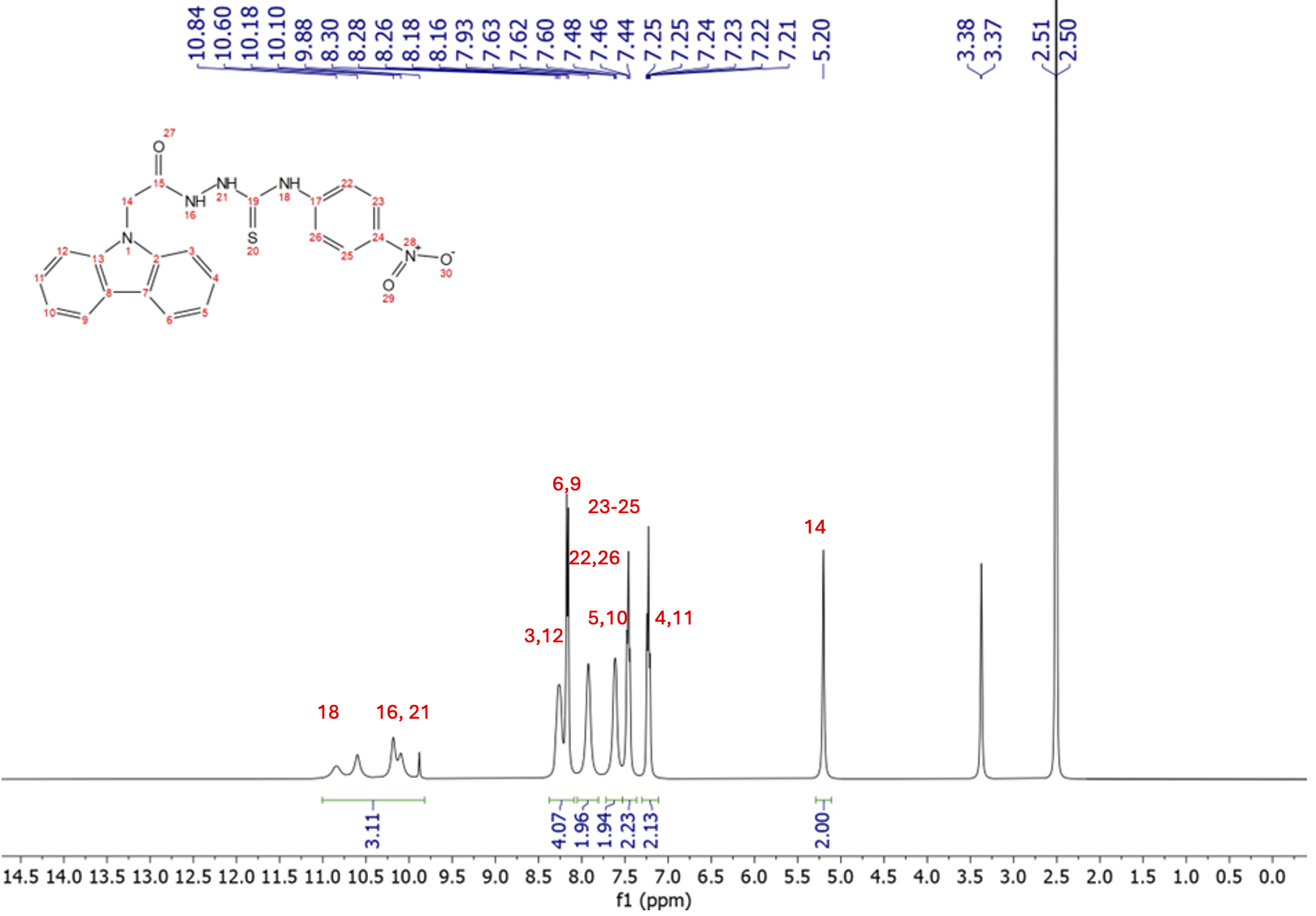


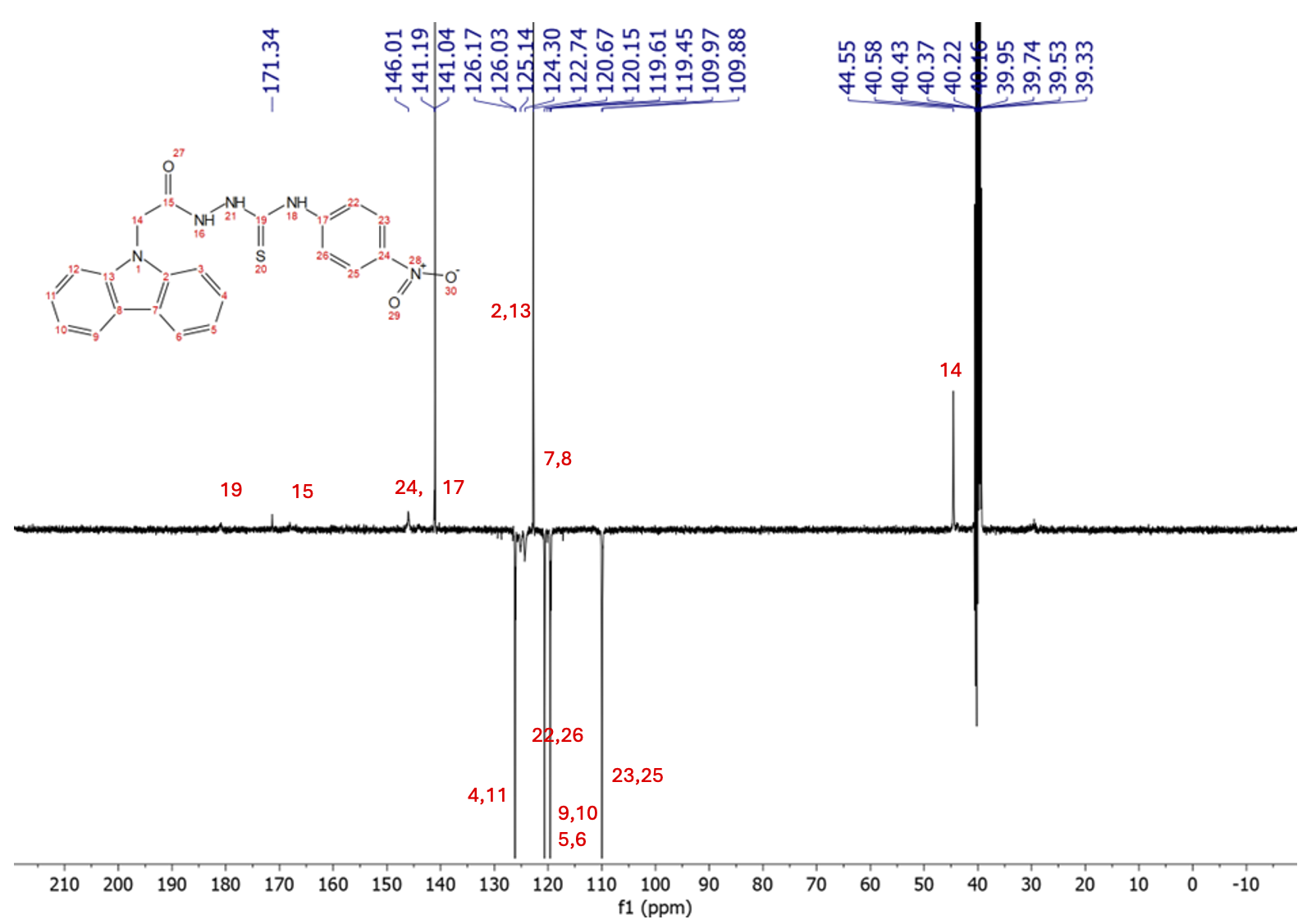


# Spectrums of Compound 4f

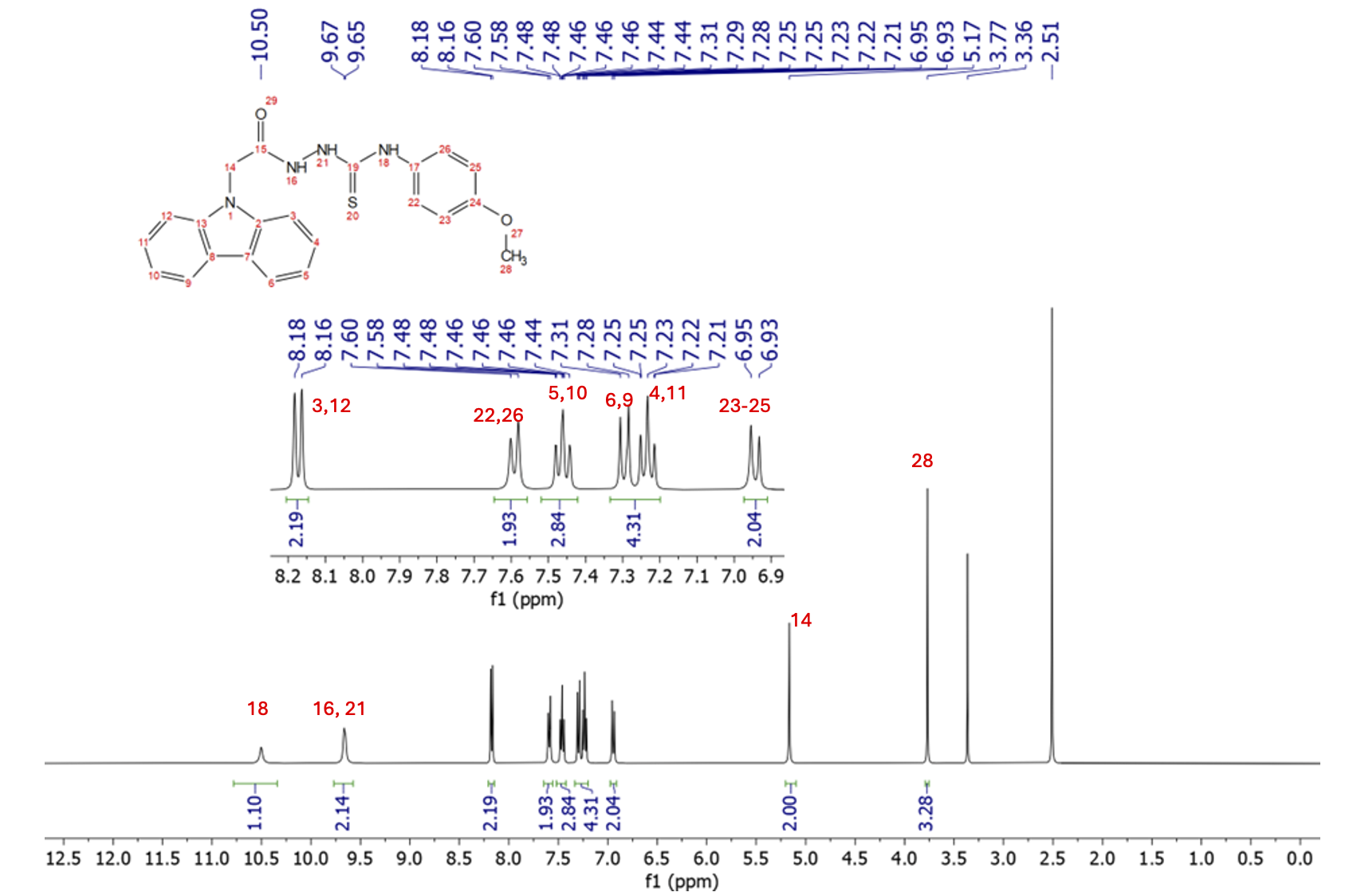


**
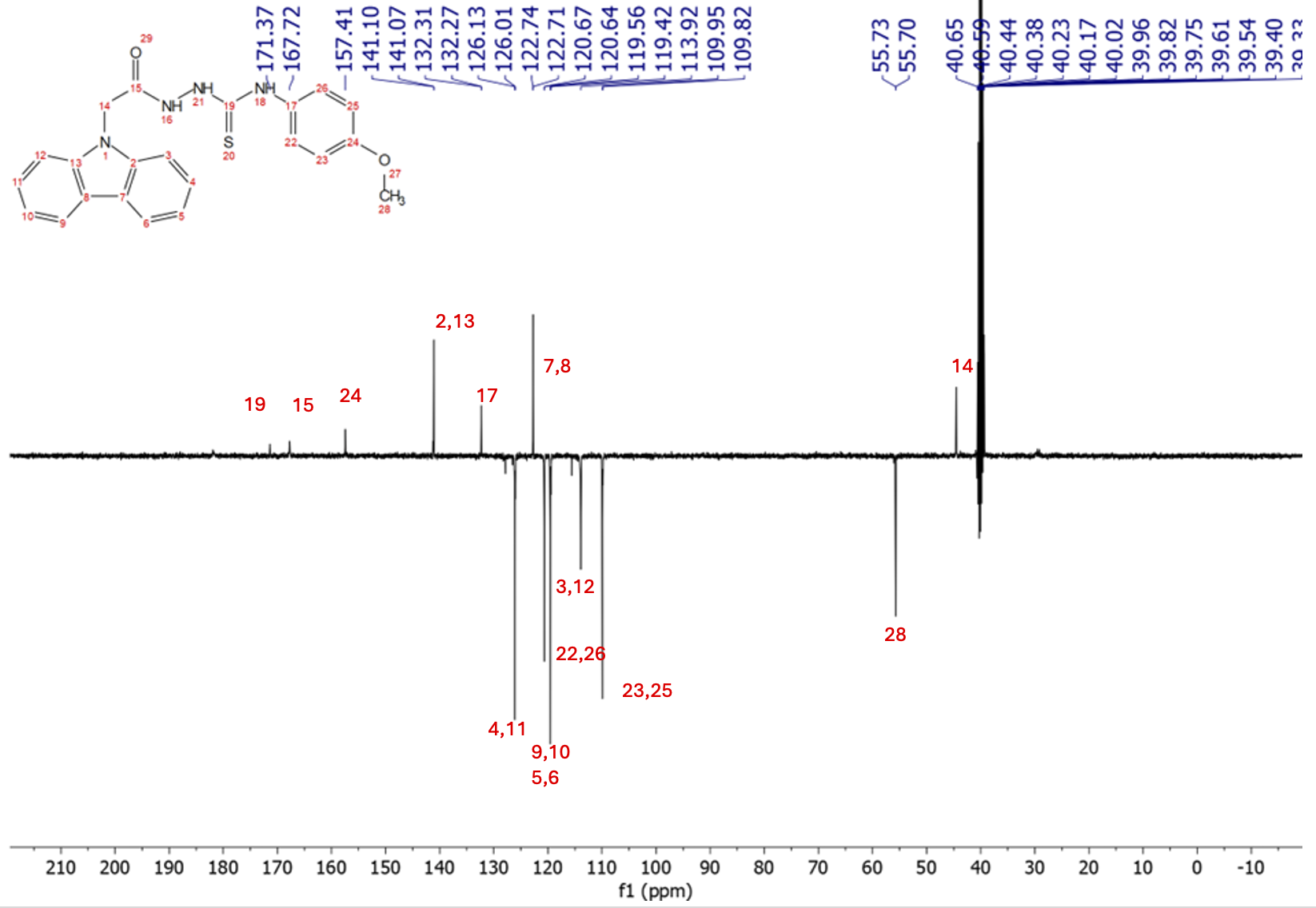
**

# Spectrums of Compound 4g

**
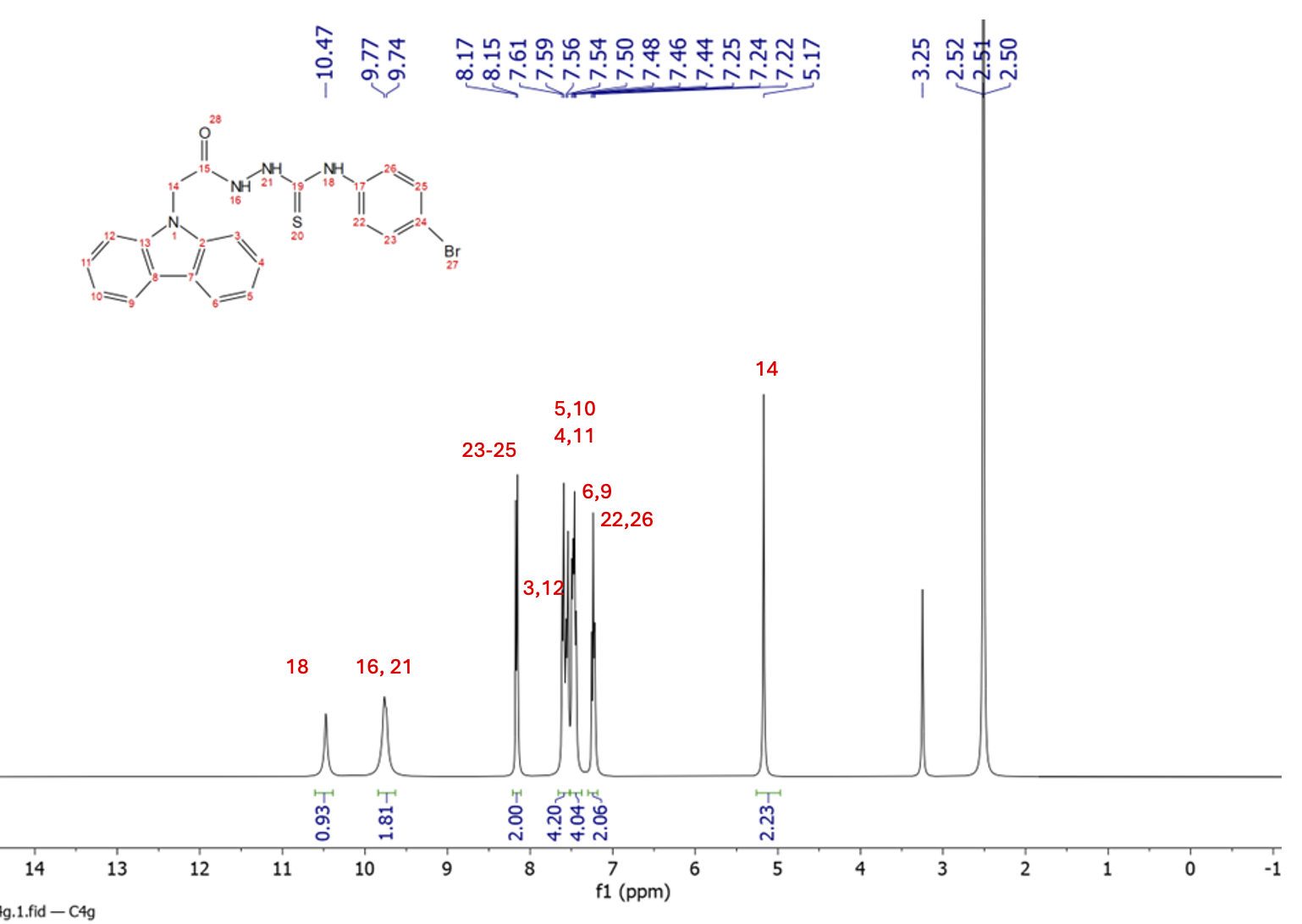
**

**
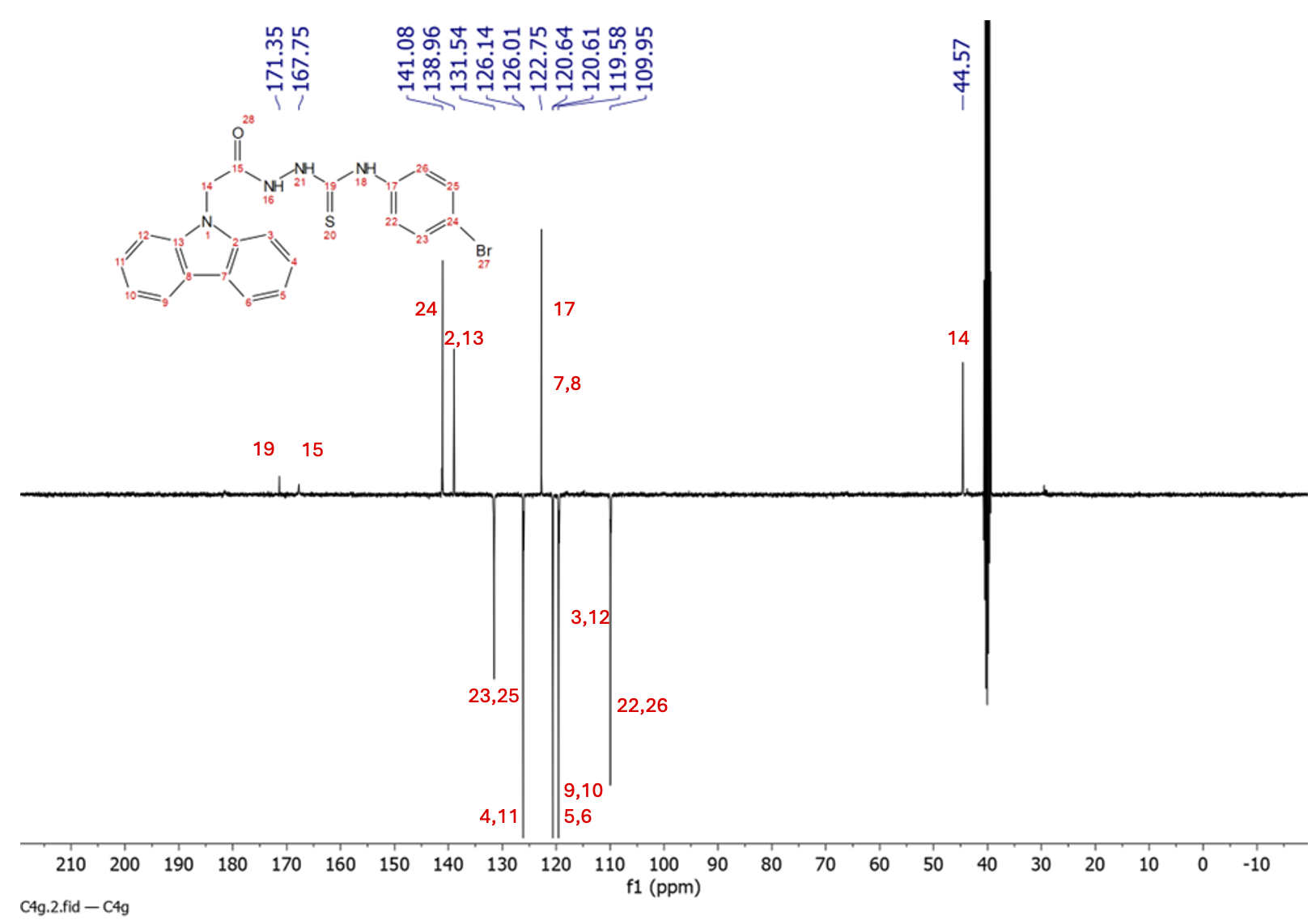
**

# Spectrums of Compound 4h

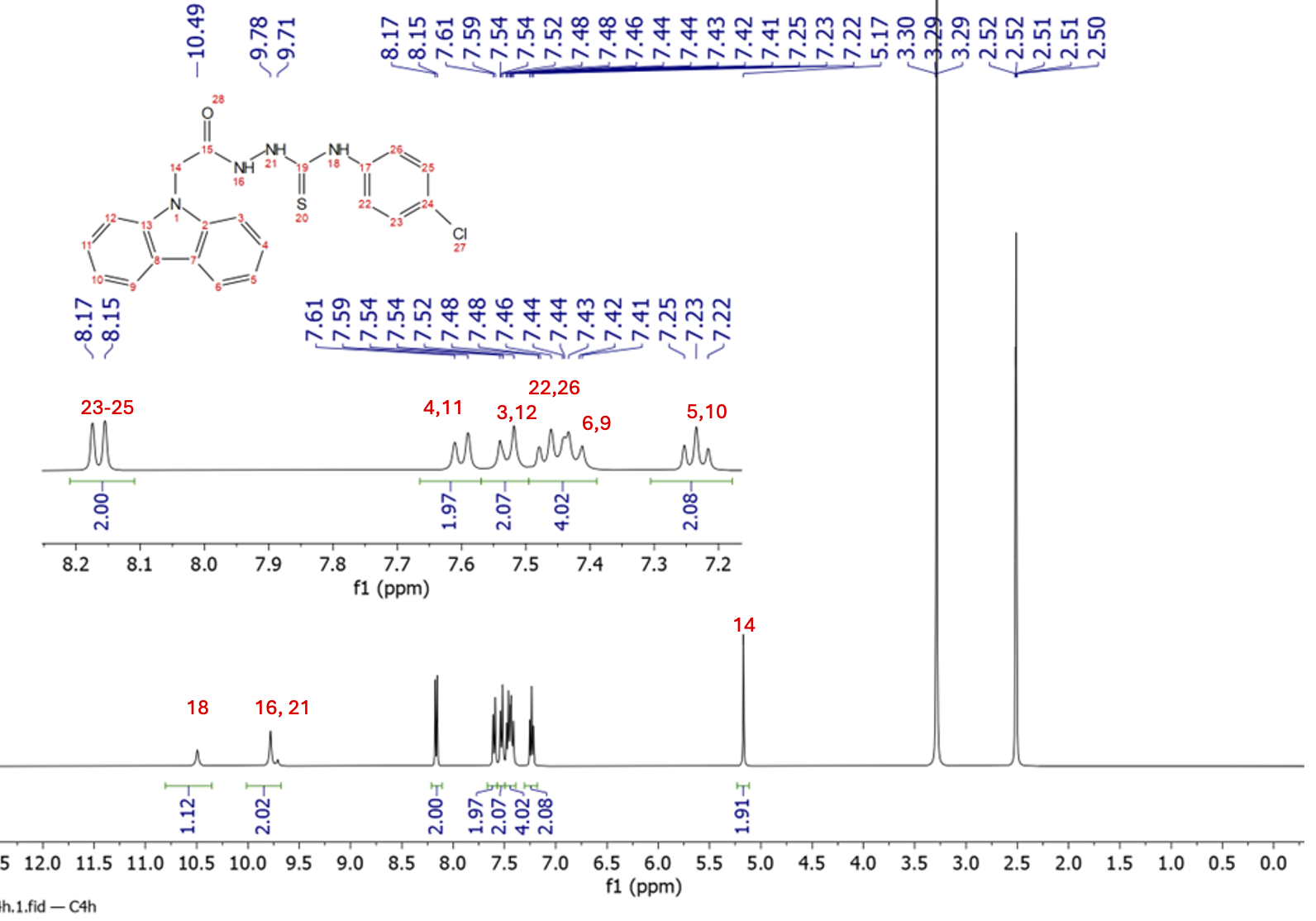


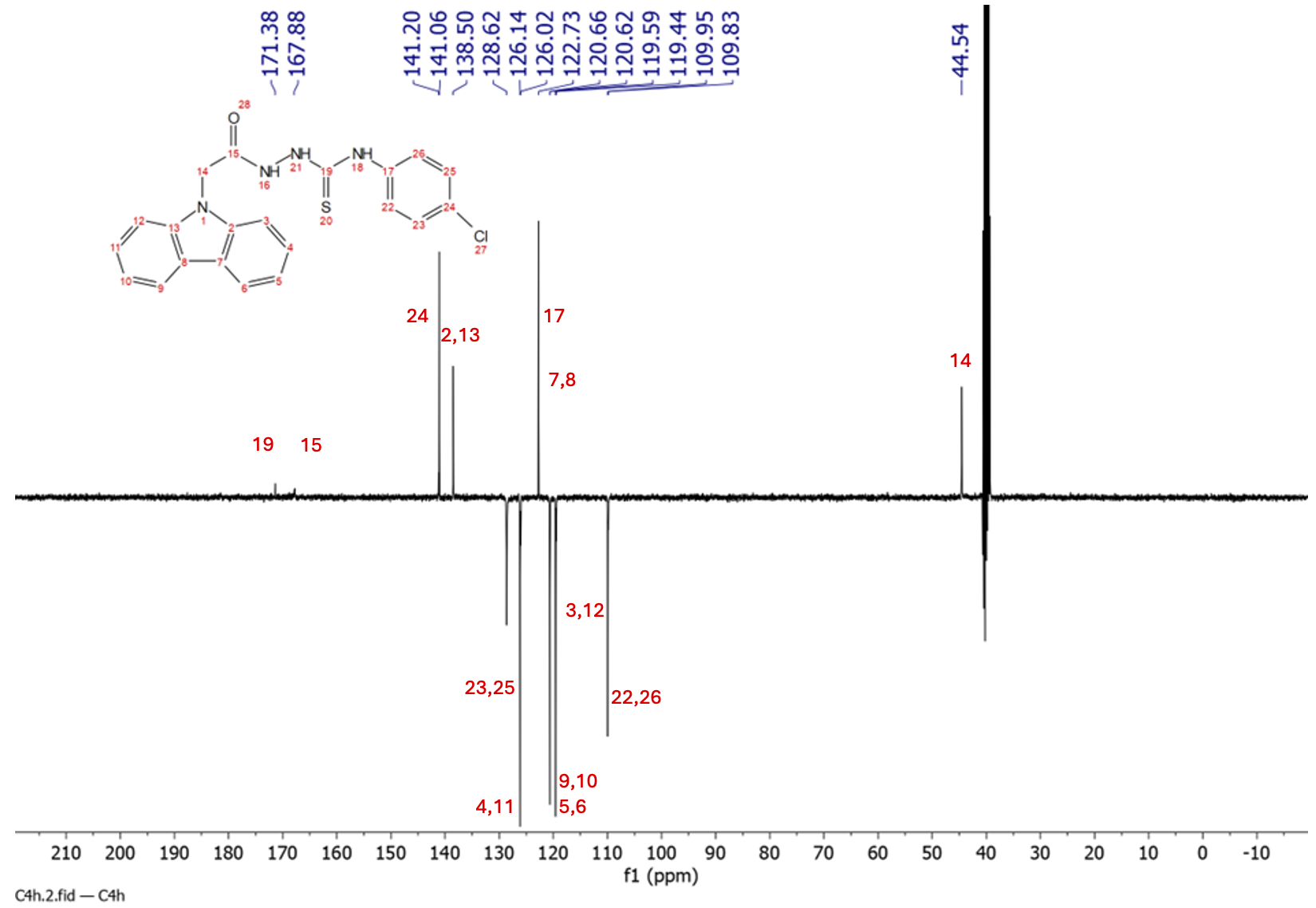


# Spectrums of Compound 4k

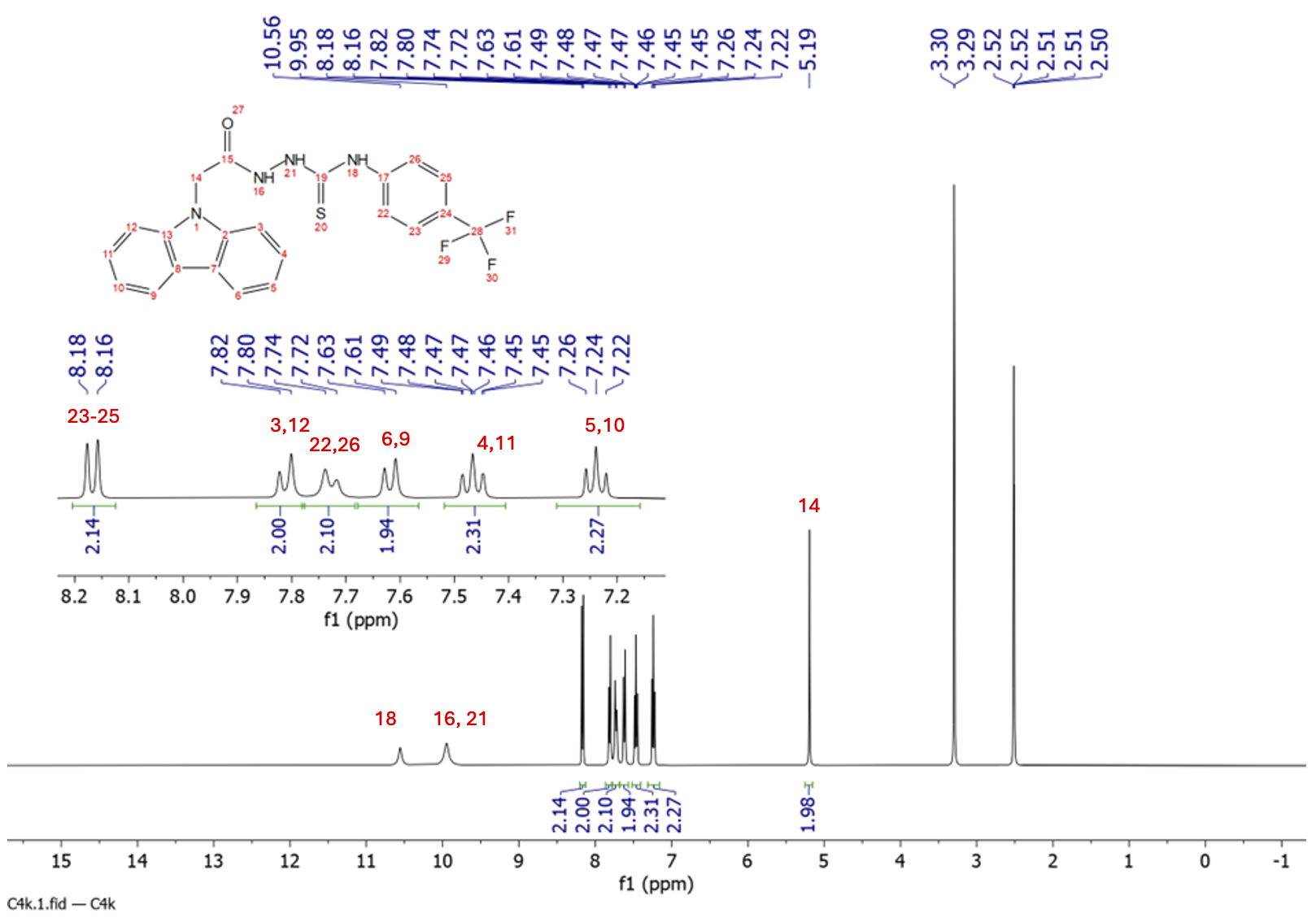


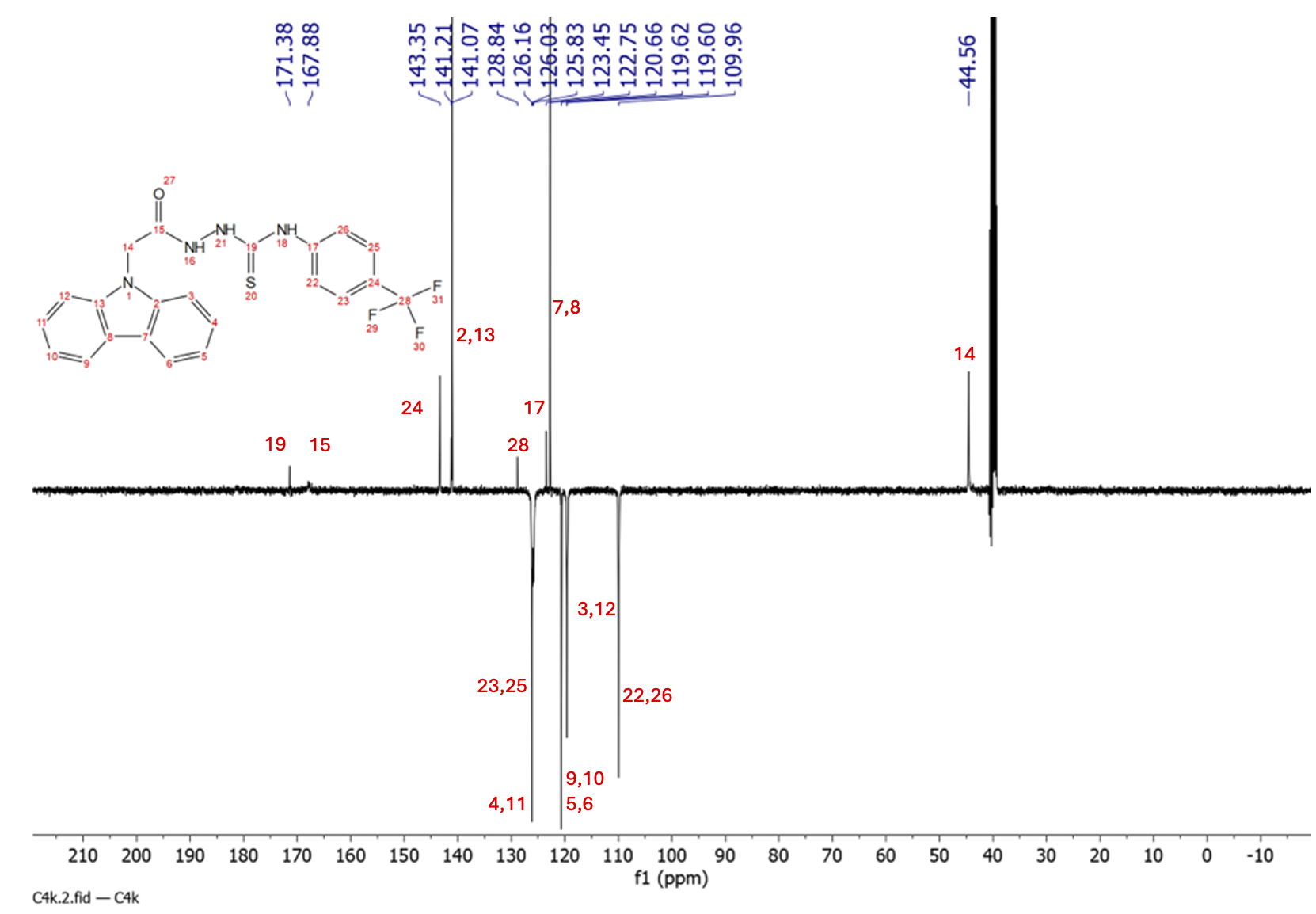


# Spectrums of Compound 4m

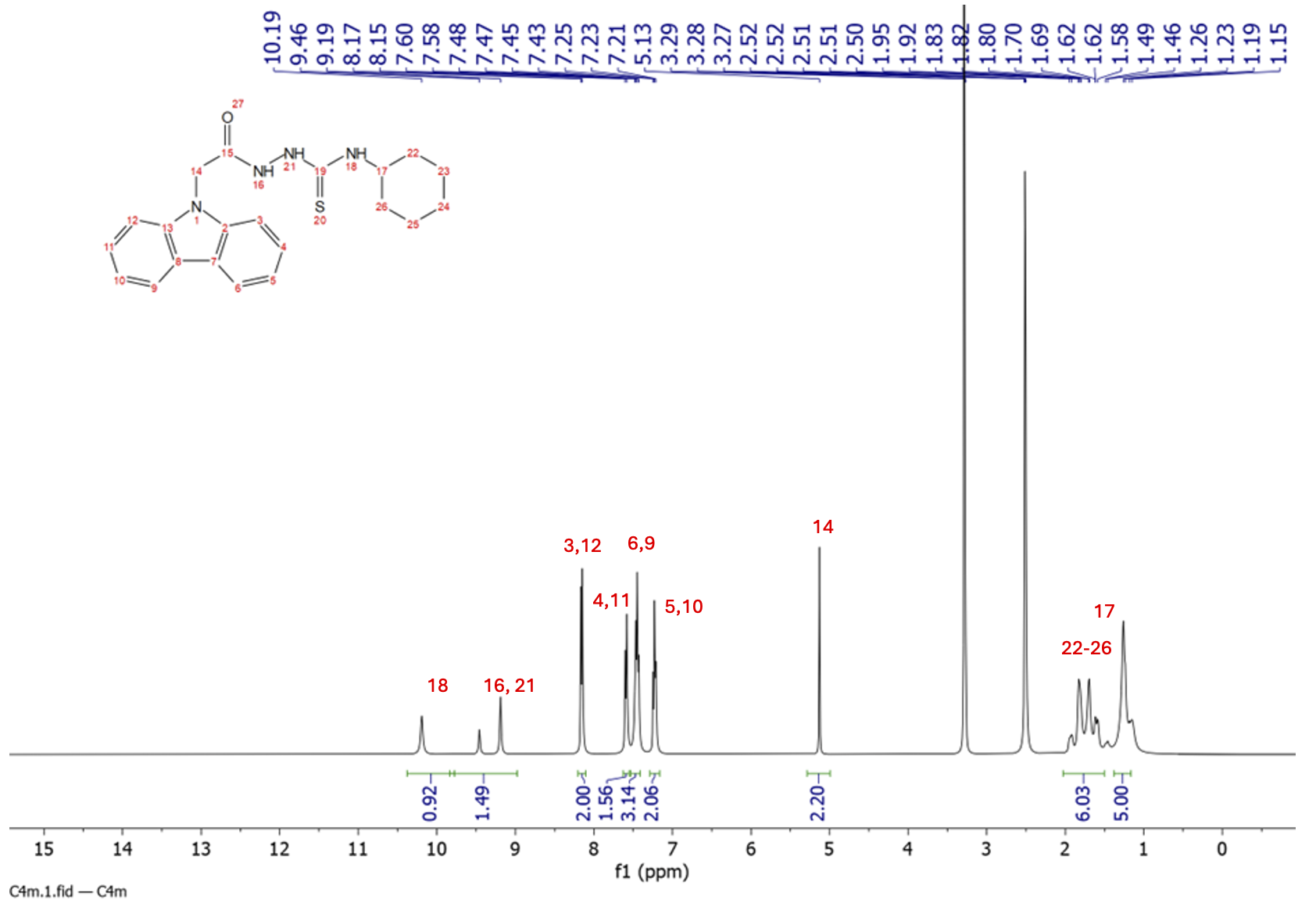


**
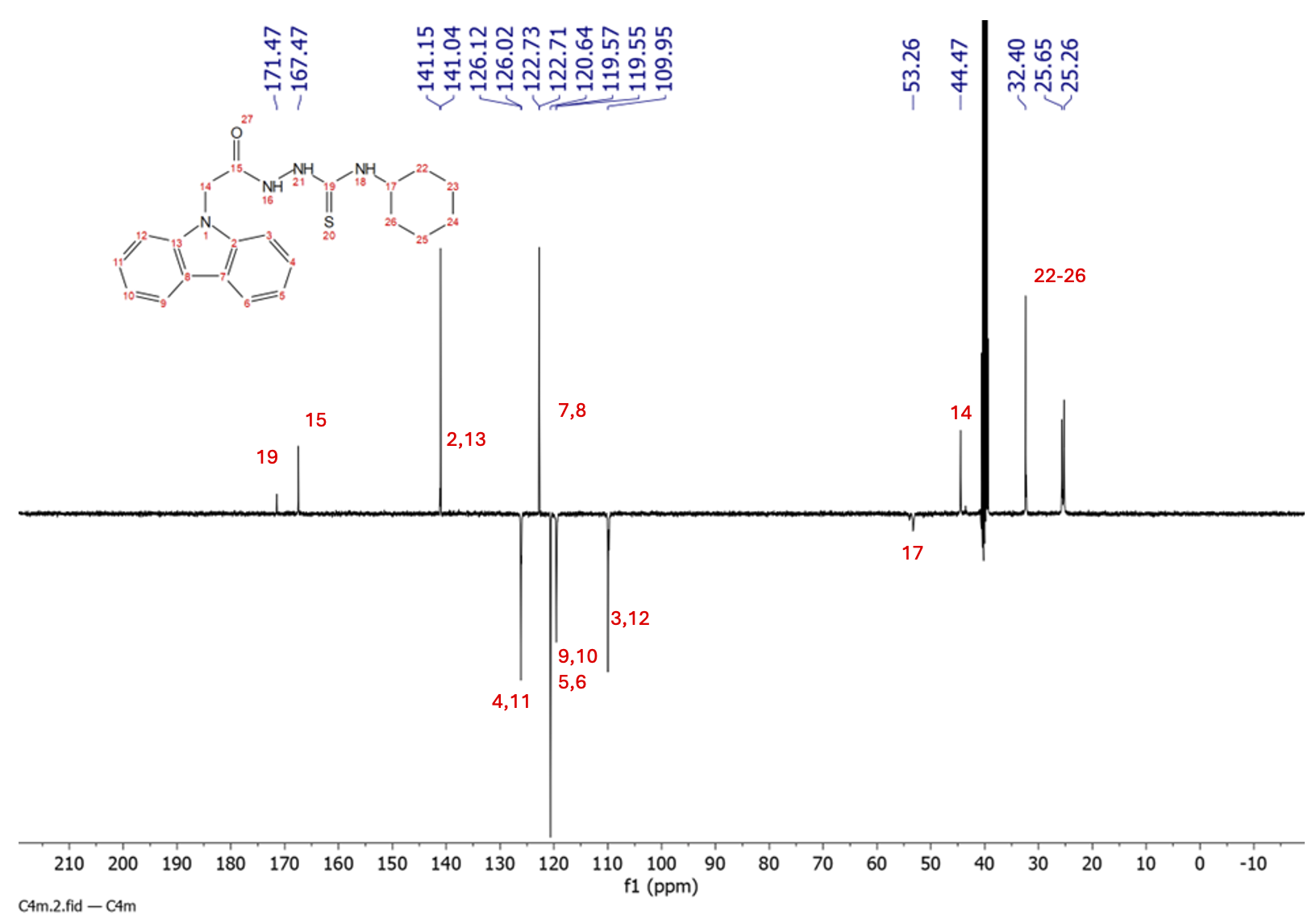
**

# Spectrums of Compound 4n

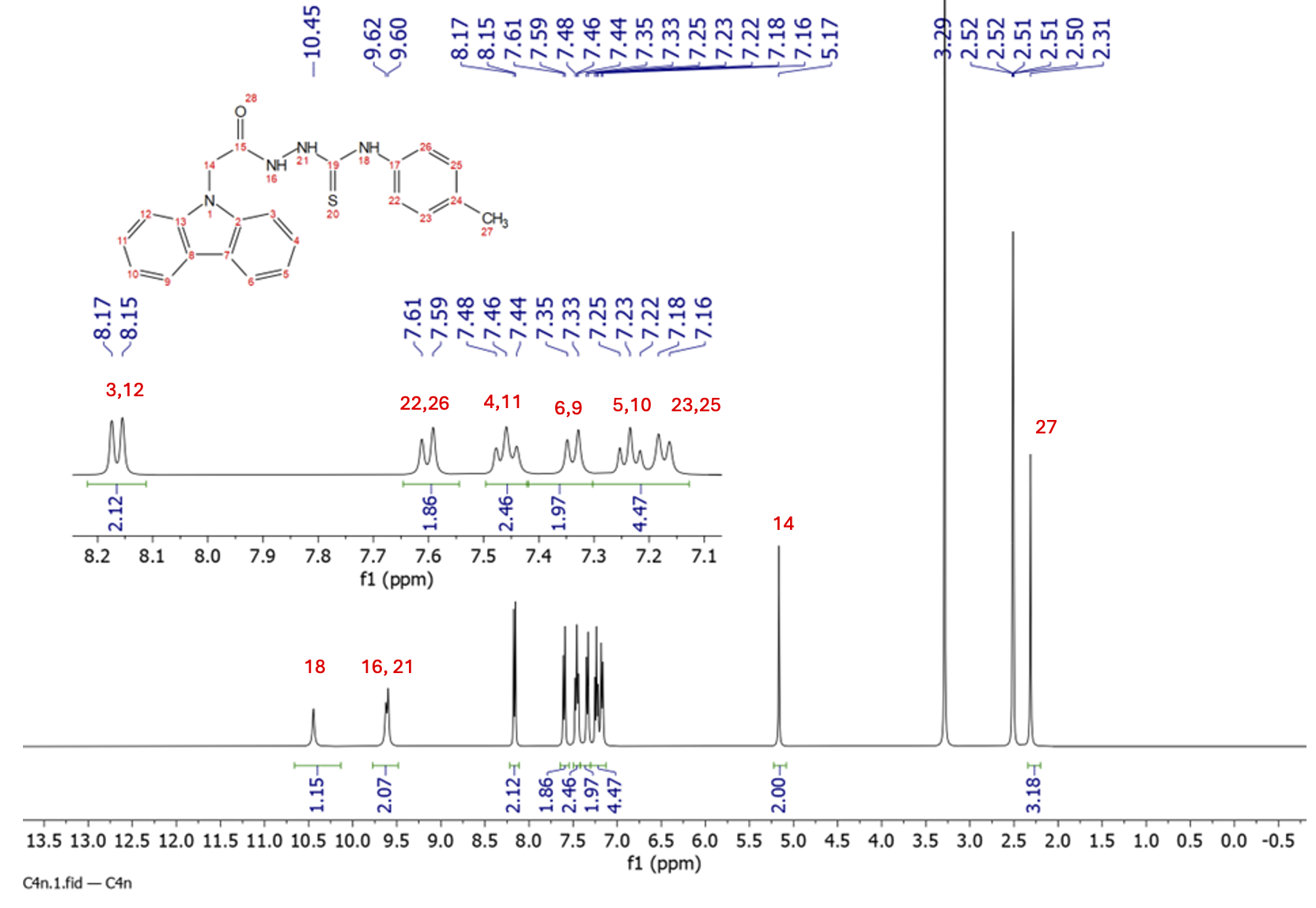


#
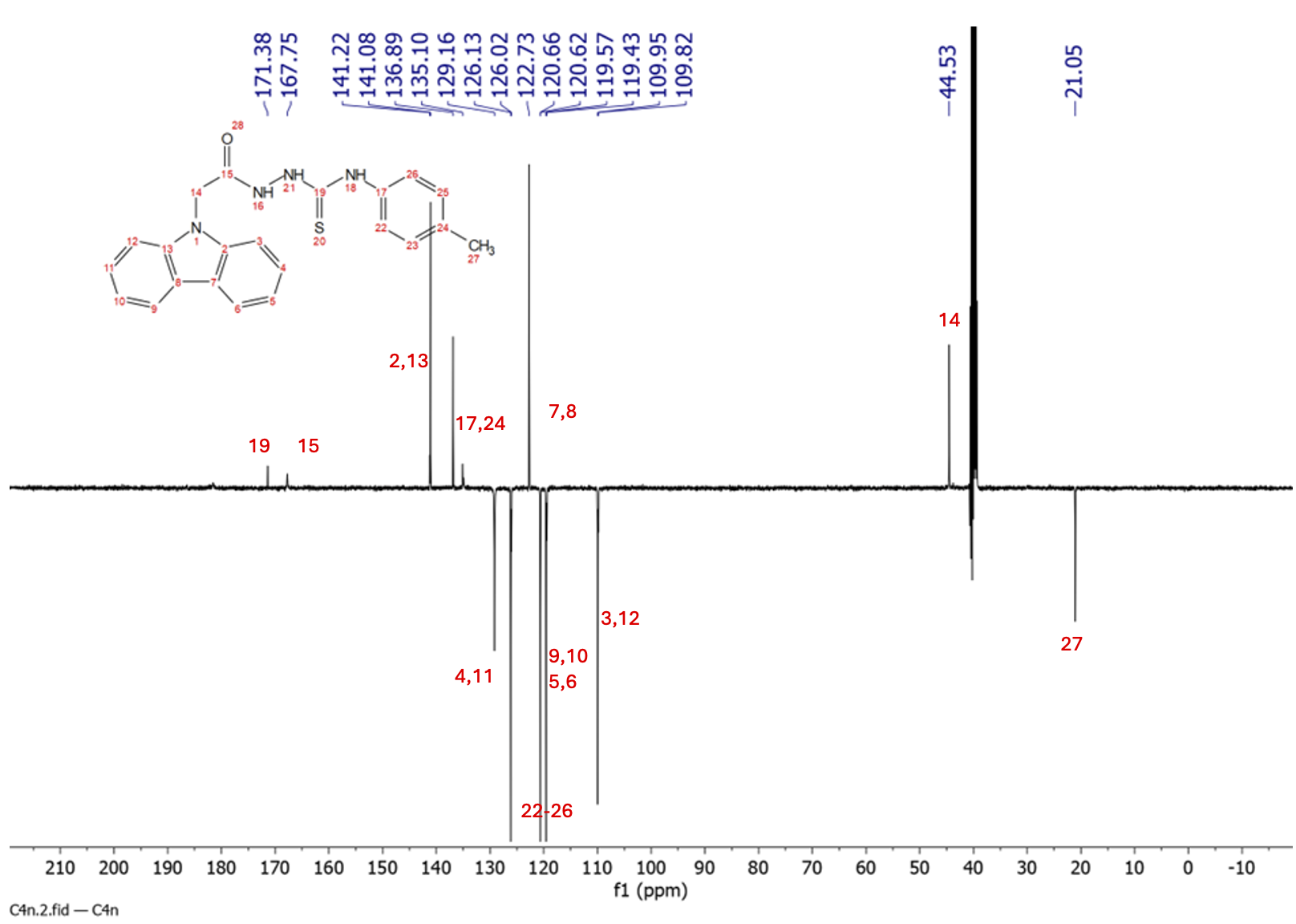


# Spectrums of Compound 4o

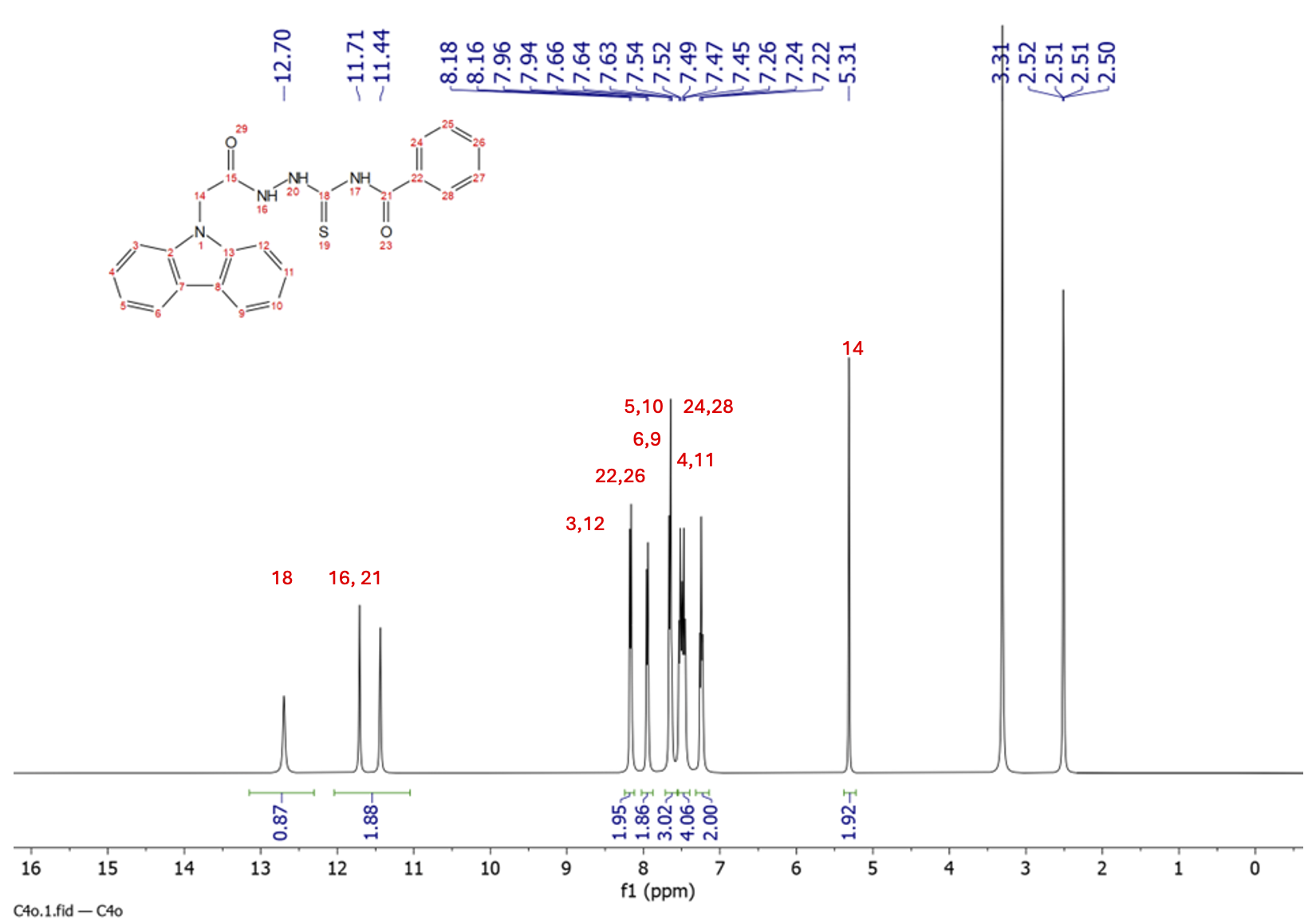


**
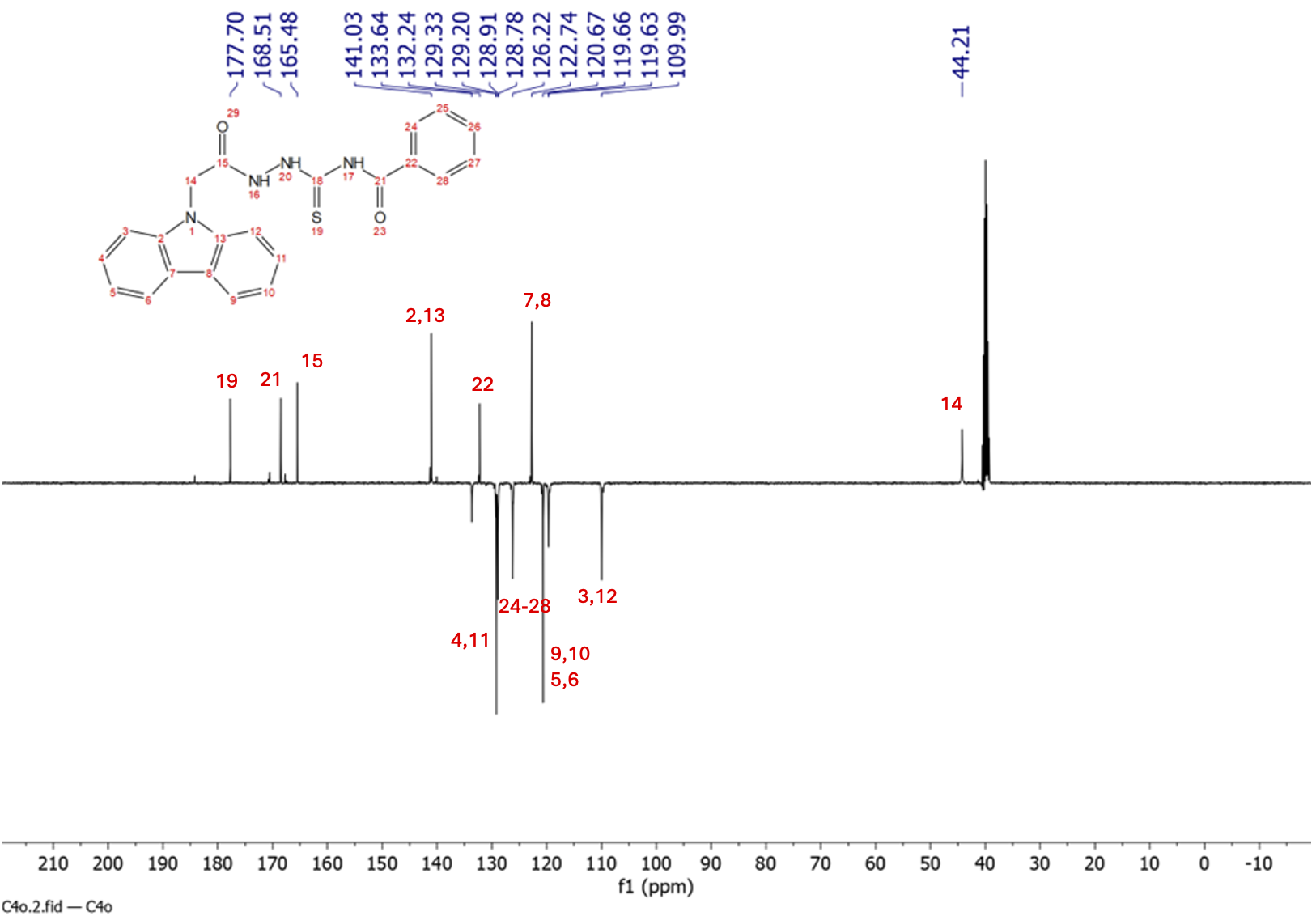
**

# Spectrums of Compound 4p

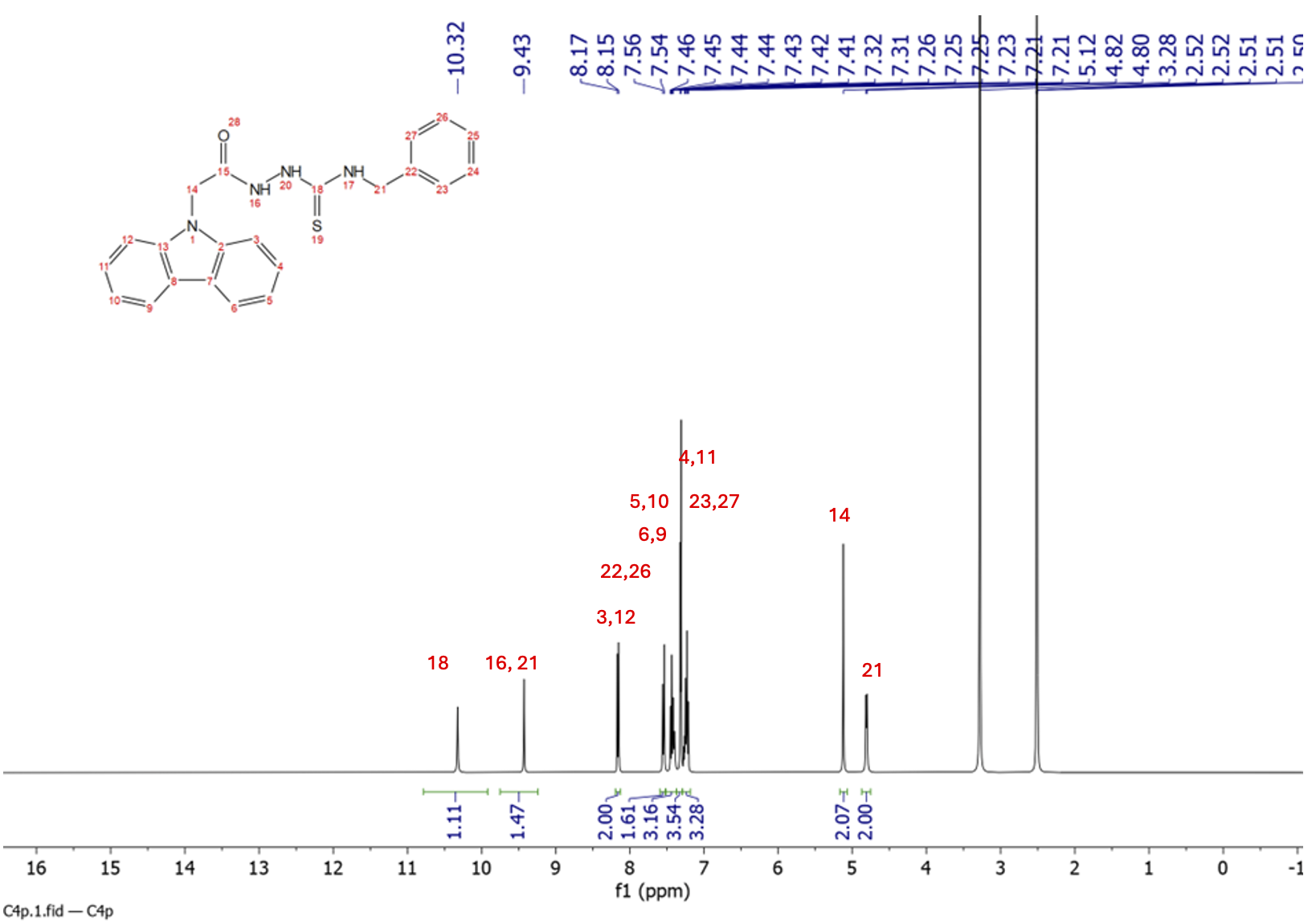


**
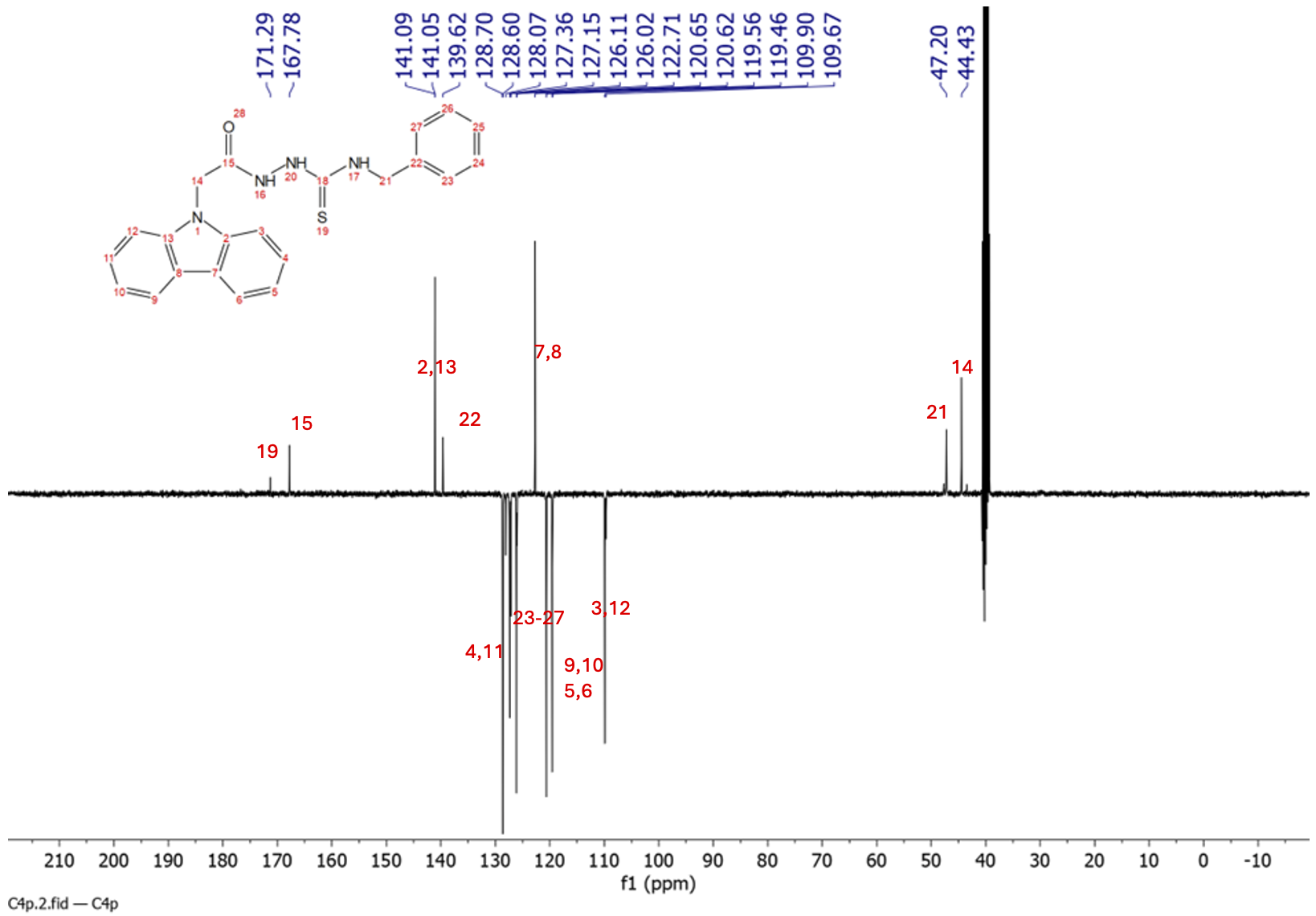
**

# Spectrums of Compound 4r

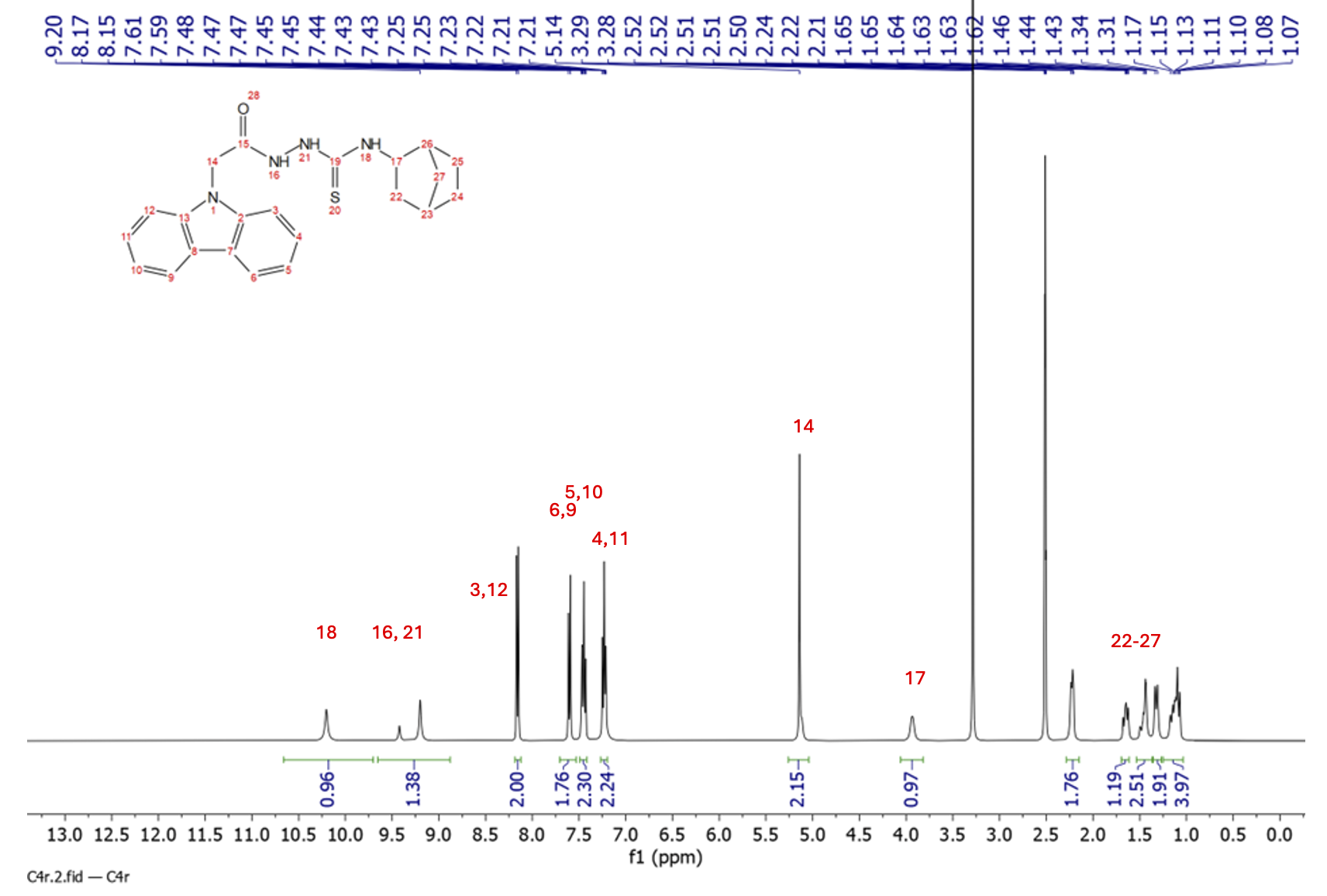


**
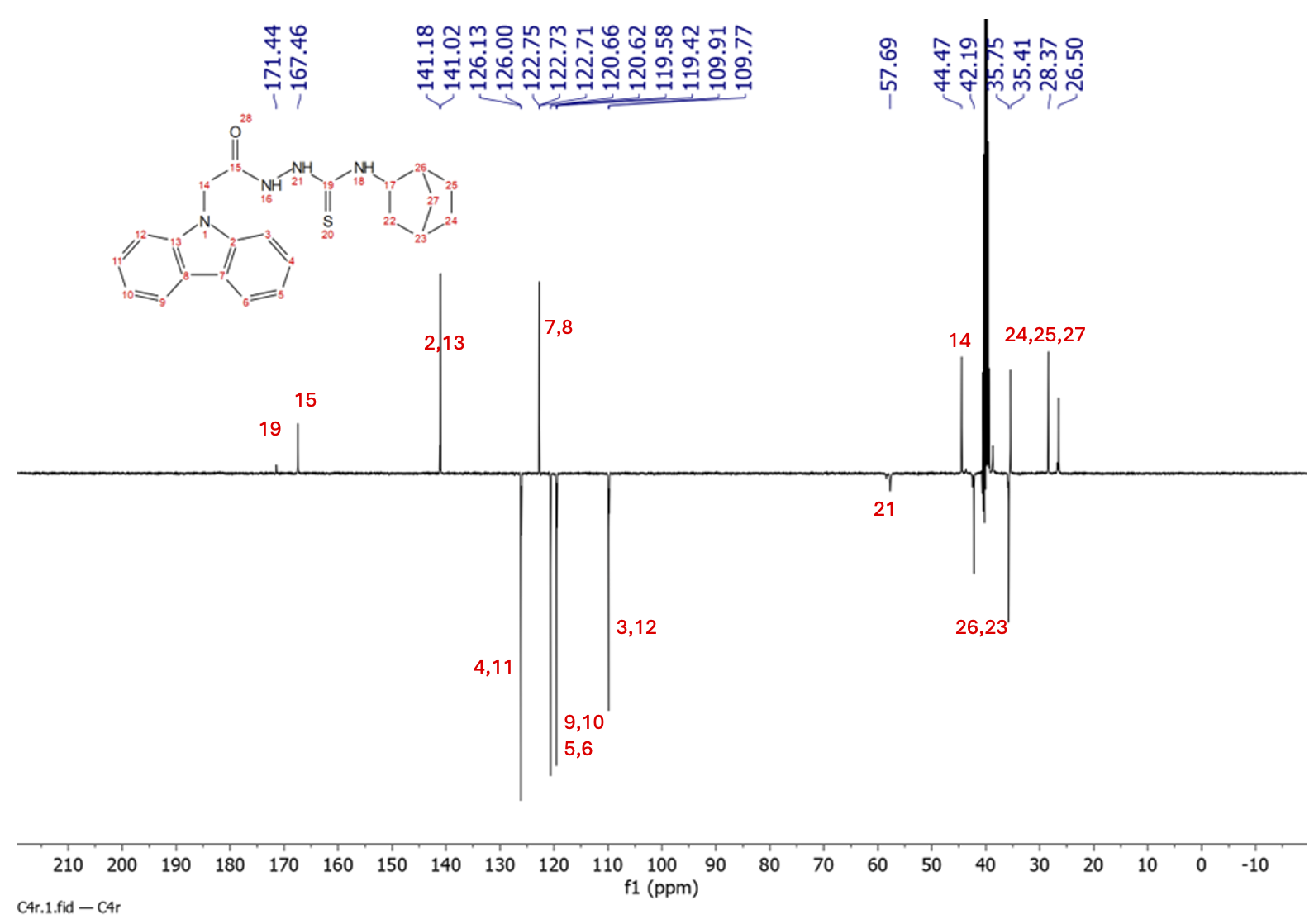
**

# Spectrums of Compound 4s

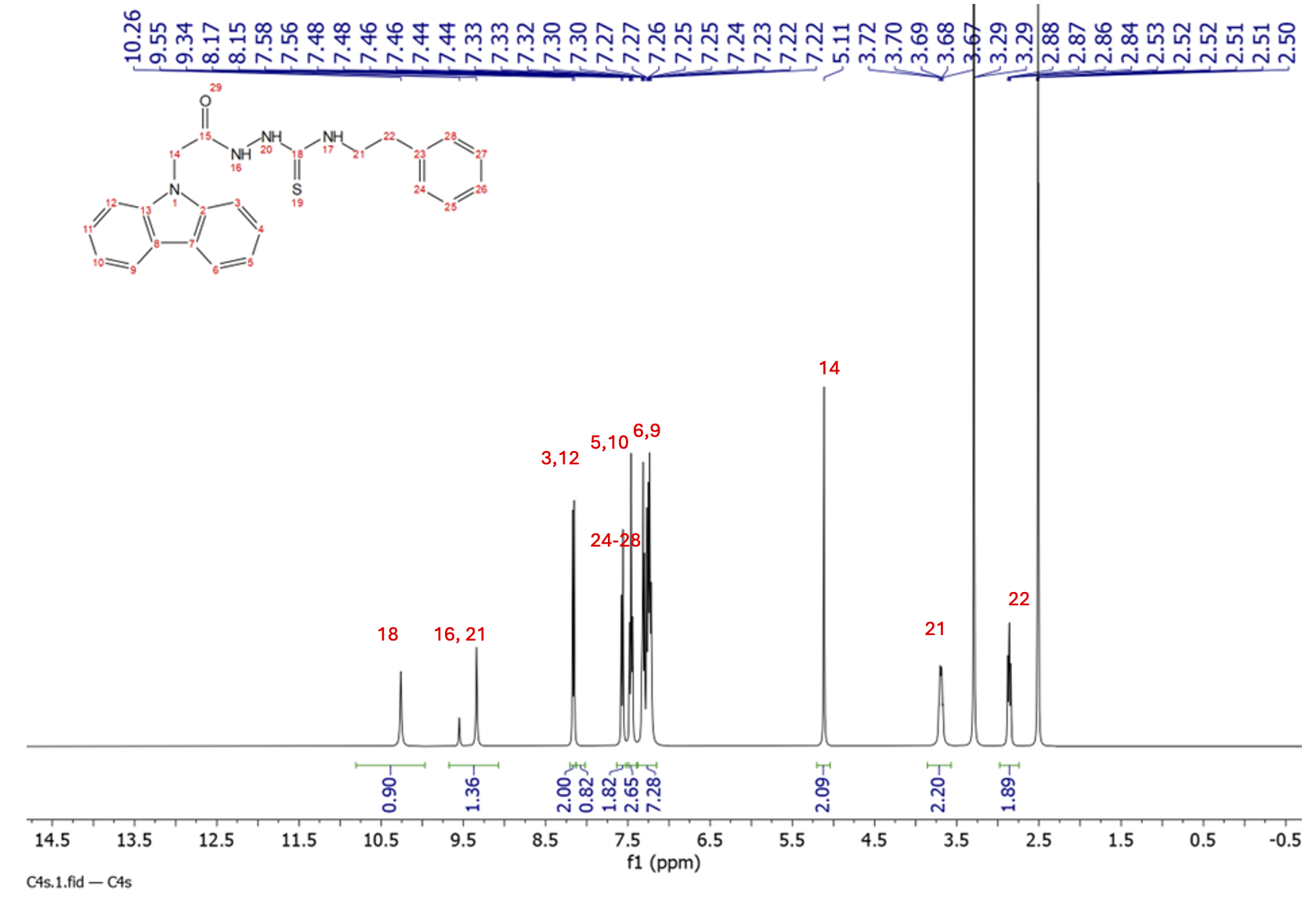


**
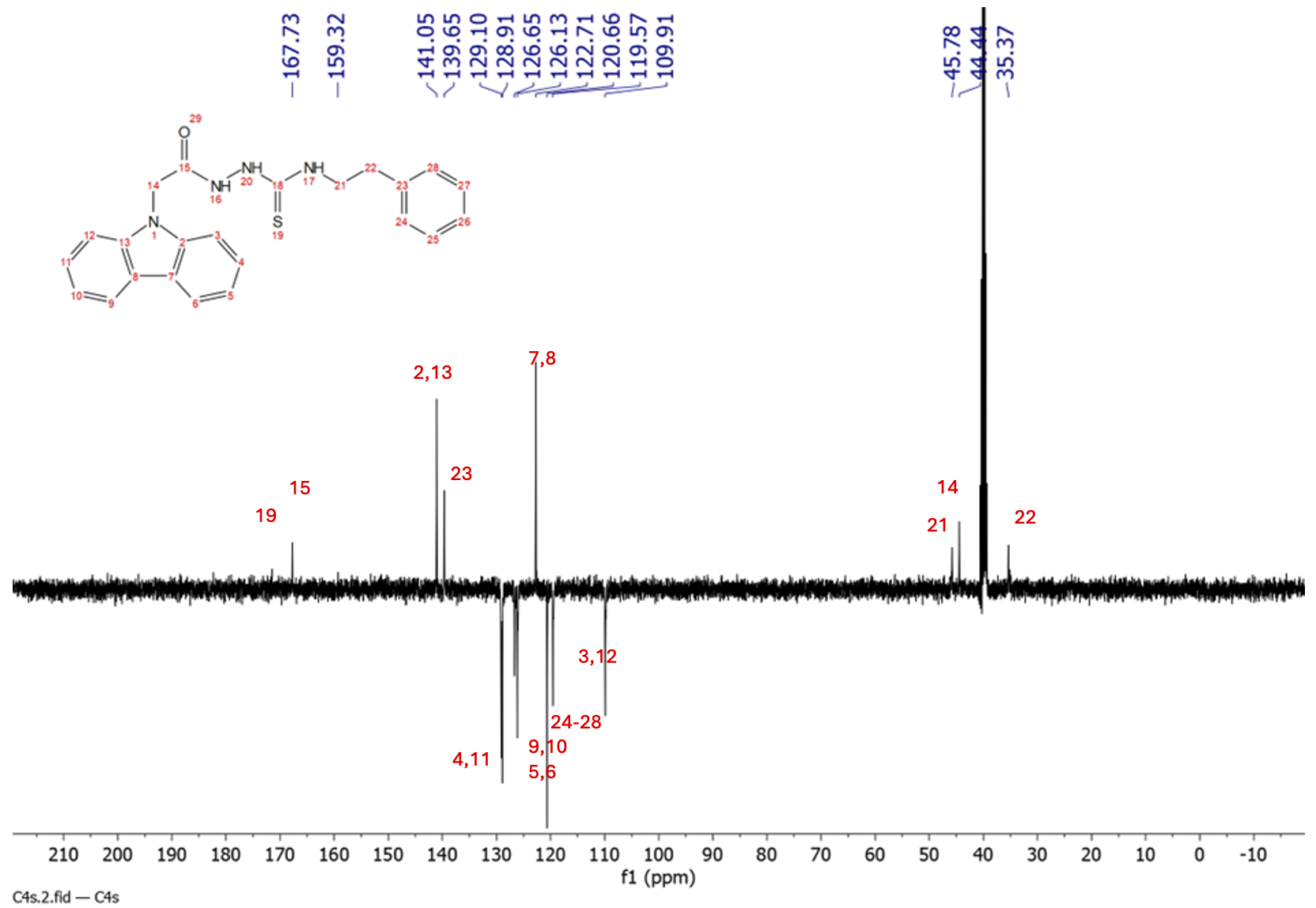
**

# Spectrums of Compound 4t

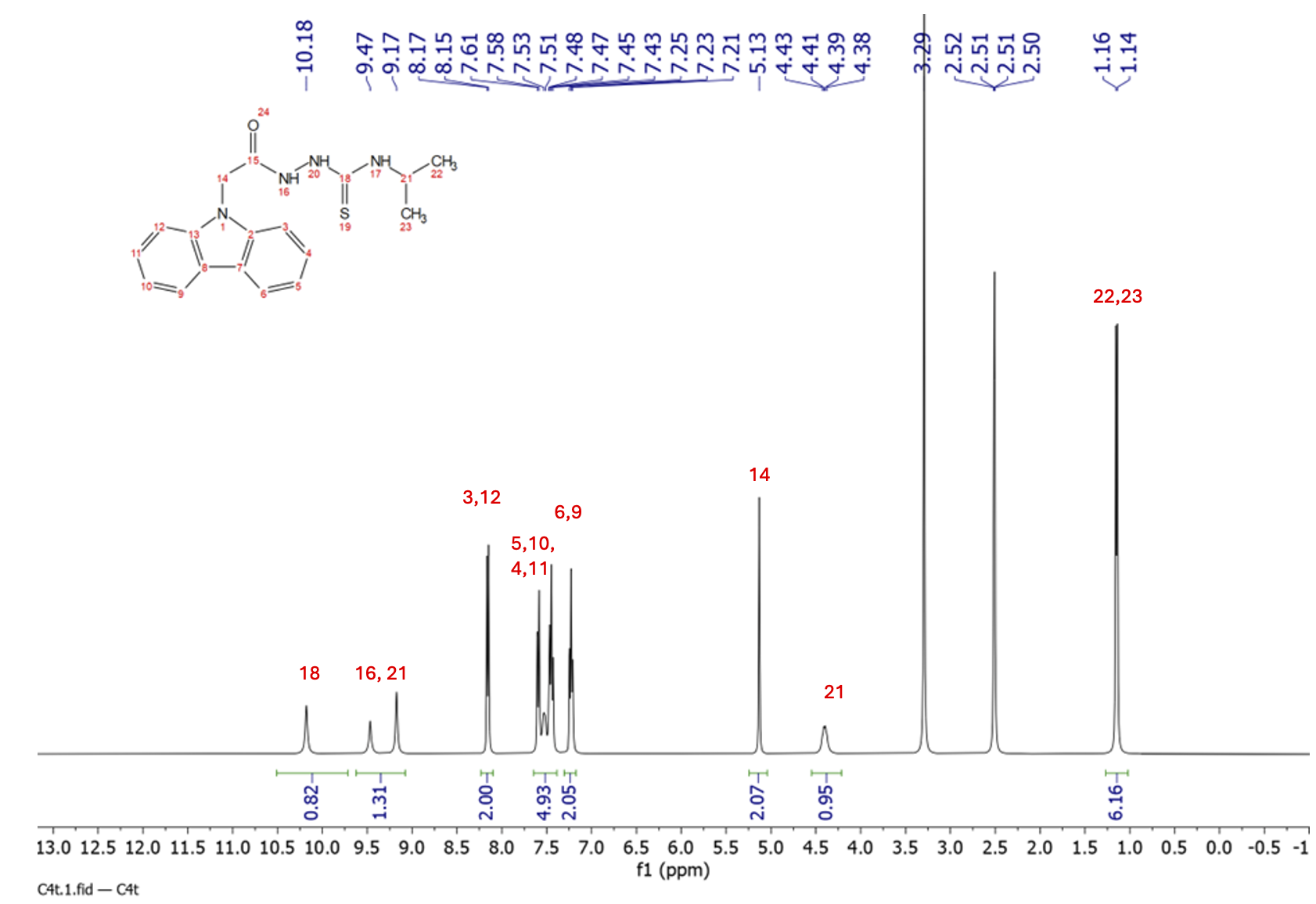


**
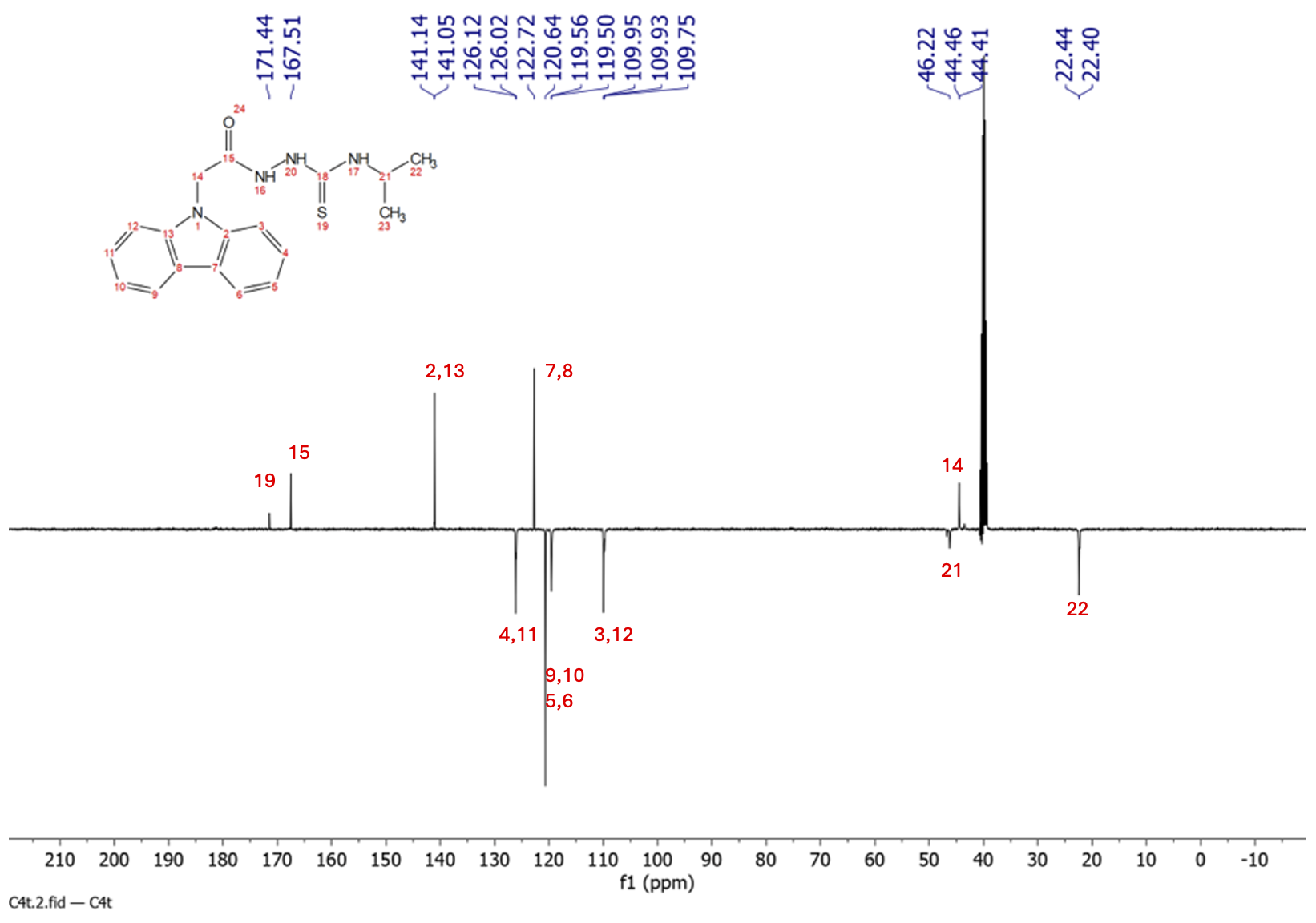
**

# Spectrums of Compound 4y

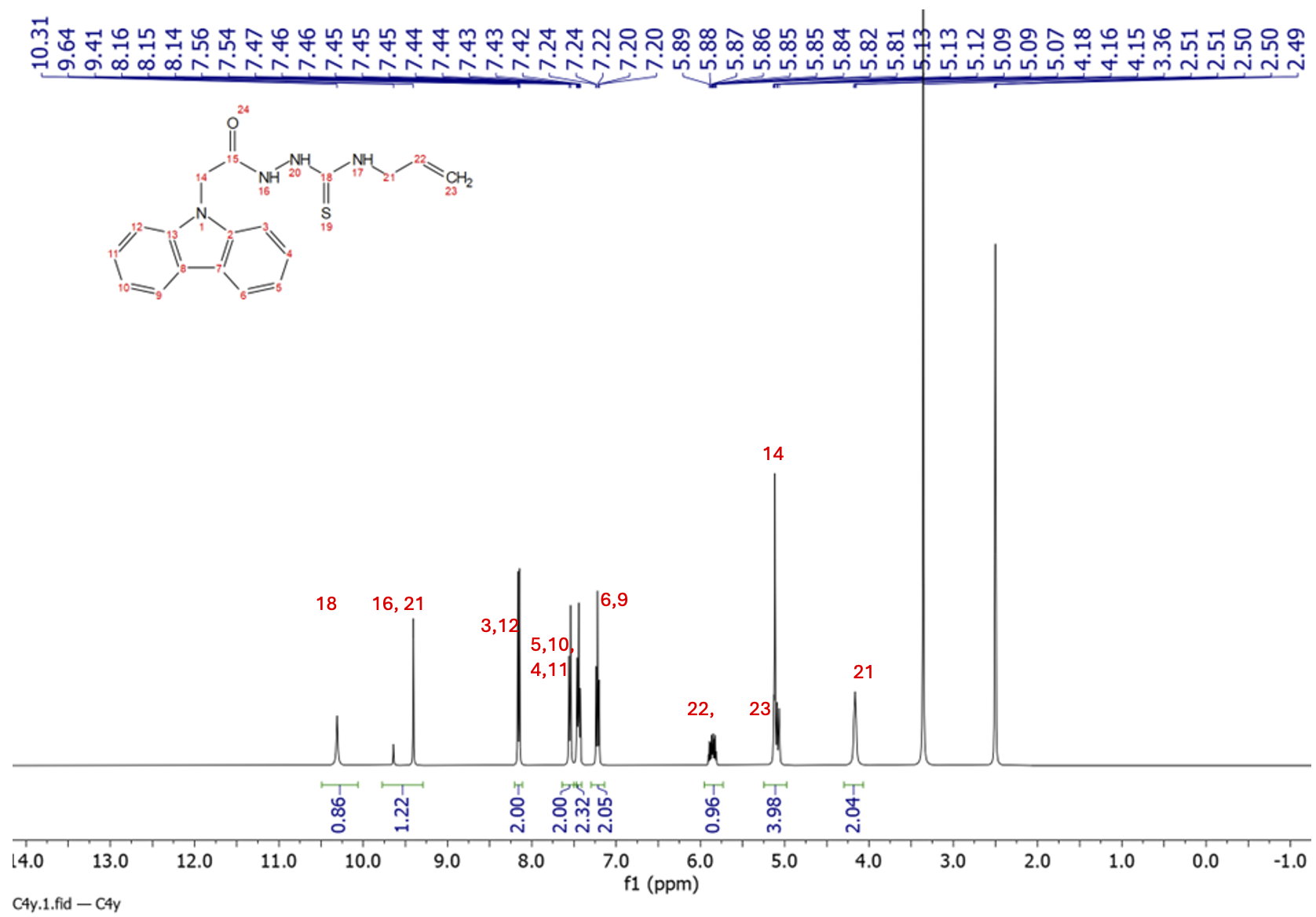


**
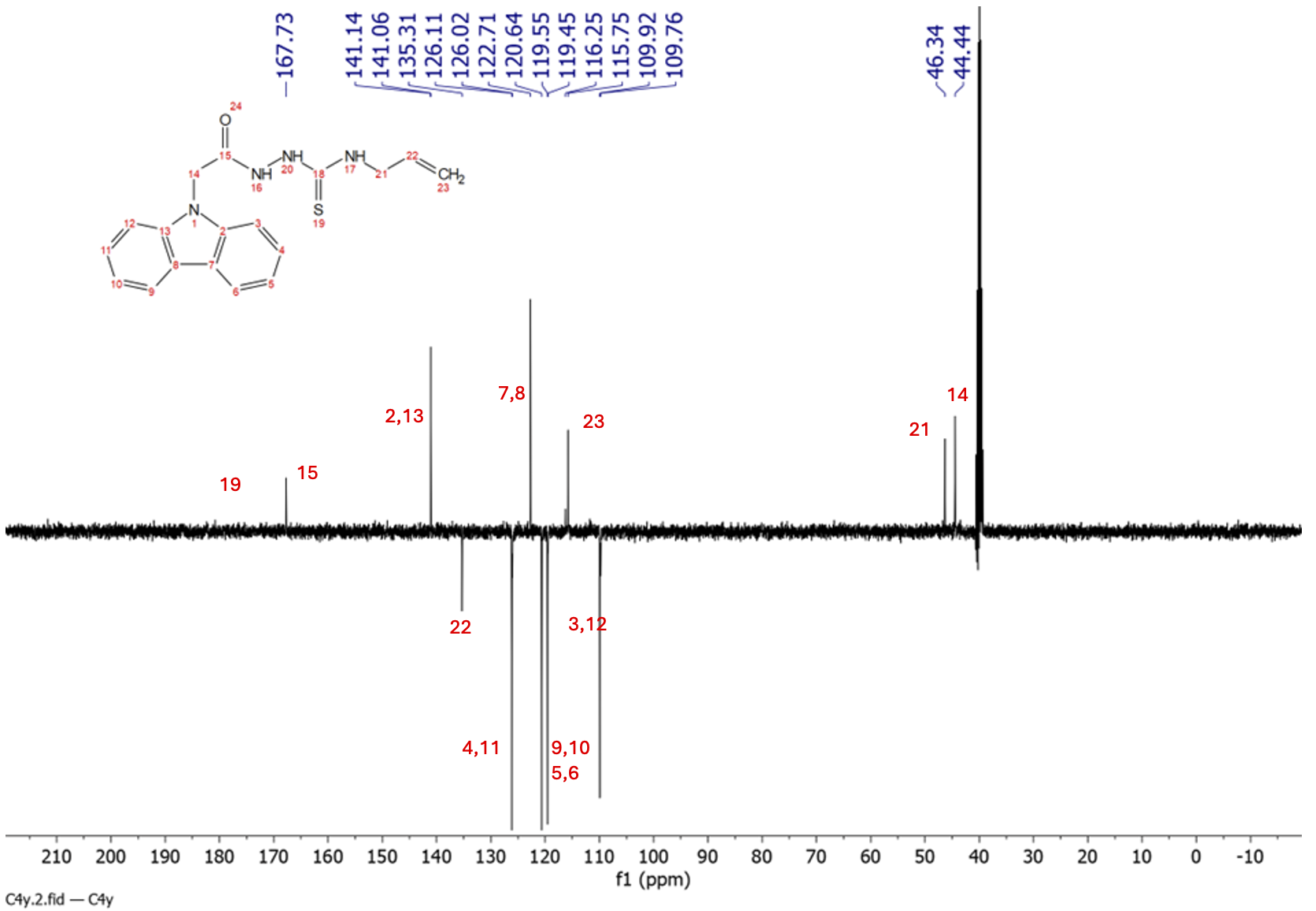
**

# Spectrums of Compound 4z

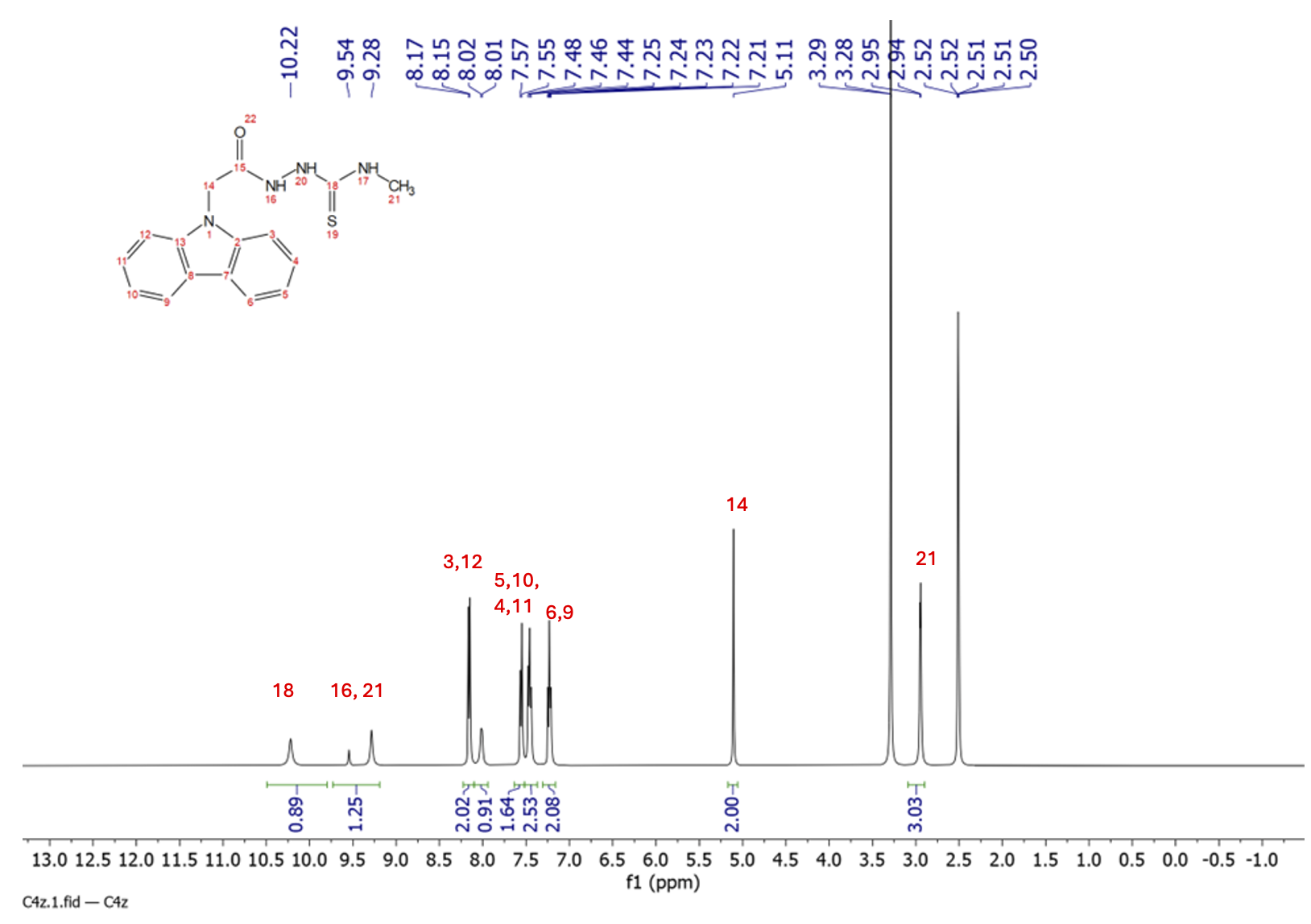


**
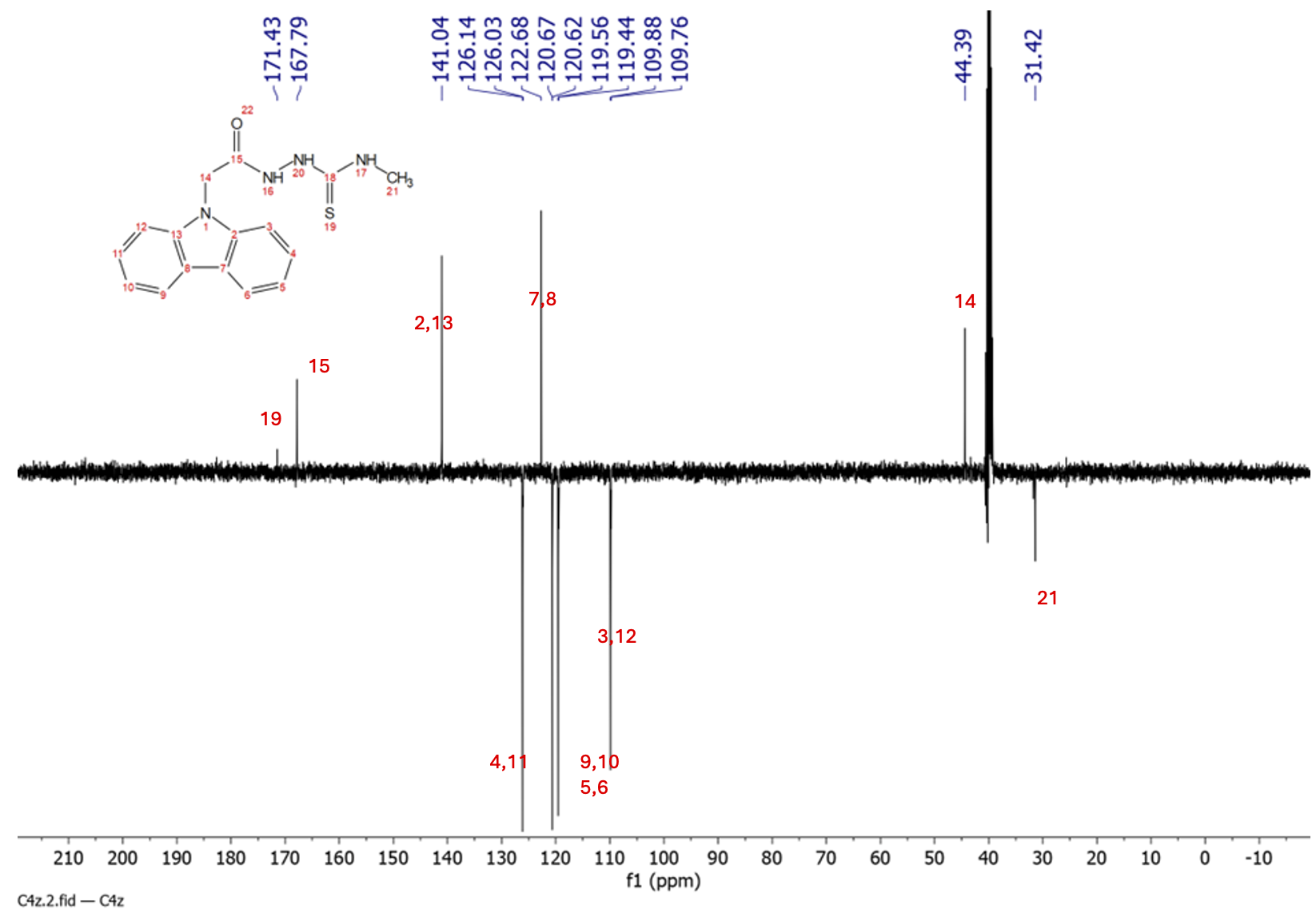
**

| Code | Binding Energy |
| --- | --- |
| 4a | -7.4 |
| 4b | -7.7 |
| 4c | -7.8 |
| 4d | -7.7 |
| 4e | -7.4 |
| 4f | -7.4 |
| 4g | -7.2 |
| 4h | -7.3 |
| 4k | -7.3 |
| 4m | -7.6 |
| 4n | -7.3 |
| 4o | -8.1 |
| 4p | -7.7 |
| 4r | -7.6 |
| 4s | -7.7 |
| 4t | -6.9 |
| 4y | -7.3 |
| 4z | -7.8 |

Table S1: Antibacterial/PDB: 4URO (A chain) docking score of compounds 4a-z

**Supplementary Figure 1.** To confirm inhibition of PI3K/AKT/mTOR pathway fold changes of mRNA expression levels of PI3K/AKT/mTOR pathway genes screened: PIK3CA (± 0.13) PIK3CB (± 0.08), PTEN (± 0.11), AKT1 (± 0.14), mTOR (± 0.10) in MCF-7 cell lines after treatment with inhibitor. All samples were normalized to the internal control β -actin, P < 0.05.

β -actin

Forward 5′ GACTTAGTTGCGTTACACCCTTTC-3′

Reverse 5′-TGCTGTCACCTTCACCGTTC-3′.

PIK3CA

Forward 5′-GGTTGTCTGTCAATCGGTGACTGT

Reverse 5′-GAACTGCAGTGCACCTTTCAAGC

PIK3CB

Forward 5′-TTGTCTGTCACACTTCTGTAGTT

Reverse 5′-AACAGTTCCCATTGGATTCAACA

PTEN

Forward 5′-GGTTGCCACAAAGTGCCTCGTTTA

Reverse 5′-CAGGTAGAAGGCAACTCTGCCAAA

AKT1

Forward 5′-TTCTGCAGCTATGCGCAATGTG

Reverse 5′-TGGCCAGCATACCATAGTGAGGTT

mTOR

Forward 5′-GCTTGATTTGGTTCCCAGGACAGT

Reverse 5′-GTGCTGAGTTTGCTGTACCCATGT
